# Semisynthesis of Functional Glycosylphosphatidylinositol‐Anchored Proteins

**DOI:** 10.1002/anie.202002479

**Published:** 2020-05-18

**Authors:** Renée F. Roller, Ankita Malik, Maria A. Carillo, Monika Garg, Antonella Rella, Marie‐Kristin Raulf, Bernd Lepenies, Peter H. Seeberger, Daniel Varón Silva

**Affiliations:** ^1^ Department of Biomolecular Systems Max Planck Institute of Colloids and Interfaces 14424 Potsdam Germany; ^2^ Institute of Chemistry and Biochemistry Freie Universität Berlin Arnimallee 22 14195 Berlin Germany; ^3^ Immunology Unit and Research Center for Emerging Infections and Zoonoses University of Veterinary Medicine Hannover Bünteweg 17 30559 Hannover Germany; ^4^ Institute for Parasitology, Center for infection Medicine University of Veterinary Medicine Hannover Bünteweg 17 30559 Hannover Germany

**Keywords:** GPI anchor, glycoproteins, glypiation, protein modifications, protein semisynthesis

## Abstract

Glypiation is a common posttranslational modification of eukaryotic proteins involving the attachment of a glycosylphosphatidylinositol (GPI) glycolipid. GPIs contain a conserved phosphoglycan that is modified in a cell‐ and tissue‐specific manner. GPI complexity suggests roles in biological processes and effects on the attached protein, but the difficulties to get homogeneous material have hindered studies. We disclose a one‐pot intein‐mediated ligation (OPL) to obtain GPI‐anchored proteins. The strategy enables the glypiation of folded and denatured proteins with a natural linkage to the glycolipid. Using the strategy, glypiated eGFP, Thy1, and the *Plasmodium berghei* protein MSP1_19_ were prepared. Glypiation did not alter the structure of eGFP and MSP1_19_ proteins in solution, but it induced a strong pro‐inflammatory response in vitro. The strategy provides access to glypiated proteins to elucidate the activity of this modification and for use as vaccine candidates against parasitic infections.

## Graphical abstract

**All together now**: An intein‐mediated one‐pot ligation enables the semisynthesis of glycosylphosphatidylinositol (GPI)‐anchored proteins. The process works efficiently with denaturated and folded proteins under diverse conditions. This method was applied to the synthesis of a natural GPI‐anchored protein from *Plasmodium berghei* that exhibits proinflammatory activity in vitro.

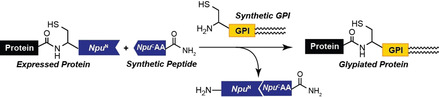

## Introduction

Posttranslational modification of proteins by the attachment of complex glycosylphosphatidylinositols (GPIs) is ubiquitous in eukaryotes and protozoa.^1^ GPIs consist of a phospholipid, a phosphoethanolamine unit, and a conserved *pseudo*‐pentasaccharide glycan core that is modified in a cell‐ and tissue‐specific manner.^2^ GPI‐anchored (“glypiated”) proteins (GPI‐APs) are functionally diverse, including hydrolytic enzymes, adhesion molecules, complementary regulatory proteins, receptors, and protozoan proteins, as well as cytokines and prion proteins.^1^, ^3^ The challenge of expressing GPI‐APs containing a single, defined glycolipid in eukaryotic cell lines and their purification is a major limitation for determining the effects of GPIs and their modifications on the structure and function of the anchored protein.

**Figure 1:**
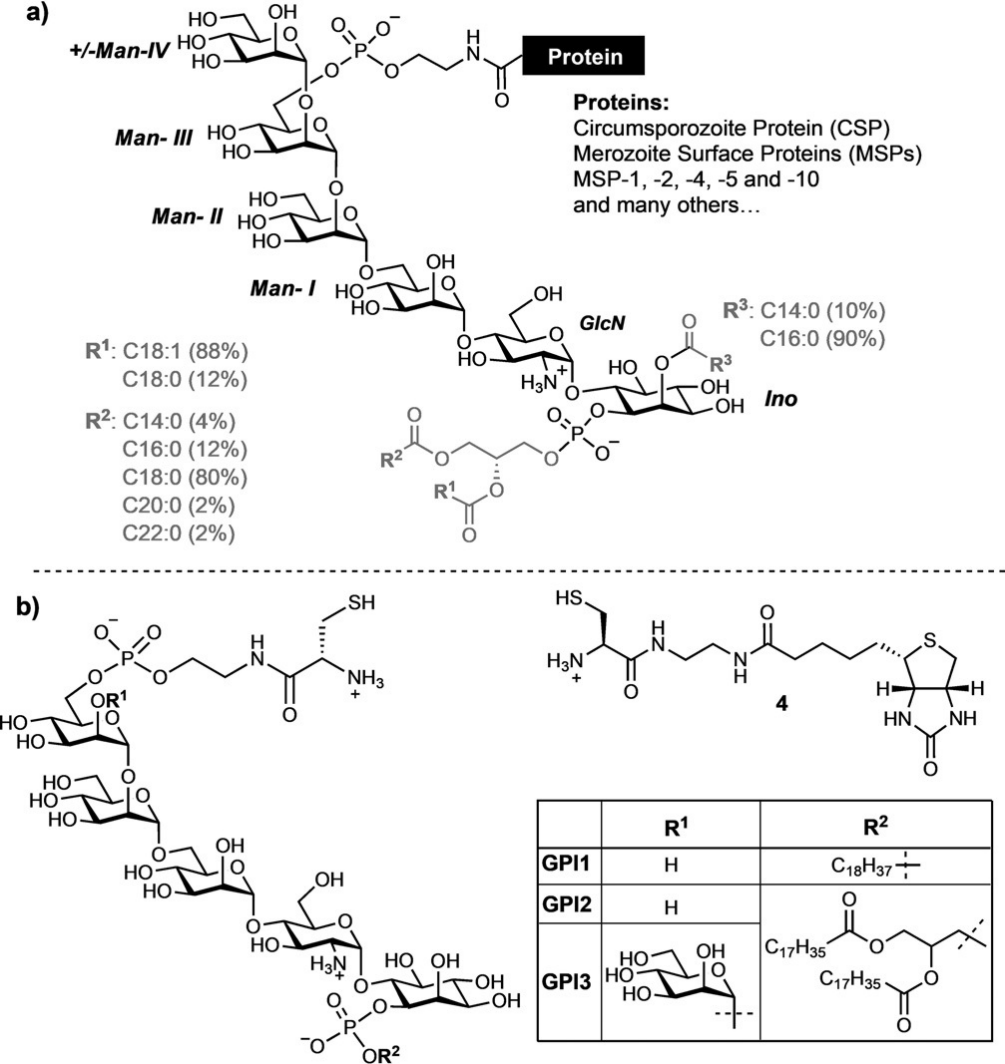
a) Lipid and glycan variability of GPIs anchoring *Plasmodium spp*. proteins. b) Structure of the cysteine‐containing GPIs and biotin used in this study.

Protozoan parasites cause severe human diseases such as leishmaniasis,^4^ Chagas disease^5^ and malaria.^6^ The cell membranes of these parasites contain large amounts of GPI‐APs that modulate the host immune response during infection.^7^ These proteins are of different size and complexity and include important vaccine candidates such as the surface variant glycoproteins from *Trypanosoma brucei*, mucins and mucin‐like proteins from *Trypanosoma cruzi*, the metalloprotease GP63 from *Leishmania donovani*, and the circumsporozite protein (CSP) on sporozoites and merozoite surface proteins (MSPs) from *Plasmodium falciparum*,^8^ among others.^9^ In *Plasmodium sp.,* GPI‐APs cover the parasite surface during all developmental stages and contribute to the survival of the parasites in the vector as well as to binding and invasion during the human liver and blood stages.8b The *Plasmodium* GPIs are very heterogenous in the lipid part and contain an additional mannose (Man‐IV) on the glycan core as the main modification (Figure 1 a).^11^

Peptides and proteins have been attached mainly to GPI analogues by coupling fully protected peptides and glycans,^12^ through Staudinger reaction and expressed‐protein ligation,^13^ and by transpeptidation with Sortase A.^14^ However, access to GPI‐APs containing a natural GPI limits the evaluation of the activity of these complex glycoconjugates. Herein, we describe a strategy to obtain GPI‐anchored proteins containing fully lipidated GPIs. The strategy uses the *Nostoc punctiforme* DnaE split intein (*Npu* ^N^/*Npu* ^C^)^10^ to generate protein thioester intermediates of the protein of interest that undergo a chemoselective reaction with synthetic cysteine‐containing GPI‐glycolipids in a one‐pot ligation (OPL).^15^ We established conditions for the reaction using enhanced green fluorescence protein (eGFP) as a protein model and show the semisynthesis of two naturally GPI‐anchored proteins: Thy1 (CD90) from thymocytes and MSP1_19_ from *Plasmodium berghei* ANKA (PbA). Moreover, we show that glypiation does not affect the structure of MSP1_19_ in solution but enhances the in vitro production of the pro‐inflammatory cytokines IL‐12 and TNF‐α by bone‐marrow‐derived dendritic cells.

## Results and Discussion

**Figure 2:**
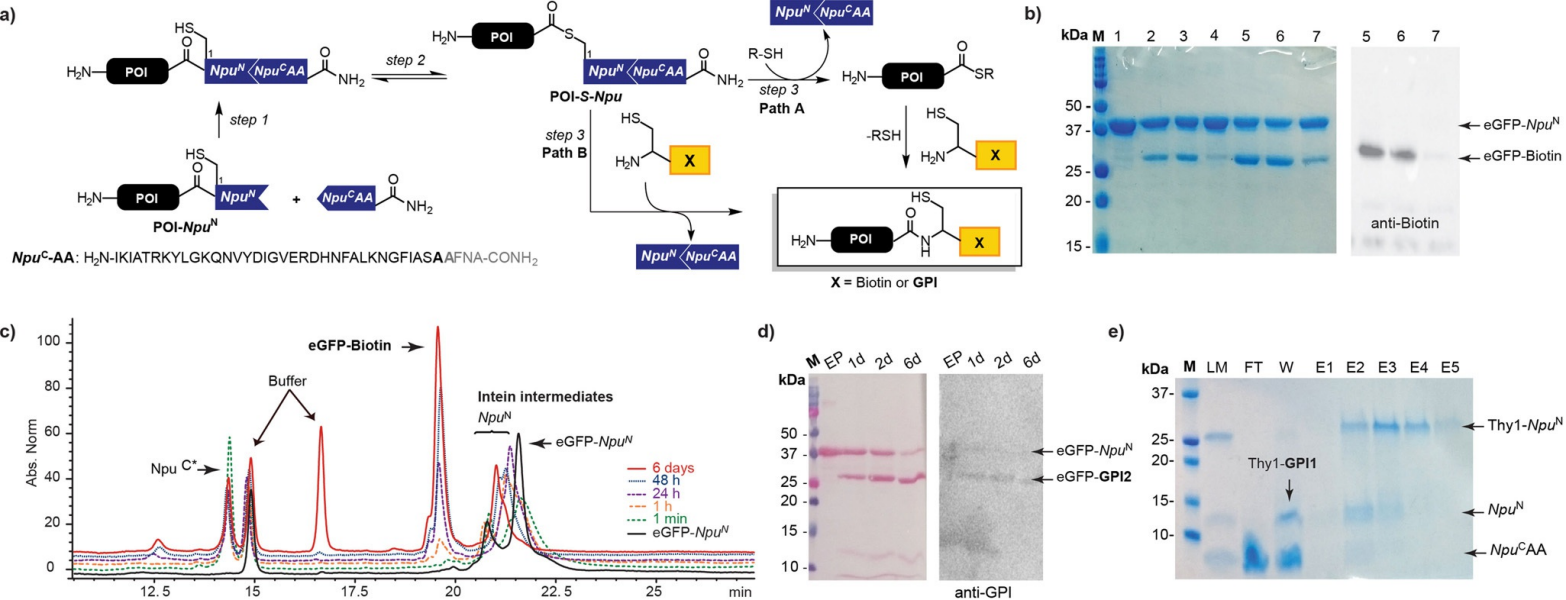
One‐pot ligation of eGFP with biotin. a) Scheme of the OPL reaction. b) Comparison of OPL with MESNA, MMP, and MMBA by SDS‐PAGE and western blot (eGFP‐Biotin detected with anti‐biotin antibody). M=Molecular weight marker; 1) GFP‐*Npu* ^N^; 2) OPL with MESNa, 1 h; 3) OPL with MMP, 1 h; 4) OPL with MMBA, 1 h; 5) OPL with MESNa, 1 d; 6) OPL with MMP, 1 d; 7) OPL with MMBA, 1 d. c) Kinetic study of OPL, monitored by RP‐HPLC (C4). d) SDS‐PAGE and western blot of the OPL of eGFP with **GPI2**; detection with Ponceau S staining and anti‐GPI antibody after 1,2 and 6 days. M=Molecular weight marker, EP=eGFP‐*Npu* ^*N*^. e) SDS‐PAGE of OPL between Thy1 and **GPI1**; purification by His‐Trap. M=Marker; LM=ligation mixture 6 d; FT=flow‐through; W=wash; E=elution fractions. MESNa=sodium 2‐mercaptoethanesulfonate, MMP=methyl 3‐mercaptopropionate.

Three fragments were necessary to obtain GPI‐APs by OPL using the *Npu* ^N^/*Npu* ^C^ split intein fragments: an expressed fusion protein of the protein of interest (POI) and the *Npu* ^N^ fragment (POI‐*Npu* ^N^), a synthetic *Npu* ^*C*^ fragment elongated with four amino acids at the C‐extein cleavage site, and a synthetic GPI glycolipid with a cysteine linked to the phosphoethanolamine unit (PEtN) on the GPI Man III unit (Figure 2 a).

The fusion proteins of *Npu* ^N^ with green fluorescence protein (eGFP‐*Npu* ^N^),13c *P. berghei* MSP1_19_ (MSP1_19_‐*Npu* ^N^), and Thy1 (Thy1‐*Npu* ^N^) were recombinantly expressed in *E. coli* either as soluble proteins or in inclusion bodies and were isolated by affinity chromatography using a HisTag attached at the C‐terminus of *Npu* ^N^ (for details see Supporting Information). The C‐terminal intein fragment coupled to the first four residues of the C‐Extein from DNA polymerase III from *Nostoc punctiforme* (*Npu* ^C^AA) was synthesized by the Fmoc strategy on Rink amide resin using microwave‐assisted solid‐phase peptide synthesis. The process provided the *Npu* ^C^ peptide in good amount and purity. However, the formation of aspartimide and a difficult separation from the product reduced the yield of the synthesis (Figure S1 in the Supporting Information).^15^ Two essential residues (Asn35 and Cys36) for the transfer and splicing in the natural intein were exchanged for Ala in this peptide, thereby de‐functionalizing the autocatalytic potential of the *Npu* ^C^ fragment for the corresponding transfer of the protein fused to the *Npu* ^N^ fragment.

**Scheme 1:**
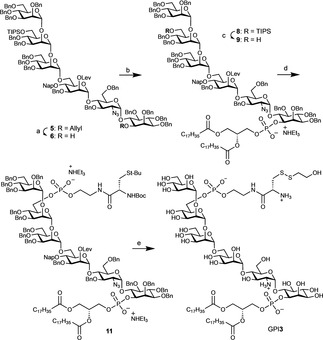
Synthesis of lipidated GPIs containing a cysteine residue. Conditions and reagents: a) PdCl_2_, AcOH, H_2_O, AcONa, 70 %. b) i. **7**,^16^ PivCl, pyridine; ii. I_2_, H_2_O 75 %. c) Sc(OTf)_3_, H_2_O, CH_2_Cl_2_/acetonitrile, 68 %. d) i. **10**,13b PivCl, pyridine; ii. I_2_, H_2_O, 70 %. e) i. H_2_N‐NH_2_‐AcOH, AcOH, pyridine, CH_2_Cl_2_; ii. Pd(OH)_2_/C, H_2_, CHCl_3_, MeOH, H_2_O; iii. TFA, anisole, Hg(OTFA)_2_, 0 °C, *iv*. β‐mercaptoethanol, H_2_O, 43 % (over 4 steps). Piv=pivaloyl, TfO=trifluoromethanesulfonate, TFA=trifluoroacetic acid.

The synthesis of **GPI1** and **GPI2**, which contain a cysteine residue, was completed using established methods.13b A convergent assembly of the glycan and two phosphorylations were used to install the lipid and a phosphoethanolamine coupled to cysteine. The synthesis of the GPI of *Plasmodium* proteins (**GPI3**, Figure 1 b) started with the preparation of the *pseudo*‐hexasaccharide **5** using two protected building blocks and a [4+2] glycosylation strategy (Scheme 1 and the Supporting Information).^16^, ^17^ Pseudo‐hexasaccharide **5** was deallylated with palladium chloride in acetic acid.^18^ A phosphitylation of *myo*‐inositol with H‐phosphonate **7** 16 and subsequent oxidation with iodine delivered the corresponding glycolipid **8**. Cleavage of the TIPS ether using scandium triflate and water to release the alcohol **9**, subsequent phosphitylation with the H‐phosphonate **10**,13b and careful oxidation with iodine and water gave the protected product **11**. Final removal of all protecting groups from **11** by treatment with hydrazine acetate, palladium‐catalyzed hydrogenolysis, and treatment with trifluoroacetic acid and with mercury trifluoroacetate delivered **GPI3** for the glypiation of proteins (Scheme 1).^19^

The one‐pot ligation (OPL) for C‐terminal protein modification involved three steps. The first step is the self‐assembly of the split‐intein fragments POI‐*Npu* ^N^ and *Npu* ^C^AA to give an active intein domain that in the second step induces an N‐ to S‐acyl shift to form the thioester POI‐*S*‐*Npu* ^N^ intermediate (Figure 2 a). Due to the lack of cysteine in *Npu* ^C^AA, the POI‐*S*‐*Npu* ^N^ intermediate cannot undergo an intramolecular transthioesterification with the C‐extein, but it reacts in the third step with a thiol to form the desired ligation product by one of two pathways (Figure 2 a). The first pathway (Path A) involves a transthioesterification of the intermediate POI‐*S*‐*Npu* ^N^ with a thiol additive, such as methyl 3‐mecaptopropionate (MMP), that is added to the reaction to form a protein thioester (POI‐SR) in situ. This thioester undergoes a second transthioesterification with a cysteine‐containing molecule (**GPI1**, **GPI2**, and **GPI3** or biotin‐**4**) and a subsequent irreversible S‐ to N‐acyl shift furnishes the desired product. In the second pathway, the product is formed through direct transthioesterification of POI‐*S*‐*Npu* ^N^ with the cysteine‐containing molecule and subsequent rearrangement to the ligation product (Figure 2 a). The OPL is technically an expressed‐protein ligation reaction, or native chemical ligation,^20^ that involves in situ formation of the protein thioesters mediated by an active split intein domain.

To establish the best conditions for the C‐terminal modification of proteins, we investigated the OPL between eGFP‐*Npu* ^N^ and biotin‐**4** using methyl 3‐mercaptopropionate (MMP), 4‐mercaptophenylacetic acid (MPAA), or sodium 2‐mercaptoethanesulfonate (MESNa) as a thiol reagent.^21^ SDS‐PAGE and western‐blot analysis of the reaction showed formation of the eGFP‐biotin product after one hour and increased formation of the product after 24 h when using MMP or MESNa (Figure 2 b and Figure S6). A kinetic study of this OPL monitored by RP‐HPLC showed a fast association of the intein fragments, the formation of intermediates to generate the active protein thioester, and the appearance of the ligation product in small quantities already after one minute. To achieve high protein conversion and high product yields, incubation over several hours or days was required (Figure 2 c). These results were in good agreement with a fast association of the intein and formation of the eGFP‐SR thioester and a slower ligation to the desired product. They showed also that a capture of the intein intermediate from the N‐ to S‐acyl shift with cysteine‐containing compound would deliver the product without the need for additional thiol reagents. However, since GPIs are difficult to synthesize and are available only in limited amounts, we performed the OPL using the cysteine‐GPIs as limiting reagents.

Glypiation of eGFP via the OPL strategy was investigated with **GPI1**, which contains a single lipid chain, using the conditions for the ligation with biotin‐**4**. OPL with this glycolipid was extended to several days to increase conversion. The glypiated eGFP‐**GPI1** product was detected by western blot using an anti‐GPI antibody. LC–MS analysis confirmed the formation of the product and the applicability of the strategy to glypiated eGFP (Figures S7 and S8). We next evaluated the OPL for glypiation of eGFP with **GPI2**, which bears a natural lipid with two alkyl chains shows low solubility in the ligation buffer. β‐Octylglucopyranoside (22 mm) was added as a surfactant to the buffer to increase the solubility of **GPI2** and the OPL was accomplished using the same reaction time and concentrations used with **GPI1**. SDS‐PAGE analysis of the ligation at different times showed a slower conversion of the eGFP‐SR thioester and lower yield formation of the eGFP‐**GPI2** product comparing to the OPLs with biotin‐**4** and **GPI1** (Figure 2 d). This slower conversion may be a result of the low solubility of **GPI2** and possible formation of micelles even in the presence of the surfactant.

Next, we performed the glypiation of the hydrophobic Thy1 (CD90) protein by OPL. The solution structure of GPI‐anchored glycoprotein Thy1 is affected by glypiation.^22^ The GPI of this protein was the first fully elucidated GPI and has as sialic acid containing trisaccharide glycan side chain on Man‐I.^23^ In addition to the GPI anchor, Thy1 has three N‐glycosylation sites and is naturally a mixture of more than 120 glycoforms. The biological function of Thy1 is not completely understood; however, it has been associated with T‐cell proliferation and different types of cancer.^24^ The influence of glycosylation and the GPI anchor on Thy1 activity remains unknown.^22^

Expression of the Thy1‐*Npu* ^N^ fusion protein in *E. coli* yielded the protein in inclusion bodies requiring high concentrations of urea for solubilization (8 m) and purification (>2 m). OPL with biotin‐**4** using the same conditions used for the ligations with eGFP, but including 2 m urea for keeping the Thy1‐*Npu* ^N^ fusion protein in solution, allowed the intein fragments to fold, thereby inducing the formation of the activated intein domain and generation of the corresponding protein thioester. OPL with Thy1 proceeded similarly to that with eGFP; the Thy1‐SR thioester formed fast, the ligation product Thy1‐biotin appeared after a few minutes, and high conversion was achieved after several days (Figure S9).

Thy1 was ligated to **GPI1** using the conditions established with biotin‐**4**. After OPL for seven days, the Thy1‐**GPI1** product was purified. The unreacted fusion protein Thy1‐*Npu* ^N^ and cleaved *Npu* ^N^ carrying a C‐terminal HisTag were retained on the HisTrap column and were eluted with imidazole (Figure 2 e). The product and the *Npu* ^C^AA intein fragment lacking the tag were not retained in the HisTrap column and were separated either by dialysis or using SEC on a Superdex 30 column. The purification was monitored by SDS‐PAGE (Figure 2 e) and the product was analyzed by western blotting and LC–MS (Figure S10). The hydrophobic Thy1‐**GPI1** ligation product was obtained, but MS characterization proved challenging as various complex mixtures of adducts and fragmentation products were observed.

Encouraged by the results obtained coupling eGFP and Thy1, OPL glypiation of the C‐terminal fragment of MSP1 (MSP1_19_), one of the most abundant and highly conserved GPI‐APs in *Plasmodium sp*. was undertaken.^25^ MSP1 is a cysteine‐rich protein stabilized by multiple disulfide bonds and is expressed as an approximately 200 kDa protein precursor. The protein is linked to the GPI via cysteine and it is an essential protein for the invasion and survival of the parasite in the host.^26^ MSP1 undergoes two proteolytic cleavages leaving the C‐terminal fragment (MSP1_19_) anchored to the membrane via the GPI.^27^ MSP1_19_ remains on the membrane during the red blood cell infection and is internalized by endocytosis during the ring‐stage into the vacuolar system, where it localizes at the food vacuole,25f a unique organelle in *Plasmodium* for degradation of hemoglobin and target for multiple antimalarial drugs.^28^

OPL between MSP1_19_ *‐Npu* ^N^ and biotin‐**4** using MMBA as thiol reagent resulted in fast formation of the MSP1_19_‐biotin product as determined by western blotting and LC–MS. SDS‐PAGE monitoring of the process showed almost complete consumption of the fusion protein after four days (Figure S11). MSP1_19_‐*Npu* ^N^ was ligated next to the synthetic **GPI3** using OPL using the conditions established to obtain eGFP‐**GPI2** (Figure 2 a and the Supporting Information).^29^ The ligation progressed in a similar manner to the ligation with biotin‐**4** and delivered the product MSP1_19_‐**GPI3** with high conversion of the fusion protein after five days (Figure S12).

**Figure 3:**
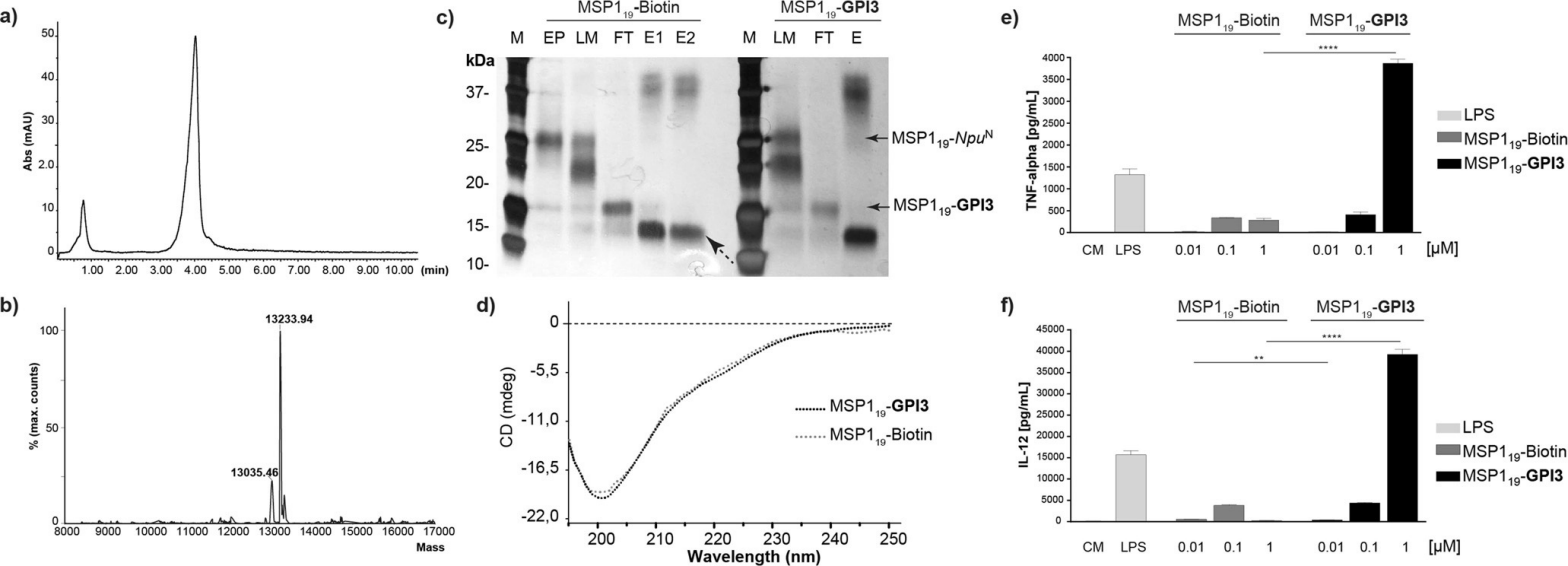
Characterization of GPI‐anchored proteins. a) RP‐HPLC chromatogram of purified MSP1_19_‐**GPI3**. b) Deconvoluted ESI‐MS spectrum of MSP1_19_‐**GPI3**. c) SDS‐PAGE after His‐Trap purification of the preparative synthesis of MSP1_19_‐Biotin and MSP1_19_‐**GPI3**. M=molecular weight standard, FT=flow through; EP=MSP1_19_‐*Npu* ^N^, LM=ligation mixture; E=elution fractions; dotted arrow shows the intein. d) CD spectra of MSP1_19_‐Biotin and MSP1_19_‐**GPI3**, e, f) Production of TNF‐α (e) and IL‐12 (f) by BMDCs stimulated with the MSP1_19_‐Biotin and MSP1_19_‐**GPI3**. CM=Culture medium.

OPL was scaled up for eGFP‐*Npu* ^N^ and MSP1_19_‐*Npu* ^N^ with biotin‐**4**, **GPI1**, and **GPI3** to obtain eGFP‐biotin, eGFP‐**GPI1**, MSP1_19_‐biotin, and MSP1_19_‐**GPI3** to investigate the effects of glypiation on the structure and biological activity of the proteins. These ligations were carried out under optimized conditions using 10 μm MSP1_19_‐*Npu* ^N^, 30 μm *Npu* ^C^AA, 20 mm TCEP solution, and 70 mm MMBA in Tris buffer at pH 7.2 containing octylglucoside (22 mm) for ligation with **GPI3**. The MSP1_19_‐biotin and MSP1_19_‐**GPI3** products were isolated using HisTrap and SEC columns and were analyzed by SDS‐PAGE, MALDI MS, and LC–MS (Figure 3).

Possible structural changes on the proteins during OPL or by the glypiation were analyzed by circular dichroism (CD).^30^ Previous CD studies showed different profiles with and without glypiation that were attributed to a structural change in the protein.^22^, ^31^ The CD spectra of eGFP‐Biotin, eGFP‐**GPI1**, and eGFP‐OH exhibit a minimum at 210 nm that corresponds with the β‐sheet folds in the β‐barrel structure reported for this protein (Figures S15).^32^ Similarly, the CD spectra of MSP1_19_‐biotin and MSP1_19_‐**GPI3** showed a profile corresponding to a random coil structure that is in good agreement with previously reported CD spectra for unmodified MSP1_19_ containing two epidermal growth factor (EGF) like domains (Figure 3 d).^33^ CD analysis indicates that OPL and glypiation did not affect the structure of eGFP and MSP1_19_ in solution, which is a requirement for the immunogenicity of this protein.^34^

Isolated GPIs and GPI‐anchored proteins from parasites induce a strong proinflammatory response and the production of cytokines by macrophages.^35^ This type of response is characteristic in patients with severe malaria that show enhanced levels of IL‐6, TNF‐α, and IL‐12 correlating with parasitemia.^36^ Thus, a proinflammatory response is considered important for protection against malaria and for clearance of the parasites, but may also contribute to malaria‐associated pathology such as cerebral malaria.^37^ The proinflammatory activity of MSP1_19_‐biotin and MSP1_19_‐**GPI3** was tested in vitro and compared to bacterial lipopolysaccharide (LPS), a strong proinflammatory stimulator. Bone‐marrow‐derived dendritic cells (BMDCs) from mice were cultured in the presence of Iscove's modified Dulbecco's medium (CM, negative control), 1 μg mL^−1^ lipopolysaccharide (LPS, positive control), or each of the modified MSP1_19_ proteins at 0.01, 0.1, and 1 μm concentrations (Figures 3 e and f). ELISA determination of tumor necrosis factor alpha (TNF‐α) and interleukin 12 (IL‐12) indicated cytokine production for cells stimulated with MSP1_19_‐biotin and MSP1_19_‐**GPI3**. The cytokine production was concentration‐dependent with highest cytokine secretion for MSP1_19_‐**GPI3** at 1 μm, and showed a tendency for higher TNF‐α and IL‐12 levels for the glypiated protein. These findings agree with previous reports describing this property for isolated GPIs from protozoa.7c, 38 Future studies will focus on the effect of glypiation for MSP1_19_ activity in vivo and its potential as a malaria vaccine candidate.

The difficulty in accessing GPI‐anchored proteins has limited investigations into the biological role of glypiation and its effect on the structure and activity of proteins. The herein reported OPL is an efficient method to glypiate the C‐terminus of proteins and provides access to naturally glypiated proteins with biological activity. This OPL method will help us to evaluate the effect of the GPI structure on protein folding, the behaviour of GPI‐APs in membranes, and the activity of glypiated proteins in vitro and in vivo.

## Conclusion

We have established a one‐pot ligation strategy for the glypiation of proteins using the DnaE split intein from *Nostoc punctiforme*. An active protein thioester is formed in situ and reacts with a synthetic cysteine‐containing GPI. The method tolerates folded and denaturated proteins, glycolipids with poor solubility in buffers, and additives for protein solubilization. We completed the semisynthesis of glypiated eGFP, Thy1, and *Plasmodium berghei ANKA* MSP1_19_ proteins and showed that the process does not affect protein folding. Structural analysis of the glypiated proteins by CD revealed no effect of glypiation on the overall structure of the eGFP and MSP1_19_ proteins in solution. Glypiation enhanced the proinflammatory activity of the MSP1_19_ protein in vitro, thus suggesting potential application of glypiated proteins in vaccine development.

## Conflict of interest

The authors declare no conflict of interest.

## Supporting information

As a service to our authors and readers, this journal provides supporting information supplied by the authors. Such materials are peer reviewed and may be re‐organized for online delivery, but are not copy‐edited or typeset. Technical support issues arising from supporting information (other than missing files) should be addressed to the authors.

Supplementary
