# Supplementary material for: Semisynthesis of Functional Glycosylphosphatidylinositol‐Anchored Proteins
Source: Angew Chem Int Ed Engl. 2020 May 18;59(29):12035–40. doi: 10.1002/anie.202002479 (PMC7383966; doi:10.1002/anie.202002479)
Supplement: Supplementary file 1 — Supplementary [file ANIE-59-12035-s001.pdf]

Angewandte Chemie
Eine Zeitschrift der Gesellschaft Deutscher Chemiker

Supporting Information

Semisynthesis of Functional Glycosylphosphatidylinositol-Anchored Proteins

*Renée F. Roller+, Ankita Malik+, Maria A. Carillo, Monika Garg, Antonella Rella, Marie-Kristin Raulf, Bernd Lepenies, Peter H. Seeberger, and Daniel Varón Silva**

anie_202002479_sm_miscellaneous_information.pdf

Supplementary Information

Synthesis of the Npu^{C} Peptide........2
Protein Expression and Purification........3
Protein Sequences........3
One-Pot Ligations with eGFP-Npu^{N}........11
OPL between eGFP-Npu^{N} and GPI1........13
OPL with Thy1-Npu^{N}........14
OPL with $MSP1_{19}$-Npu^{N}........16
Preparative OPL between $MSP1_{19}$-Npu^{N} and GPI3.........18
Structural Analysis by Circular Dichroism........20
Dendritic cells stimulation assay........20
Synthesis and Characterization of GPIs........22
General Methods for Synthetic chemistry........22
LC-MS Characterization of the GPIs........36
NMR Spectra........40
References........55

Synthesis of the *Npu*C Peptide

*Npu*C AA – Sequence: IKIATRKYLGKQNVYDIGVERDHNFALKNGFIASAAFNA

The modified C-terminal intein *Npu*C AA was synthesized via solid phase peptide synthesis (SPPS) using a microwave peptide synthesizer (Liberty Blue, CEM, USA).[1] Rink amide TentaGel S RAM resin was swollen in dichloromethane (DCM) and transferred to the reaction vessel of the synthesizer. The synthesis was run on the synthesizer using 1 M ethyl cyano(hydroxyimino)acetate (Oxyma)[2] with 0.1 M *N*,*N*-diisopropylethylamine (DIPEA) in dimethylformamide (DMF) as activator base and 0.5 M *N*,*N'*-diisopropylcarbodiimide (DIC) in DMF as activator. The fluorenylmehtyloxycarbonyl (Fmoc) group was removed after each coupling step by treatment twice with 20% piperidine in DMF containing 1% formic acid.[3] The peptide was cleaved from the resin with trifluoroacetic acid (TFA) containing triisopropylsilyl (TIPS) in water (90:5:5) by shaking the reaction for 2 h at room temperature. The cleaved peptide was precipitated and washed with ice-cold ether three times. Peptides were analyzed by RP-HPLC on a C18 Hydrosphere column (3 x 50 mm, YMC, Japan) using a 30-minute gradient of 5 – 70% acetonitrile in water with 0.1% TFA. The products were analyzed by MALDI-MS using 2,3-dihydroxybenzoic acid (DHB) as matrix and a reflector method in positive ion mode. The peptides were purified using a Waters HPLC system with a Hydrosphere C_{18} semi-preparative column (10 x 150 mm, YMC, Japan). Fractions containing the product were collected and lyophilized to give 20 mg of the *Npu*C AA. The isolated product was analyzed by LC-MS and MALDI-TOF-MS.

RP-HPLC Analysis for Peptide Samples. Peptide samples were analyzed in RP-HPLC using a Hydrosphere C18 column (50 x 3 mm, YMC, Japan) in an 1100 system (Agilent, USA), using a gradient from 5 – 70% acetonitrile in water (0.1% TFA) in 30 min. Chromatograms were analyzed using ChemStation software.

a)

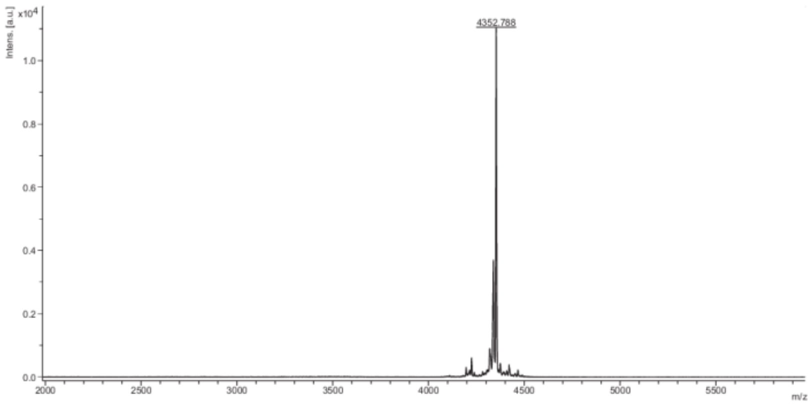

b)

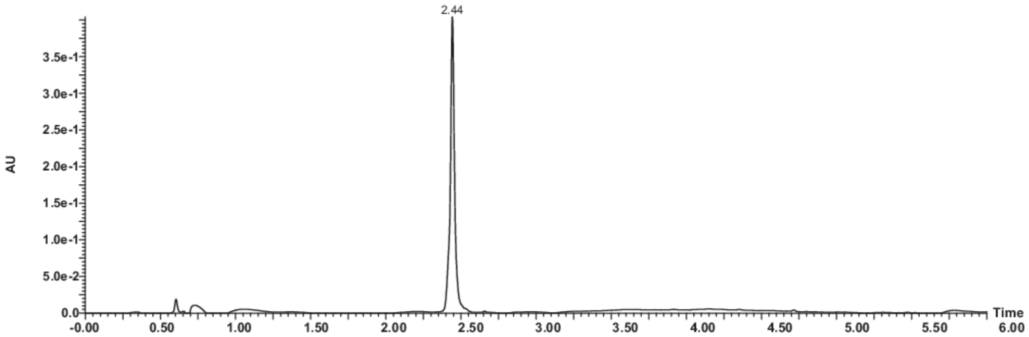

c)

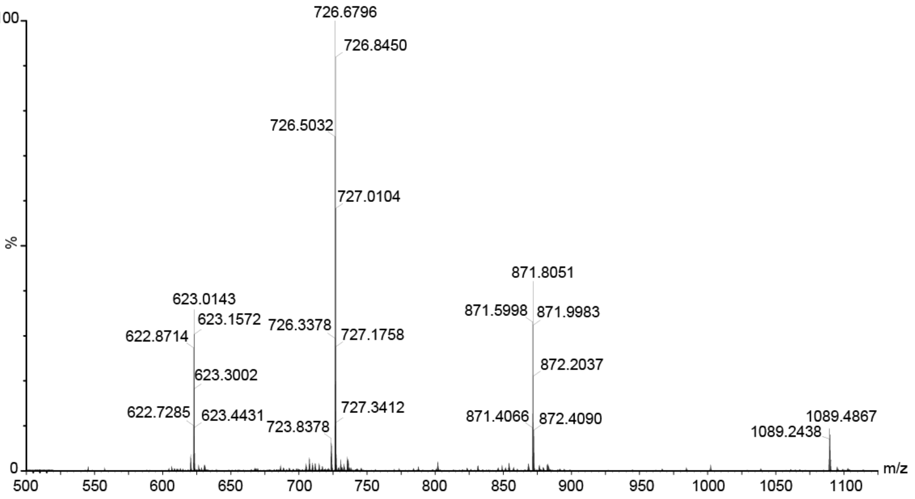

Figure **S1**. Characterization of *Npu*^{C}AA 16. a) MALDI-TOF spectrum of *Npu*^{C}AA , DHB was used as matrix, reflector positive mode $[M+H]^{+}_{Obs}$: 4352.788, $[M+H]^{+}_{Calc}$: 4353.32. b) Chromatogram (Waters BEH C18 1.7 µm, 50 x 2.1 mm, 5 – 70 % acetonitrile with 0.1%FA in water with 0.1% formic acid (FA), 0.3 mL/min), and c) LC-ESI-MS spectrum of purified *Npu*^{C}AA. $[M]^{+}_{calc}$=4352.32 Da, $[M]^{+}_{obs}$=4352.03 Da.

Protein Expression and Purification

Protein Sequences

eGFP-Npu^{N}

DNA sequence:

agttggagccacccgcagttcgaaaaagctagcggtaccgtgagcaagggcgaggagctgttcaccggggtggtgcccatcctg gtcgagctggacggcgacgtaaacggccacaagttcagcgtgtccggcgagggcgagggcgatgccacctacggcaagctgacc ctgaagttcatctgcaccaccggcaagctgcccgtgccctggcccaccctcgtgaccaccctgacctacggcgtgcagtgcttcagcc gctaccccgaccacatgaagcagcacgacttcttcaagtccgccatgcccgaaggctacgtccaggagcgcaccatcttcttcaagga cgacggcaactacaagacccgcgccgaggtgaagttcgagggcgacaccctggtgaaccgcatcgagctgaagggcatcgacttc aaggaggacggcaacatcctggggcacaagctggagtacaactacaacagccacaacgtctatatcatggccgacaagcagaagaa cggcatcaaggtgaacttcaagatccgccacaacatcgaggacggcagcgtgcagctcgccgaccactaccagcagaacacccccca tcggcgacggccccgtgctgctgcccgacaaccactacctgagcacccagtccgccctgagcaaagaccccaacgagaagcgcga tcacatggtcctgctggagttcgtgaccgccgccgggatcactctcggcatggacgagctgtacaagggatccagatctggatcctgtt taagctatgaaacggaaatattgacagtagaatatggattattaccgattggtaaaattgtagaaaagcgcatcgaatgtactgtttatagc gttgataataatggaaatatttatacacaacctgtagcacaatggcacgatcgcggagaacaagaggtgtttgagtattgtttggaagatg

gttcattgattcgggcaacaaaagaccataagtttatgactgttgatggtcaaatgttgccaattgatgaaatatttgaacgtgaattggatttgatgcgggttgataatttgccgaattaa

Amino acid sequence:

MASWSHPQFEKASGTVSKGEELFTGVVPILVELDGDVNGHKFSVSGEGEGDATYGKLTLKFICTTGKLPVPWPTLVTTLTYGVQCFSRYPDHMKQHDFFKSAMPEGYVQERTIFFKDDGNYKTRAEVKFEGDTLVNRIELKGIDFKEDGNILGHKLEYNYNSHNVYIMADKQKNGIKVNFKIRHNIEDGSVQLADHYQQNTPIGDGPVLLPDNHYLSTQSALSKDPNEKRDHMVLLEFVTAAGITLGMDELYKGSRSGSCLSYETEILTVEYGLLPIGKIVEKRIECTVYSVDNNGNIYTQPVAQWHDRGEQEVFEYCLEDGSLIRATKDHKFMTVDGQMLPIDEIFERELDLMRVDNLPN

MW: 40800.20 Da, theoretical pI: 5.10

Thy1-Npu^{N}

DNA sequence:

atgcagaaagtgaccagcctgaccgcgtgcctggtggatcagagcctgcgtctggattgccgtcatgaaaacaccagcagcagcccgattcagtatgaatttagcctgacccgtgaaaccaaaaaacatgtgctgtttggcaccgtgggcgtgccggaacatacctatcgtagccgtaccaactttaccagcaaatataacatgaaagtgctgtatctgagcgcgtttaccagcaaagatgaaggcacctatacctgcgcgctgcatcatagcggccatagcccgccgattagcagccagaacgtgaccgtgctgcgtgataaactggtgaaatgcctgagctacgagaccgaaattctgaccgtggagtatggtctgctgccgatcggcaagattgttgagaaacgtatcgaatgcaccgtgtacagcgttgacaacaacggcaacatttatacccagccggtggcgcaatggcacgatcgtggtgagcaggaagtttttgagtattgcctggaagacggcagcctgatccgtgcgaccaaggaccacaaattcatgaccgttgatggtcaaatgctgccgatcgacgagatttttgaacgtgaactggacctgatgcgtgttgacaatctgccgaaccaccaccaccaccaccactaa

Amino Acid sequence:

MQKVTSLTACLVDQSLRLDCRHENTSSSPIQYEFSLTRETKKHVLFGTVGVPEHTYRSRTNFTSKYNMKVLYLSAFTSKDEGTYTCALHHSGHSPPISSQNVTVLRDKLVKCLSYETEILTVEYGLLPIGKIVEKRIECTVYSVDNNGNIYTQPVAQWHDRGEQEVFEYCLEDGSLIRATKDHKFMTVDGQMLPIDEIFERELDLMRVDNLPNHHHHHH

MW: 25226.48 Da, theoretical pI: 6.14

MSP1$_{19}$-Npu^{N}

DNA sequence:

atgaacatggacagtatggacttgttgggcattgatccgaaacatgtttgcattaacactcgtgatatcccagccaatgcgggttgctttcgctacgataatggaaacgaagaatggcgctgtctcctgggctacaagaaaaacaataatacctgtattgaggactctaacccaacctgcggaaataataacggtggctgcgacccgacagcgggttgccagacggcggaaaaccgtgagaacagtaagaaaatcatctgcacttgcaaagaacccacgccaaacgcatactatgacggcgttttcggatcctgtttaagctatgaaacggaaatattgacagtagaatatggattattaccgattggtaaaattgtagaaaagcgcatcgaatgtactgtttatagcgttgataataatggaaatatttatacacaacctgtagcacaatggcacgatcgcggagaacaagaggtgtttgagtattgtttggaagatggttcattgattcgggcaacaaaagaccataagtttatga

ctgttgatggtcaaatgttgccaattgatgaaatatttgaacgtgaattggatttgatgcgggttgataatttgccgaatcatcatcatcatcatcattaa

Amino Acid sequence:

MNMDSMDLLGIDPKHVCINTRDIPANAGCFRYDNGNEEWRCLLGYKKNNNTCIED SNPTCGNNNGGCDPTAGCQTAENRENSKKIICTCKEPTPNAYYDGVFGCLSYETEILT VEYGLLPIGKIVEKRIECTVYSVDNNGNIYTQPVAQWHDRGEQEVFEYCLEDGSLIRA TKDHKFMTVDGQMLPIDEIFERELDLMRVDNLPNHHHHHH

MW: 24079.9 Da, Theoretical pI: 4.90

***Npu*^N^**

Amino Acid sequence:

CLSYETEILTVEYGLLPIGKIVEKRIECTVYSVDNNGNIYTQPVAQWHDRGEQEVFE YCLEDGSLIRATK DHKFMTVDGQ MLPIDEIFER ELDLMRVDNL PNHHHHHH

MW: 12671.15 Da, Theoretical pI: 4.97,

Expression and characterization of eGFP-Npu^N

Plasmid pVS017 coding for the eGFP-Npu^{N} fusion protein was kindly provided by Prof. Henning Mootz (University of Münster).[4] It was transformed into *E. coli* BL21 (DE3) Star cells and the fusion protein was expressed in 2 L LB medium with 100 µg/mL kanamycin with overnight induction (0.4 mM IPTG) at 18°C. The cells were harvested by centrifugation (4.000 x g, 10 min, 4°C). Pellets were resuspended in 10 mL buffer W (50 mM Tris/HCl, pH 7.5, 150 mM NaCl) + 1 mM PMSF and 0.5 mg/mL lysozyme and incubated 1 h at 30°C. For a more thorough lysis, the viscous suspension was then sonicated (10 min with pulses/pauses auf 30 sec on ice, 20% amplitude). The lysate was cleared by centrifugation (24.000 x g, 1 h, 4°C) followed by filtration through a 0.2 µm syringe filter and loaded very slowly onto StrepTactin columns equilibrated with buffer W (pH 7.5, CV = 2 mL). After washing with 5 x 2 CV buffer W the fusion protein was eluted with 6 x 0.5 CV buffer E (buffer W + 2.5 mM desthiobiotin). Fractions were analyzed using SDS-PAGE; fractions 2 – 5 contained the fusion protein and were combined for further purification on SEC (Superdex 75 pg HiLoad 16/600 on Knauer Azura FPLC, SEC buffer 50 mM Tris/HCl pH 7.5, 700 mM NaCl, flow rate 0.3 mL/min). Fractions containing the fusion protein (analyzed by SDS-PAGE) were combined and concentrated using centricons. 30 mg of pure fusion protein were obtained from 2 L culture volume. It was dialyzed in ligation buffer and stored at max. 2.5 mg/mL at 4°C in order to prevent dimerization. The expressed protein (eGFP-Npu^N) was characterized using RP-HPLC MALDI-TOF-MS (DHB as matrix substance, linear positive mode) as well as LC-ESI-MS (Figue S2).

RP-HPLC Analysis of Protein Samples. If not stated otherwise, all protein samples were analyzed in RP-HPLC using a Jupiter C_4 column (50 x 2.1 mm, Phenomenex, USA) in an Agilent 1100 system, applying a gradient from 10 – 70% acetonitrile in water with 0.1% TFA in 30 min. Chromatograms were analyzed using ChemStation software.

LC-MS Analysis. LC-MS analyses were performed using an Xevo G2-XS ESI-MS (Waters, USA) coupled to an Acquity H-class UPLC (Waters, USA). LC separations were done on an Acquity UPLC Protein BEH C_4 column (21 x 50 mm, 1,7 mL, Waters, USA), a Jupiter C_4 column (50 x 2.1 mm, Phenomenex, USA) or an Acquity UPLC BEH C_{18} column (Waters, USA), using a gradient of acetonitrile in water with 0.5% formic acid. The MS analysis was performed in positive or negative mode depending on the composition of the molecules.

a)

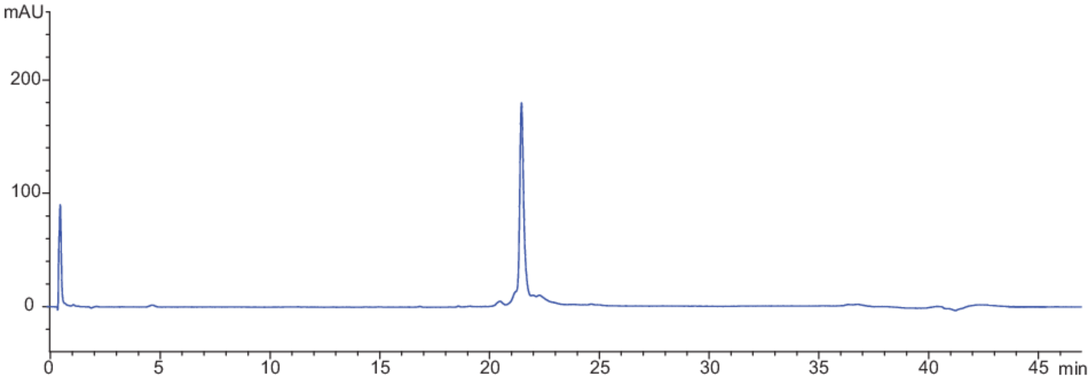

b)

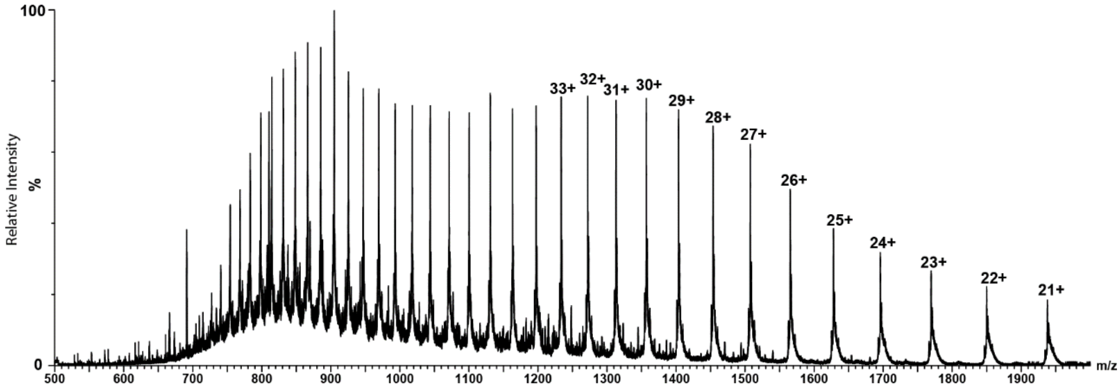

c)

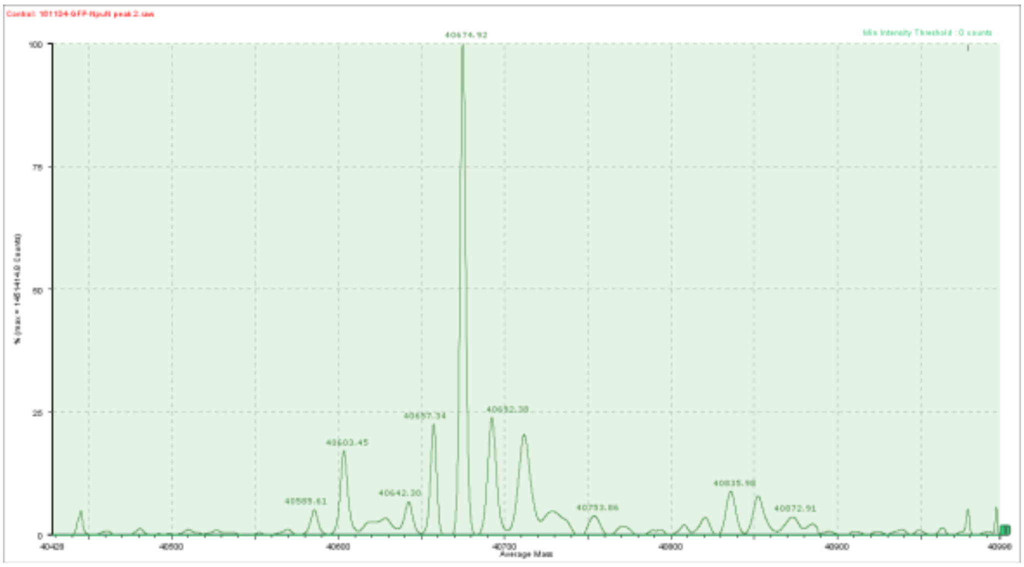

Figure S2. Characterization of eGFP-*Npu*[N] fusion protein. a) RP-HPLC, b) LC-ESI-MS, raw and c) deconvoluted spectra are shown, $[M+H]^+_{calc}$=40669.16 Da, $[M+H]^+_{obs}$=40674.92 Da.

Expression and characterization of Thy1-*Npu*[N]

Plasmid pET30a-Thy1-NpuN was ordered from GenScript (Piscataway, USA) and transformed into *E. coli* BL21 Rosetta cells using heat shock transformation and chemically competent cells. 1 L LB medium were inoculated to OD_{600} = 0.05, grown to late exponential phase (OD_{600} of 0.6 – 0.8) at 37°C, 220 rpm and protein expression was induced by addition of 0.5 mM IPTG. The temperature was changed to 30°C and shaking speed was reduced to 180 rpm. After 24 h induction, the cells were harvested by centrifugation (4.000 x g, 10 min, 4°C), the pellet was resuspended in 25 mL lysis buffer (20 mM Tris pH 8.0, 500 mM NaCl, 10 mM Imidazole) + 0.25 mg/mL lysozyme + protease inhibitors and incubated for 1 h at room temperature, 120 rpm. The suspension was subsequently sonicated for 20 min (pulses of 30 sec, 20% amplitude, on ice) and centrifuged (12.000 x g, 1 h, 4°C). The fusion protein was expressed in insoluble form, the lysis was repeated to remove all soluble proteins. The supernatant was discarded and the inclusion body pellet was solubilized in 20 mL HisTrap equilibration buffer + 8 M urea by vortexing, ultrasonic bath incubation for 2 h at room temperature (120 rpm). The solubilized inclusion bodies were centrifuged for 1.5 h (24.400 x g, 4°C) and filtered through a 0.2 µm syringe filter prior HisTag purification under denaturing conditions (8 M urea). For this, 8 mL lysate per run were loaded onto a HisTrap FF (5 mL) column equilibrated in HisTrap equilibration buffer + 8 M urea (on Knauer Azura FPLC, flow rate 1 mL/min). The column was washed with equilibration buffer + 8 M urea for 10 CV following elution of unbound host proteins, and the fusion protein was eluted using a gradient from 30 mM to 1 M imidazole over 5 CV. The fractions were analyzed in SDS-PAGE and fractions containing the fusion protein were combined and dialyzed into ligation buffer + 6 M urea. 40 mg fusion protein was obtained from 1 L culture volume. The purification process is shown in Figure S3.

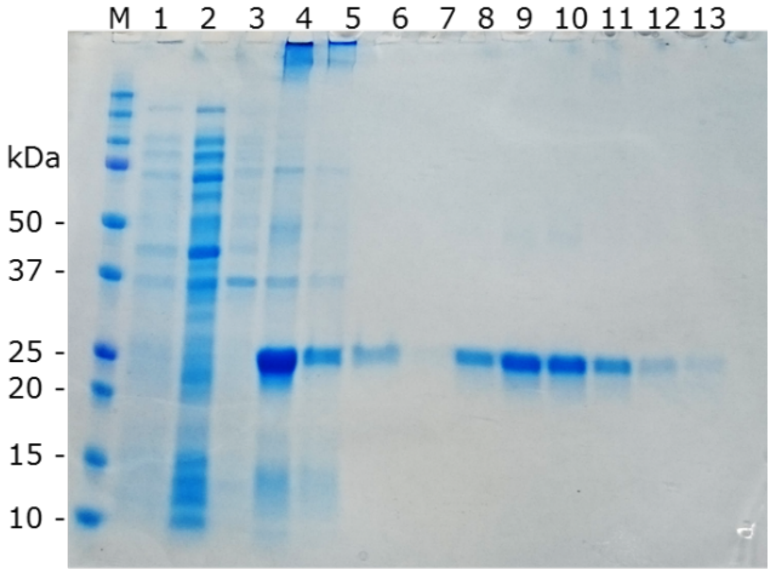

Figure **S3**. SDS-PAGE analysis of the purification of Thy1-*Npu*^{N} via HisTag. M: Molecular weight marker, 1: pre induction, 2: lysate 1, 3: lysate 2, 4: solubilized inclusion bodies (load) 5: flow-through HisTrap column, 6: wash fraction, 7 -13: elution fractions.

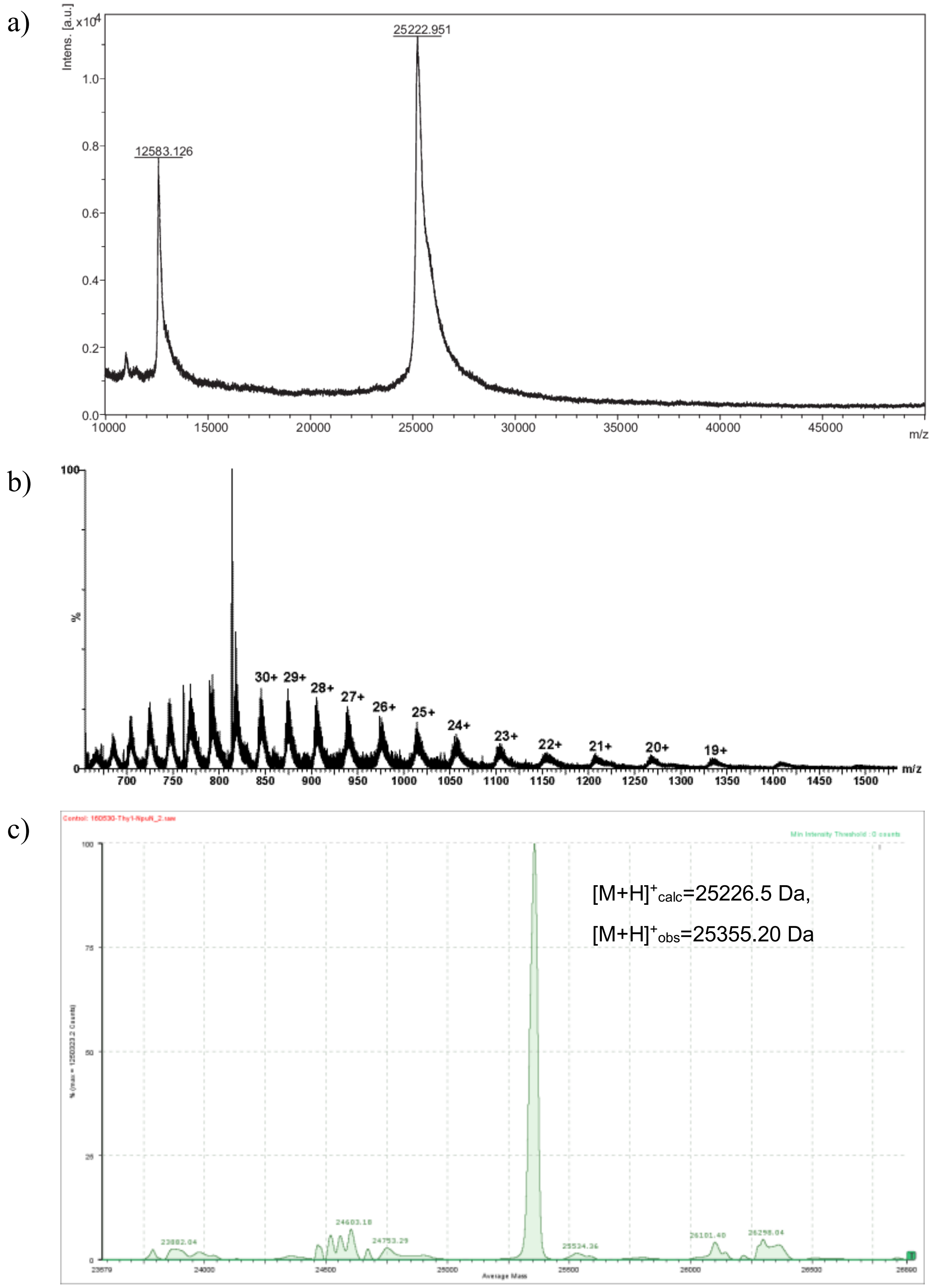

Figure **S4**. Characterization of Thy1-*Npu*^{N} fusion protein by MS. a) MALDI-TOF-MS spectrum $[M+H]^{+}_{calc}$=25226.5 Da, $[M+H]^{+}_{obs}$=25222.9 m/z. b) Raw and c) deconvoluted spectra from LC-ESI-MS analysis. Waters BEH C4 UPLC column (50 x 2.1 mm, 1.7 µm), gradient 10 – 70% ACN with 0.1% FA in water with 0.1% FA in 30 min, flow rate 0.5 mL/min, ES^+ mode. $[M+H]^{+}_{calc}$=25226.5 Da, $[M+H]^{+}_{obs}$=25356.21 m/z.

Expression and Characterization of MSP1$_{19}$-*Npu*^{N}

Plasmid pET29a+-MSP1$_{19}$-*Npu*^{N} was ordered from GenScript (Piscataway, USA) and transformed into *E. coli* BL21 Star (DE3) using heat shock transformation and chemically competent cells. 1 L LB medium were inoculated to OD_{600} = 0.05, grown to late exponential phase (OD_{600} of 0.6 – 0.8) at 37°C, 220 rpm and protein expression was induced with 0.5 mM IPTG and the temperature was changed to 25°C m. After 7 h induction, the cells were harvested by centrifugation (4.000 x g, 10 min, 4°C), the pellet was resuspended in 25 mL lysis buffer (20 mM Tris pH 8.0, 500 mM NaCl, 10 mM Imidazole) + 0.25 mg/mL lysozyme + protease inhibitors and incubated for 1 h at room temperature. The suspension was subsequently sonicated for 30 min using pulses of 60 sec. and 30 sec. pause, at 37°C, and centrifuged (12.000 x g, 2 h, 4°C).

The supernatant was loaded onto a HisTrap FF (5 mL) column equilibrated with 20 mM Tris buffer (pH 8.0, 500 mM NaCl) buffer. The column was washed with 10 CV of the buffer to remove unbound proteins. The fusion protein MSP1$_{19}$-*Npu*^{N} was eluted using a gradient from 30 mM to 1 M imidazole in 50 mM Tris buffer (pH 8.0, 150 mM NaCl) over 10 CV. The fractions were analyzed in SDS-PAGE and fractions containing the fusion protein were combined and dialyzed against 50 mM Tris buffer (pH 8.0, 10 mM NaCl). To separate proteins with higher molecular weight, the collected fractions from the HisTrap were eluted on a Superdex 75 column to obtain 6-10 mg of the MSP1$_{19}$-*Npu*^{N} protein. Purification of MSP1$_{19}$-*Npu*^{N} is shown in Figure S5.

a)

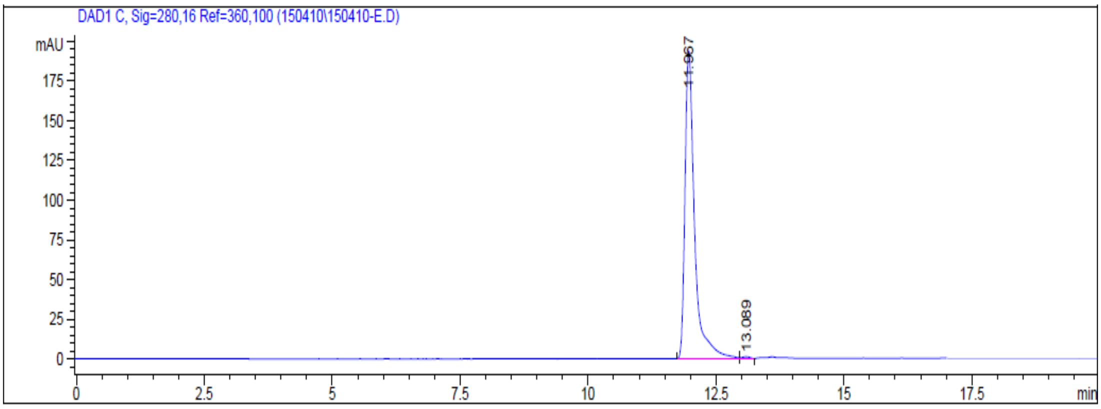

b)

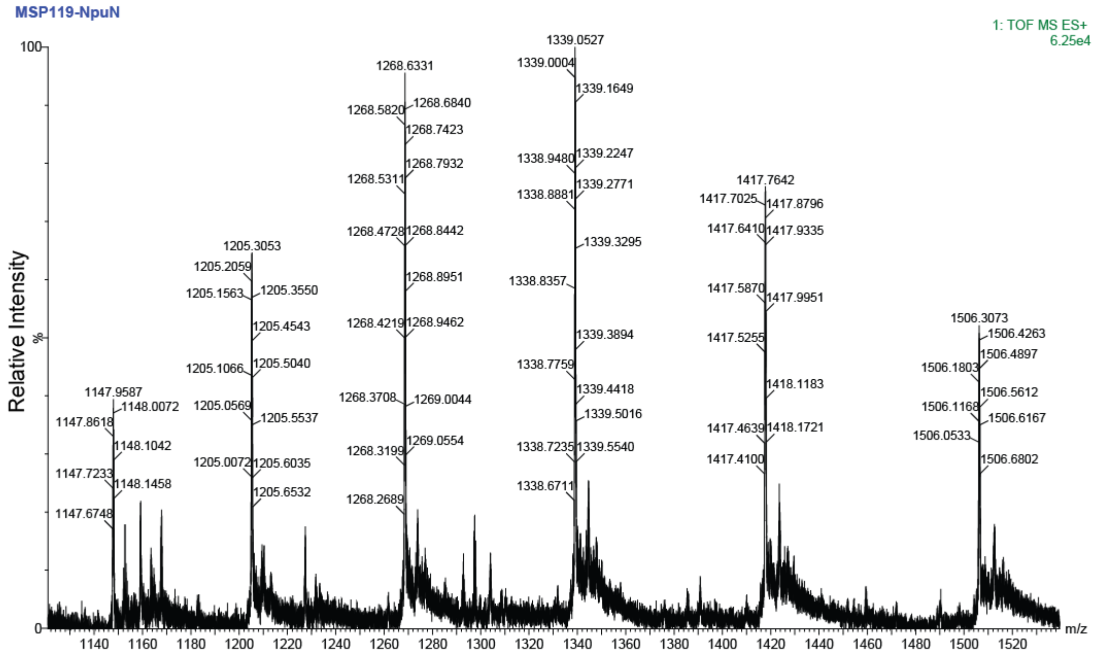

c)

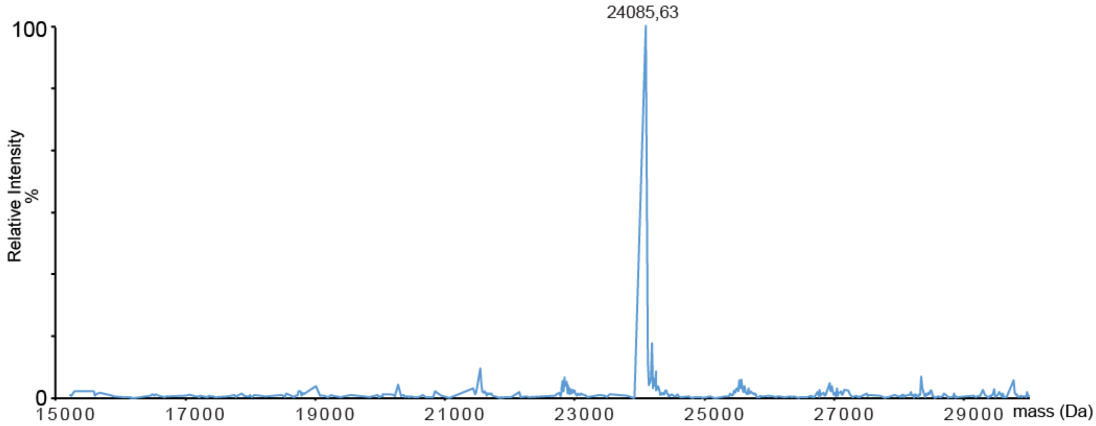

Figure **S5**. Characterization of the MSP1$_{19}$-*Npu*^{N} protein. a) Analytical chromatogram on a C4 Jupiter column, gradient 10-70% acetronitrile (0.1% formic acid) in water (0.1% formic acid). b) ESI-MS spectrum and c) deconvoluted ESI-MS spectrum of MSP1$_{19}$-*Npu*^{N}: MS $(M+H)^{+}$calc: 24079.19 Da, (M+H)+found: 24085.638 Da

One-Pot Ligations with eGFP-*Npu*[N]

a)

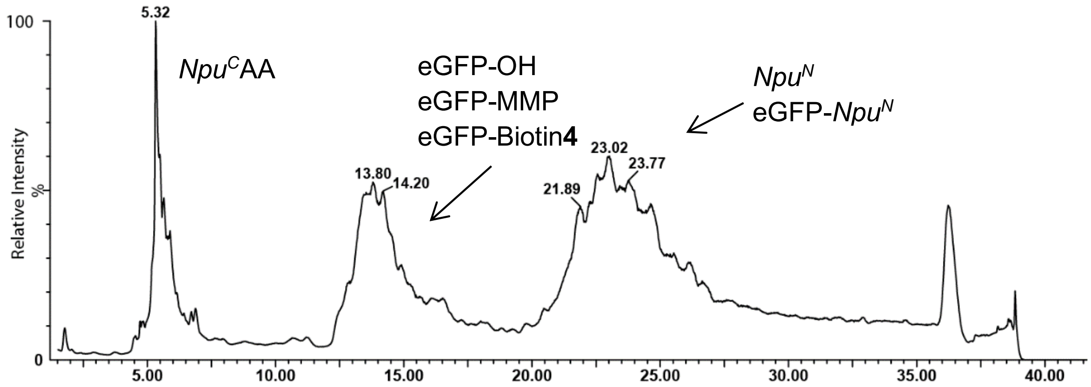

b)

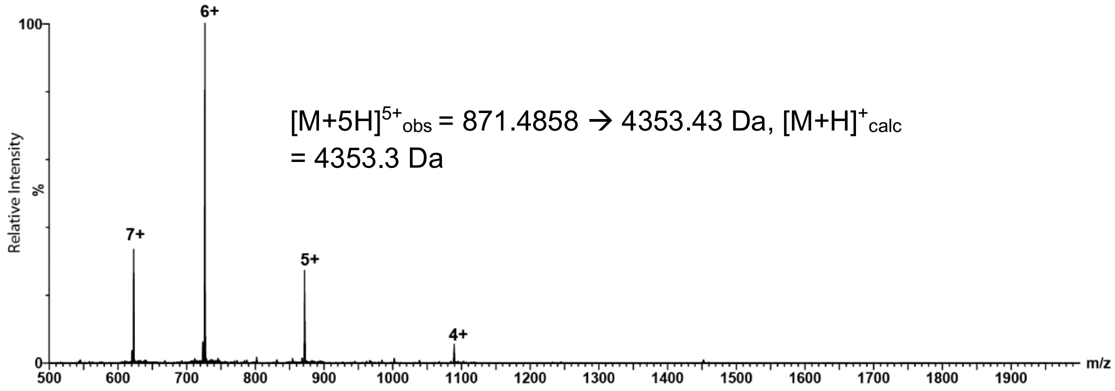

c)

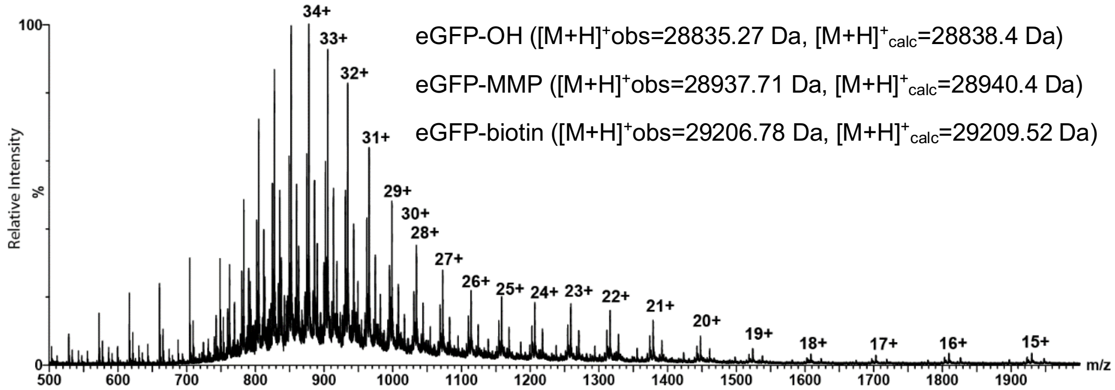

d)

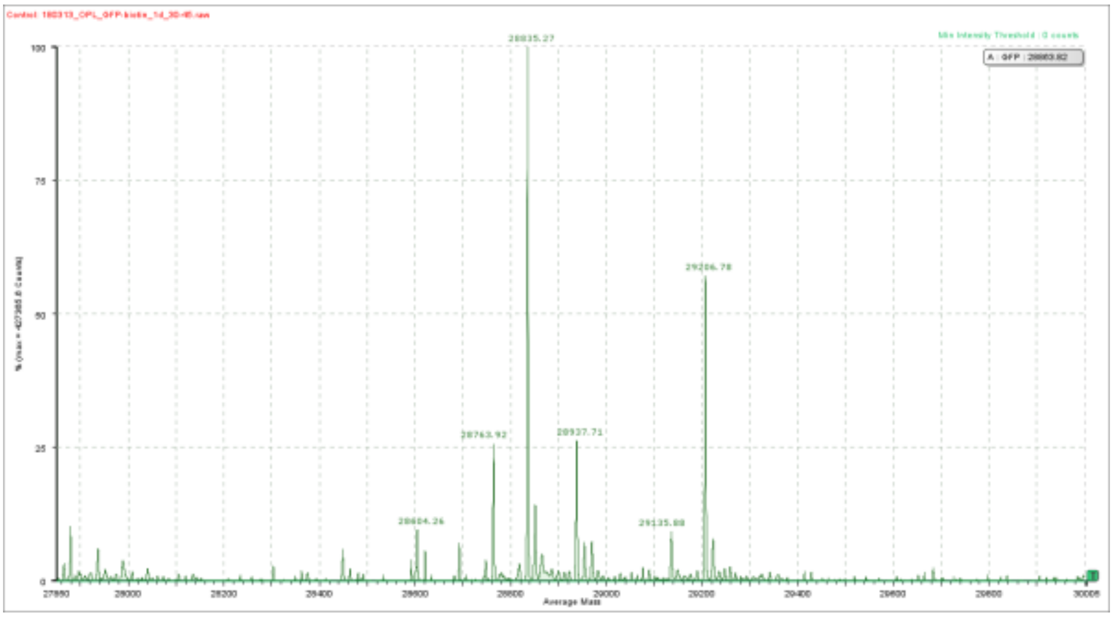

e)

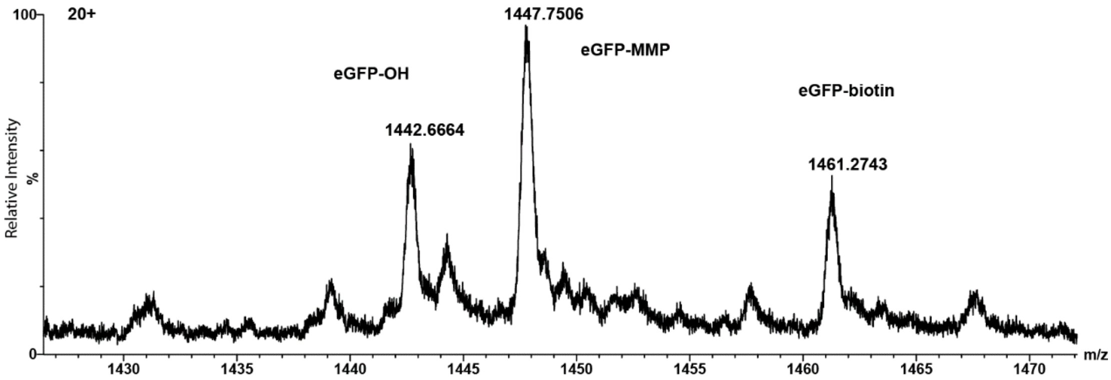

Figure **S6**. LC-ESI-MS analysis of OPL reaction between eGFP-*Npu*^{N} and Cys-biotin **4**. a) TIC profile of the OPL reaction between eGFP-*Npu*^{N} and Cys-biotin **4**, b) mass spectrum acquired at t_R=5.3 min *Npu*^{C}AA, MS observed 871.4858 Da $[M+5H]^{5+}$ corresponding to 4353.20 Da MS_{calc} = 4353.3 Da, c) Raw and d) deconvoluted spectrum acquired at t_R=13.8 min. It contains eGFP-OH, eGFP-MMP and eGFP-biotin. e) MS spectrum (zoom) showing the MS-signals of the products from c).

OPL between eGFP-Npu^N and GPI1

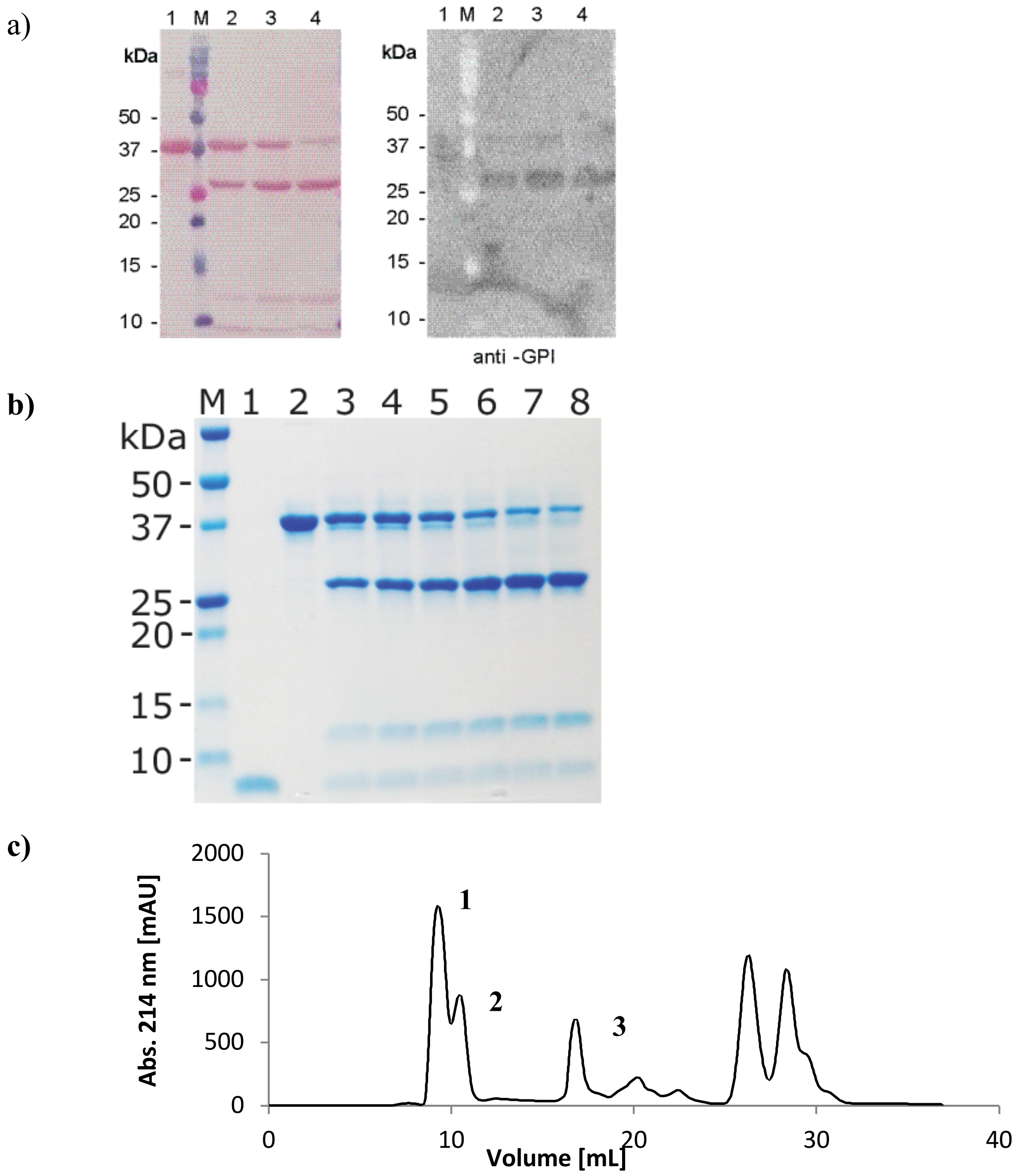

Figure **S7**. Analysis of OPL reaction between eGFP-Npu^N and **GPI1**, a) SDS-PAGE (left) and western blot (right, with anti-GPI antibody) of OPL between eGFP and **GPI1**, M – Marker, 1: eGFP-Npu^N, 2: OPL eGFP-**GPI1** day, 3 – OPL eGFP-**GPI1** 2 days, 4: OPL eGFP-**GPI1** 6 days. b) SDS-PAGE of the reaction; M: Marker, 1: NpuCAA, 2: eGFP-Npu^N, 3-8: reaction after 1, 2, 6, 24, 48 and 72 h; b) SEC chromatogram of purification of eGFP-**GPI1** from OPL. Peak 1: eGFP-**GPI1**, peak 2 eGFP-**GPI1** + eGFP-OH, peak 3: Npu^N.

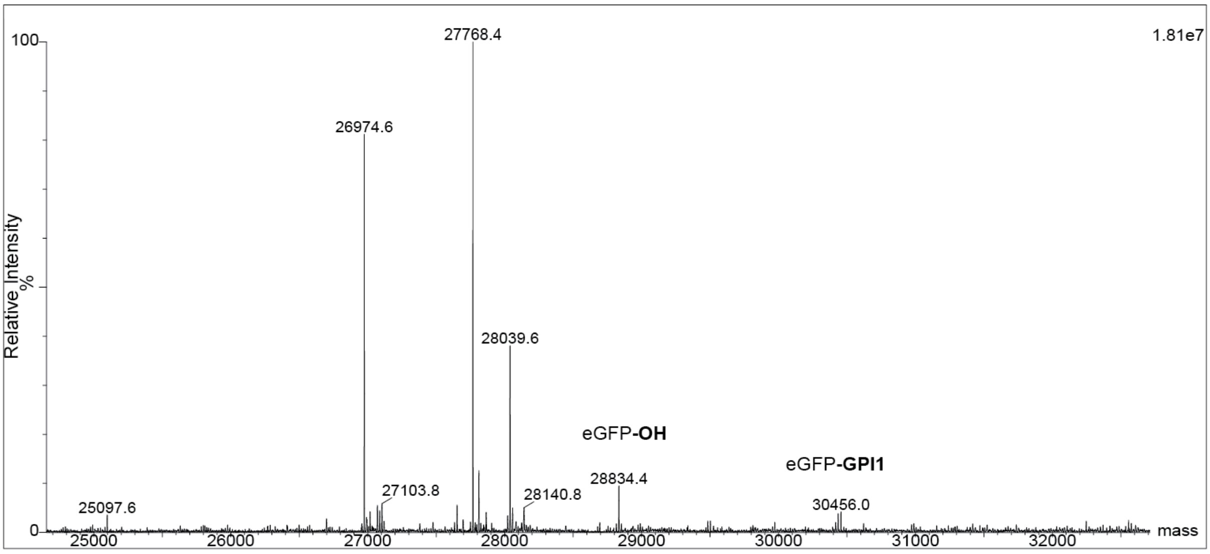

Figure **S8**. LC-ESI-MS analysis of eGFP-**GPI1**. $[M+H]^{+}_{calc}$=30205.9 Da, $[M+H]^{+}_{obs}$=30456.0 Da. eGFP-OH was detected as well.

OPL with Thy1-*Npu*^{N}

Optimization of OPL between Thy1-Npu^{N} and Cys-Biotin **4**.

To a solution of 15 µM fusion protein Thy1-*Npu*^{N} (1 mg) and 1.2 equivalents of *Npu*^{C}AA peptide in ligation buffer (20 mM Tris/HCl pH 7.0, 150 mM NaCl) containing 6 M urea, TCEP was added to a final concentration of 1, 5 and 10 mM. The cysteine Biotin **4** (10 equivalents) and the thiol reagent (50 mM of MMP) were added to the mixture. The ligation was incubated at 37 °C during six days. The reaction mixture was passed through a HisTrap column to separate the unreacted fusion protein and the cleaved *Npu*^{N} fragment having a HisTag. The reaction progress and the isolation of the product was monitored using RP-HPLC, SDS-PAGE and western blotting, Figure S9.

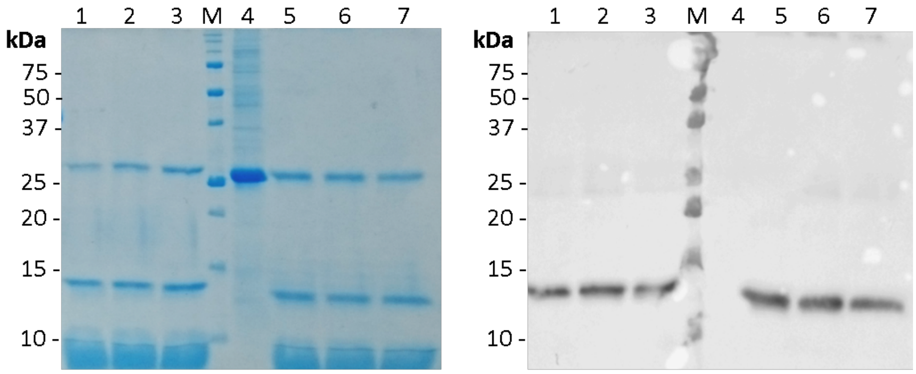

Figure **S9**. OPL between Thy1-*Npu*^{N} and Cys Biotin. A) SDS-PAGE of the reaction, b) Western blot with anti-biotin antibody-HRP. M: Molecular weight marker, 1: OPL r.t., 10 mM TCEP, 2: OPL r.t., 5 mM TCEP, 3: OPL r.t., 1 mM TCEP, 4: Thy1-*Npu*^{N}, 5: OPL 37°C, 1 mM TCEP, 6: OPL 37°C, 5 mM TCEP, 7: OPL 37°C, 10 mM TCEP.

OPL between GPI1 and Thy1-*Npu*^{N}.

The OPL was completed under the same conditions as described for cysteine biotin. Only 1 equivalent of the thiol reagent, (50 µg, 0.035 µmol) GPI1, was added to the reaction.

a)

b)

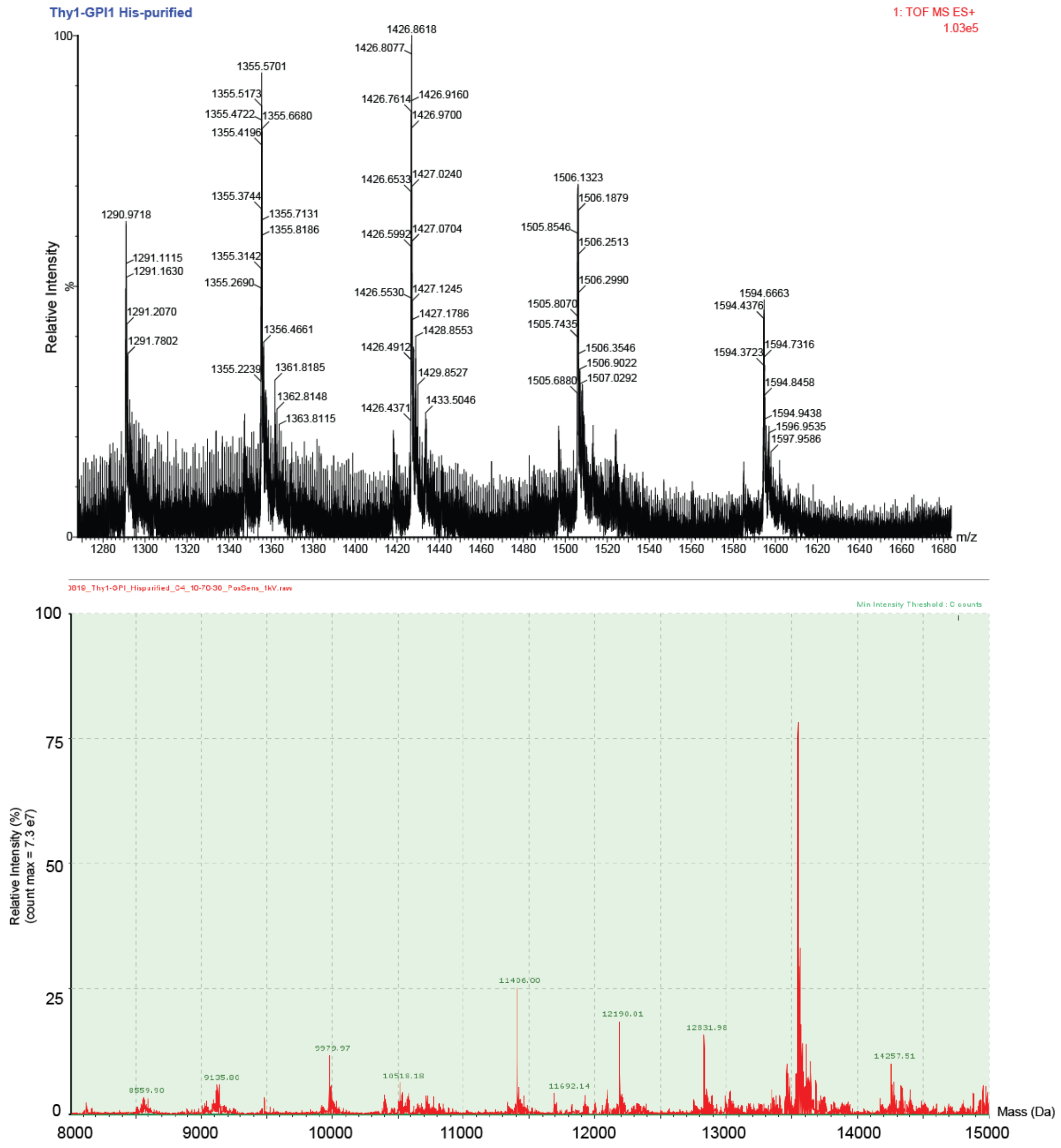

Figure **S10**. MS characterization of Thy1-**GPI1** after purification. a) ESI-TOF-MS Raw spectrum of the fraction containing the Thy1-**GPI1** product. b) Deconvoluted spectrum of the purified product. LC-ESI-MS analysis on a Waters BEH C4 column (50 x 2.1 mm, 1.7 µm), gradient 10 – 70% ACN with 0.1% FA in water with 0.1% FA in 15 min, flow rate 0.5 mL/min, ES^{+} mode with 1.5 kV capillary voltage. $[M+H]^{+}_{calc}$=13939.88 Da, see table for a summary of MS values the deconvoluted signals.

Table **S1**. Masses detected by LC-MS for Thy-**GPI1**

Entry	$[M+H]^+_{calc.}$	$[M+X]^+_{obs.}$	Species
1	12726.4	12,755.43	Thy1-PEtN
2	13589.03	13544.68	(Thy1-GPI**1**) -PI
3	13939.88	13998.1	(Thy1-GPI**1)**

OPL with MSP1$_{19}$-*Npu*^{N}

*Optimization of the OPL between MSP1$_{19}$-Npu^N and Cys-Biotin **4**.*

To a solution of 13 µM fusion protein MSP1$_{19}$-*Npu*^{N} (0.1 mg) and 1.5 equivalents of *Npu*^{C}AA peptide in ligation buffer (20 mM Tris/HCl pH 7.0, 150 mM NaCl), TCEP was added to a final concentration of 0.5 mM. The cysteine-Biotin **4** (4 equivalents) and the thiol reagent (50 mM of MMBA) were added to the mixture. The ligation was incubated at 37 °C and samples were taken after 1, 14 and 24 hours. The reaction progress was monitored using SDS-PAGE and western blot.

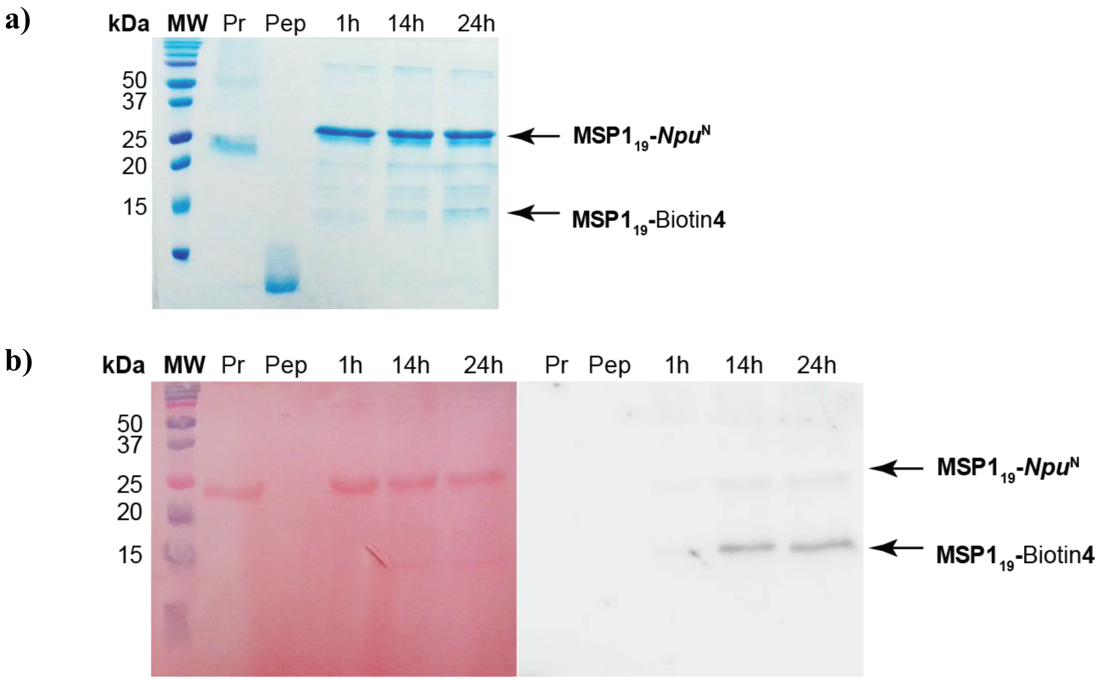

Figure **S11**. Analysis of the OPL reaction between MSP1$_{19}$-*Npu*^{N} and cysteine-biotin **4.** a) SDS-PAGE, and b) Western Blot analysis (right) using an anti-biotin antibody for detection.

OPL reaction between MSP1$_{19}$-Npu^N and GPIs.

To a solution of 13 µM fusion protein MSP1$_{19}$-*Npu*^{N} (0.1 mg) and 1.5 equivalents of *Npu*^{C}AA peptide in ligation buffer (20 mM Tris/HCl pH 7.0, 150 mM NaCl), TCEP was added to a final concentration of 0.5 mM. The cysteine containing GPI**1** and GPI**3** (4 equivalents) and the thiol reagent (50 mM of MMBA) were added to the mixture. The ligation was incubated at 37 °C

and samples were taken after 1, 24 and 72 hours. The reaction progress was monitored using SDS-PAGE and western blot. The reaction with GPI**3** was performed with and without of octyl glucoside (20 mM) as detergent.

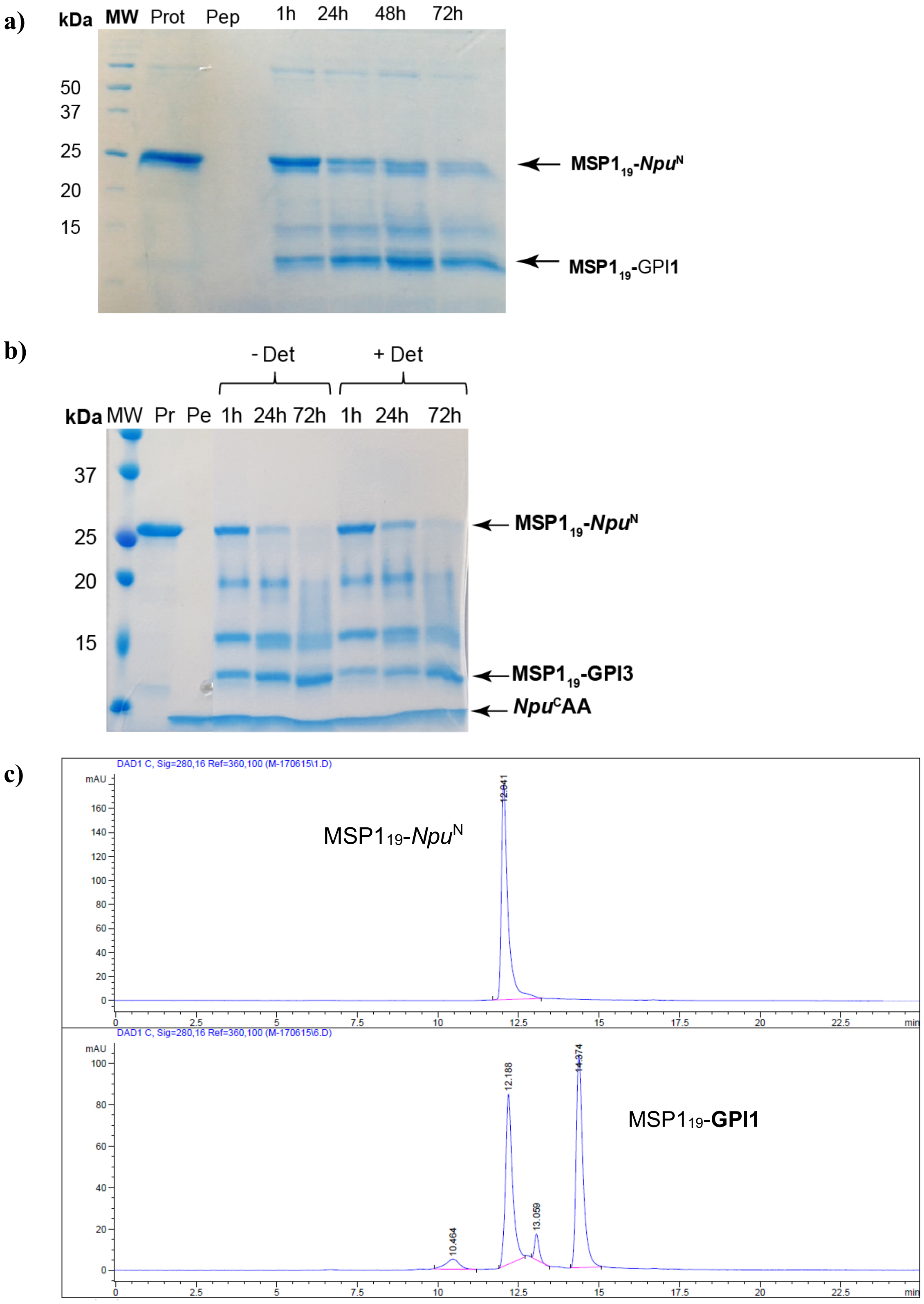

Figure S**12**. Analysis of the OPL reaction between MSP1$_{19}$-*Npu*^{N} and GPIs. a) SDS-PAGE of the OPL between MSP1$_{19}$-*Npu*^{N} and **GPI1**; b) SDS-PAGE of the OPL between

MSP1$_{19}$-*Npu*^{N} and **GPI3** with and without octyl glucoside; c) Analytical LC analysis of MSP1$_{19}$-**GPI1** using a Yarra 2000 SEC column.

a)

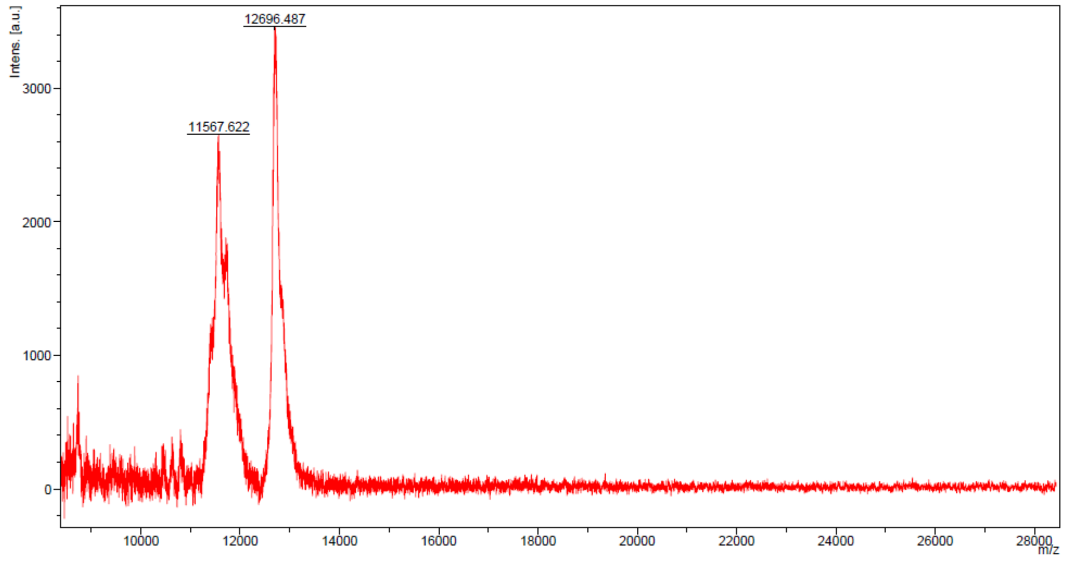

b)

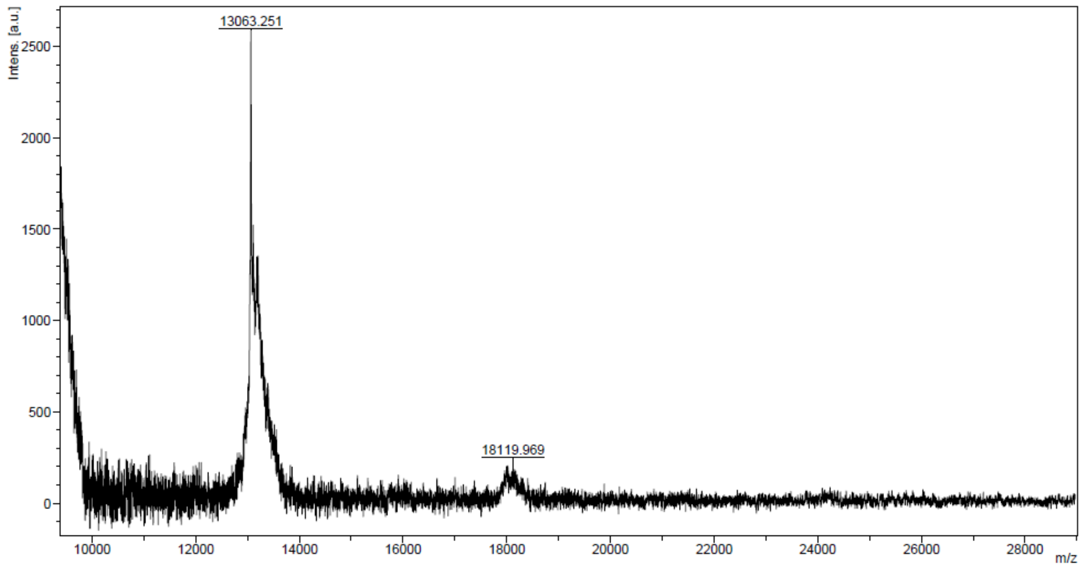

Figure **S13**. MALDI-TOF MS analysis of OPL with MSPI$_{19}$-*Npu*^{N} and **GPI3**. a) MALDI-MS spectrum of the isolated MSP1$_{19}$-SR and *Npu*^{N}, b) MALDI-MS spectrum of the product.

Preparative OPL between MSP1$_{19}$-*Npu*^{N} and GPI3.

GPI**3** (0.1 mg, 52.6 nmol) was suspended in 300 mL of in Tris buffer (20 mM Tris/HCl pH 7.0, 150 mM NaCl) and TCEP (0.12 mg, 0.41 µmol) were added. The mixture was stirred for 1 hour.

To a solution of 30 µM fusion protein MSP1$_{19}$-*Npu*^{N} (1 mg) in ligation buffer (20 mM Tris/HCl pH 7.0, 150 mM NaCl, 0.5 mM TCEP and 22 mM octyl glucoside) 1.5 equivalents of *Npu*^{C}AA peptide (0.27 mg) were added. The solution of the reduced **GPI3** and the thiol reagent (50 mM of MMBA) were added to the mixture and the volume was completed to 1.2 mL with

ligation buffer. The ligation was incubated at 37 °C for 4 days. The reaction was analyzed by LC-MS.

The reaction mixture was separated using a HisTrap column (1 mL). The unbound protein and peptide were collected and separated by SEC on a GE Healthcare Superdex 30 (16 x 600 mm) column. The fractions containing the product were concentrated to a final volume of 1 mL using a 5 mL centrifugation tube with a 5 KDa membrane filter to give 0.405 mg of the ligation product (70 % yield).

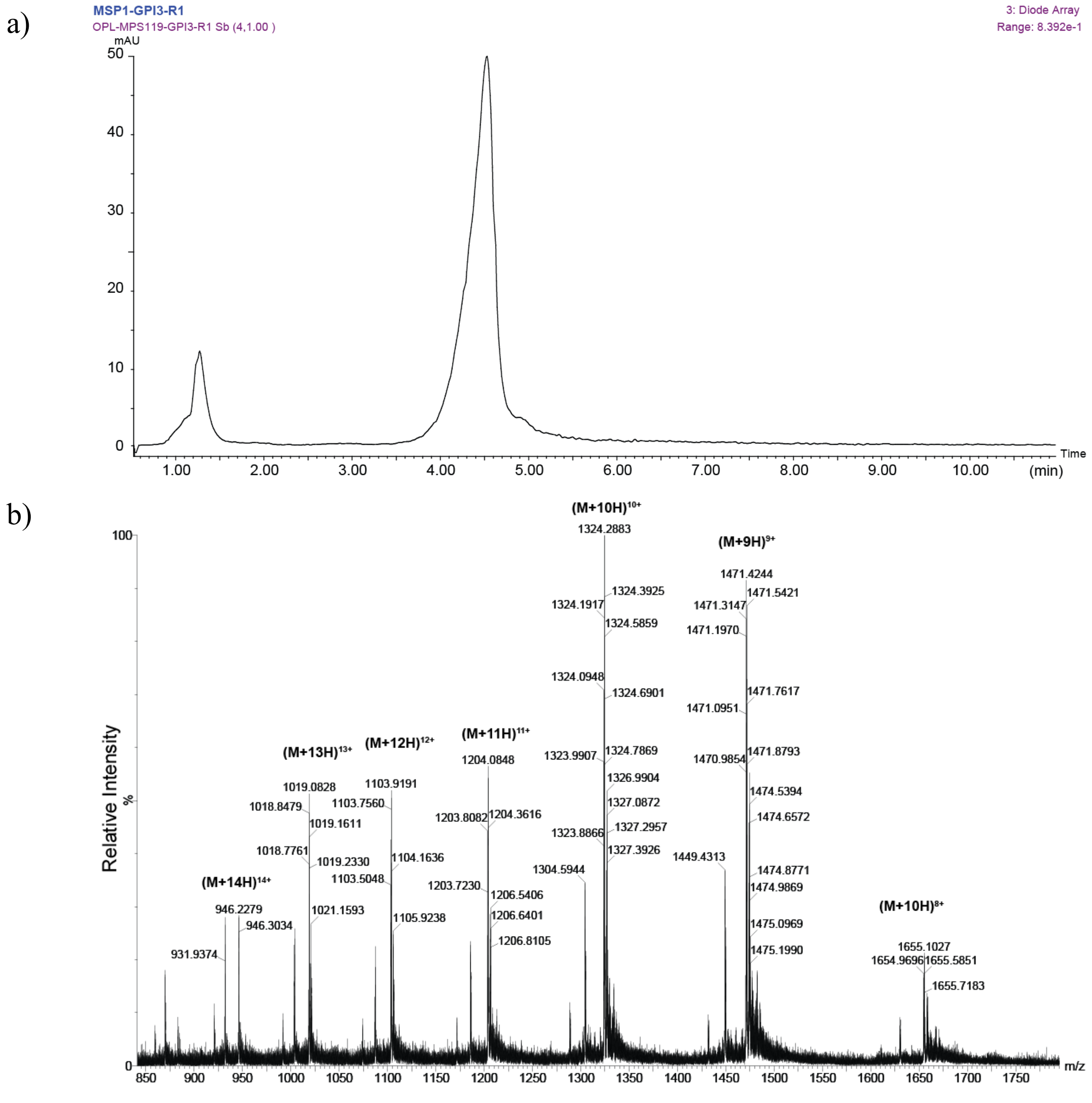

Figure **S14**. LC-Analysis of the purified MSP1$_{19}$-**GPI3**. a) Chromatogram at 214 nm of the LC separation on a Waters Acquity UPLC Protein BEH C4 column (2.1 x 50 mm, 300 Å, 1,7 µm), gradient 10-70% acetonitrile (0.5% formic acid) in water (0.5% formic acid) in 5 minutes; b) raw ESI-MS spectrum. MW calculated for MSP1$_{19}$-**GPI3**: 13222.8, M_{Found}: $(M+H)^+$: 13230.9 Da, $(M+H)^+$:13035.9 Da (product – 1 lipid chain).

Structural Analysis by Circular Dichroism

To evaluate the effect of the OPL reaction conditions and from the biotinylation and glypiation on the structures of the eGFP and $MSP1_{19}$ proteins, circular dichroism analysis was performed.

For CD measurements, protein samples were prepared in PBS buffer at concentrations ranging from 0.1 – 1 mg/mL, depending on the amounts available. The protein solution was then filled into a high precision cell quartz cuvette (light path 1 mm) and placed in the Chirascan Circular Dichroism Spectrometer. Circular dichroism was measured between 195 and 250 nm against PBS buffer as a reference and measurements were performed in triplicates. The averages of these measurements were plotted over the wavelength.

a) b)

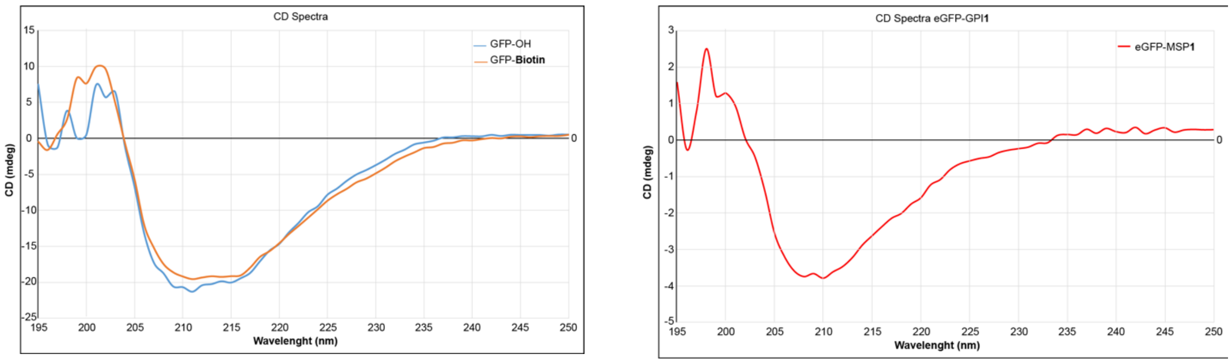

Figure **S15**. CD spectra of the Proteins after OPL. A) eGFP-biotin, eGFP-OH, b) eGFP-**GPI1**). The β-sheet structure of eGFP is not compromised by C-terminal modification with biotin and **GPI1**. The protein also retains its fluorescence.

Dendritic cells stimulation assay

Bone marrow cells were obtained from femur and tibia of C57BL/6 mice and were differentiated into BMDCs using differentiation medium (IMDM medium, 10% fetal bovine serum, 2 mM L-glutamine, 100 U/ml penicillin, 100 µg/ml streptomycin, 10% X63-GM-CSF supernatant) at 37°C and 5% CO_2. After 9 days of differentiation, BMDCs were seeded at a concentration of $5x10^5$ cells/ml in a 96-well plate and stimulated with the samples (see list below) at 37°C and 5% CO_2 for 16 to 20 h. On the next day, supernatants were harvested and cytokine concentrations were measured by ELISA (Murine TNF-α Standard ABTS ELISA Development Kit, Murine IL-12 Standard ABTS ELISA Development Kit, PeproTech, Hamburg, Germany).

- *Negative control:* 100µl medium
- *Positive control LPS:* 1µg/ml
- *MSP1-Biotin* 1µM: Stock in medium
- *MSP1-Biotin* 0.1µM: 1:10 out of 1µM

- *MSP1-Biotin* 0.01µM: 1:10 out of 0.1µM
- *MSP1-**GPI3*** 1µM: Stock in medium
- *MSP1-**GPI3*** 0.1µM: 1:10 out of 1µM
- *MSP1-**GPI3*** 0.01µM: 1:10 out of 0.1µM

Differentiation of BMDCs was checked by CD11c surface staining. BMDCs were blocked with anti-mouse CD16/32 (93, eBioscience, 1:100) for 10min at 4°C and stained with APC-conjugated anti-mouse CD11c (N418, eBioscience, 1:200). Samples were analyzed using an Attune NxT Flow Cytometer (Thermo Fisher Scientific).

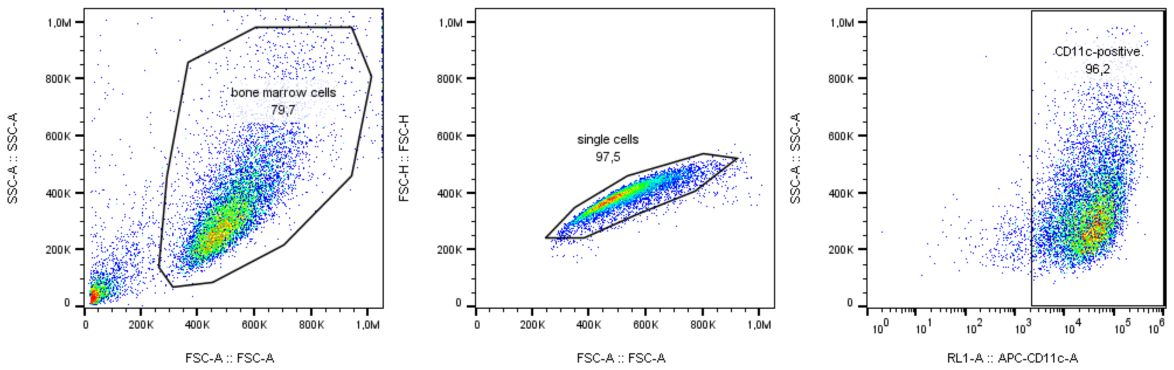

Figure **S16**. Gating strategy for BMDC differentiation. Cells were gated on size (FSC) and granularity (SSC) followed by gating on single cells (FSC-A against FSC-H). BMDC differentiation was evaluated based on CD11c expression, indicating a differentiation efficiency higher than 95.0%.

Target	Dye	Reactivity	Company	Reference	Lot	Dilution
CD11c	APC	mouse	N418, eBioscience	17-0114-82	4304965	1:200

Synthesis and Characterization of GPIs

General Methods for Synthetic chemistry

All purchased chemicals were of reagent grade and all anhydrous solvents were of high-purity grade and used as supplied except where noted otherwise. Reactions were performed in oven-dried glassware under an inert argon atmosphere unless noted otherwise. Reagent grade thiophene was dried over activated molecular sieves prior to use. Pyridine was distilled over CaH_2 prior to use. Sodium hydride suspension was washed with hexane and THF and stored in an anhydrous environment. Benzyl bromide was passed through activated basic aluminum oxide prior to use. Metal sodium was washed with hexane and stored in hexane. Analytical thin layer chromatography (TLC) was performed on Merck silica gel 60 F_{254} plates (0.25mm). Compounds were visualized by UV irradiation or heating the plate after dipping in staining solution. The staining solutions were cerium sulfate-ammonium molybdate (CAM) solution, basic potassium permanganate solution, acidic ninhydrin-acetone solution, or a 3-methoxyphenol-sulfuric acid solution (Sugar Stain). Flash column chromatography was carried out using a forced flow of the indicated solvent on Sigma Aldrich silica gel high purity grade 60 Å (230-400 mesh particle size, for preparative column chromatography). Solvents were removed under reduced pressure using rotary evaporator and high vacuum (<1 mbar). Freeze drying of the aqueous solutions was performed using Alpha 2-4 LD Lyophilizer (Christ, Osterode am Harz, Germany)

^{1}H, ^{13}C and ^{31}P-NMR as well as all 2D-spectra (^{1}H-^{1}H COSY, ^{1}H-^{1}H TOCSY, ^{1}H-^{13}C HSQC, ^{1}H-^{13}C HMBC) were recorded on a Varian 400 (400 MHz), a Varian 600 (600 MHz), a Bruker 400 (400 MHz) and a Bruker Ascend 400 (400 MHz) spectrometer in $CDCl_3$ (7.26 ppm ^{1}H, 77.1 ppm ^{13}C), D_2O (4.79 ppm ^{1}H), MeOD (4.87 ppm and 3.31 ppm ^{1}H, 49.00 ppm ^{13}C), Acetone-d_6 (2.05 ppm and 2.84 ppm ^{1}H, 206.26 ppm and 29.84 ppm ^{13}C) unless otherwise stated. The coupling constants (J) are reported in Hertz (Hz). Splitting patterns are indicated as s, singlet; d, doublet; t, triplet; q, quartet; br, broad singlet; dd, doublet of doublets; m, multiplet; dt, doublet of triplets; h, hextet for ^{1}H NMR data. Signals were assigned by means of ^{1}H-^{1}H COSY, ^{1}H-^{1}H TOCSY, ^{1}H-^{13}C HSQC, ^{1}H-^{13}C HMBC spectra and version thereof. ESI mass analyses were performed by Waters Xevo G2-XS Q-TOF with an Acquity H-class UPLC and a Bruker Autoflex-speed MALDI-TOF spectrometer.

*Allyl 2,3,4,6-tetra-O-benzyl-α-D-mannopyranosyl-(1→2)-3,4-di-O-benzyl-6-O-triisopropylsilyl-α-D-mannopyranosyl-(1→2)-3,4,6-tri-O-benzyl-α-D-mannopyranosyl-(1→6)-2-O-levulonoyl-3-benzyl-4-O-(2-naphthyl)methyl-α-D-mannopyranoside (*__14__*)*

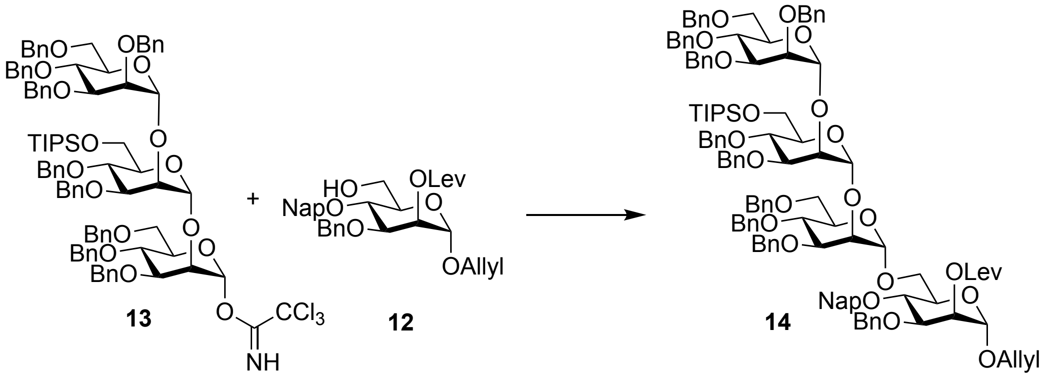

A mannose acceptor **12**[5] (0.509 g, 0.928 mmol) and trimannosyl donor **13**[6] (1.65 g, 1.021 mmol) were dissolved and co-evaporated with dry toluene three times and dried under high vacuum for 2 h. The reaction mixture was dissolved in dry thiophene/DCM (2:1, 18 mL) under argon atmosphere, followed by the addition of activated powdered molecular sieves. Then, 21.7 µL (0.093 mmol) of TBSOTf were added to the reaction mixture under argon atmosphere. After 2 h, the reaction was quenched by using Et_3N, filtered the molecular sieves and concentrated. The residue was purified using column chromatography to obtain tetrasaccharide **14** (1.24 g, 0.619 mmol, 66.7%, α-anomer). R*f* = 0.25 (n-Hex/AcOEt, 3:1) ^{1}H NMR (400 MHz, $CDCl_3$) δ 7.94 – 7.85 (m, 4H, Ar), 7.61 – 7.55 (m, 3H, Ar), 7.52 – 7.28 (m, 50H, Ar), 7.22 (t, *J* = 7.3 Hz, 1H, CH- allyl), 5.82 (dddd, *J* = 16.9, 10.9, 6.3, 4.9 Hz, 1H, -CH-CH_2 allyl), 5.42 (d, *J* = 1.6 Hz, 2H, CH_2- Bn), 5.33 (d, *J* = 1.7 Hz, 1H, anomeric), 5.25 (d, *J* =1.3 Hz, 1H, anomeric), 5.24 (dd, *J* = 10.3 1.6 Hz, 1H, =CH2 allyl), 5.16 (dd, *J* = 1.3, 11.2 Hz, 1H, =CH_2 Bn), 5.05 (d, *J* = 10.9 Hz, 1H, CH_2= allyl), 4.99 (d, *J* = 10.7 Hz, 2H, CH_2- Bn), 4.88 (d, *J* = 1.8 Hz, 1H, CH_2- Bn), 4.87 (d, *J* = 1.8 Hz, 1H, anomeric), 4.80 (d, *J* = 1.8 Hz, 1H, anomeric), 4.94 – 4.54 (m, 21H), 4.46 – 3.82 (m, 21H), 3.77 – 3.57 (m, 5H), 2.87 – 2.73 (m, 4H, 2xCH_2 Lev), 2.24 (s, 3H, CH_3 Lev), 1.22 (m, 21H, CH_3-CH TIPS). ^{13}C NMR (101 MHz, $CDCl_3$) δ: 206.3 (C=O ketone), 172.2 (C=O ester), 163.6, 138.8, 138.8, 138.7, 138.6, 138.5, 138.4, 138.3, 138.3, 138.2, 138.1, 137.9, 133.2 (CH=CH_2), 128.5, 128.4, 128.4, 128.3, 128.3, 128.2, 128.2, 128.2, 127.9, 127.9, 127.9, 127.9, 127.8, 127.8, 127.7, 127.6, 127.6, 127.5, 127.5, 127.5, 127.5, 127.5, 127.4, 127.4, 118.2 (=CH_2), 100.3 (C-anomeric), 99.1 (C-anomeric) 98.8 (C-anomeric), 96.4 (C-anomeric), 80.2, 78.5, 75.0, 74.9, 74.9, 74.8, 74.7, 74.6, 74.3, 73.3, 73.2, 73.0, 72.6, 72.2, 72.1, 72.0, 71.6, 71.6, 70.6, 68.8, 67.9, 37.9 (-CH_2- Lev), 29.8 (-CH_3 Lev), 28.1 (-CH_2- Lev), 18.2 (CH_3- TIPS), 12.0 (-CH- TIPS). ESI-MS (*m*/*z*): $[M+Na]^+$ calcd 1953.851, $[M+Na]^+$obs 1953.747.

2-3,4,6-Tetra-O-benzyl-α-D-mannopyranosyl-(1→2)-3,4-di-O-benzyl-6-O-triisopropyl silyl-α-D-mannopyranosyl-(1→2)-3,4,6-tri-O-benzyl-α-D-mannopyranosyl-(1→6)-2-O-levulonoyl-3-O-benzyl-4 O-(2-(naphthyl)methyl-α-D-mannopyranosyl trichloroacetamidate **(15)**

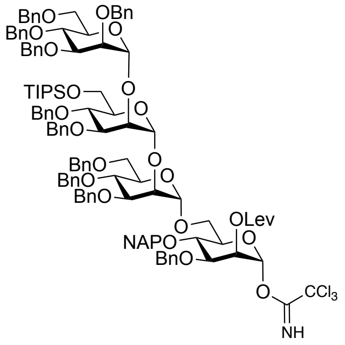

A solution of [IrCOD(PPh_2Me)$_2$]PF_6 (15.0 mg) in THF (3 mL) was stirred under hydrogen atmosphere until the color of the solution turned from red to colorless to pale yellow. The hydrogen atmosphere was exchanged with Argon. This solution was added to a solution of tetrasaccharide **142** (1.20 g, 0.599 mmol) in THF (6 mL). After 16 h, the solvent was removed in *vacuo* and the residue was dissolved in a mixture of acetone (11 mL) and water (1.2 mL). Mercury (II) chloride (0.813 g, 3 mmol) and mercury (II) oxide (19 mg, 0.090 mmol) were added. After 1 h, saturated $NaHCO_3$ (aq) was added and the reaction mixture was extracted three times with CH_2Cl_2. The combined organic layer was dried over Na_2SO_4, filtered and concentrated. The crude product was purified by silica gel column chromatography to give trisaccharide hemiacetal (1.01 g, 0.51 mmol, 86 %) as colorless oil. ^{1}H NMR (400 MHz, $CDCl_3$) δ: 7.76 (d, *J* = 8.2 Hz, 1H, Ar), 7.66 (d, *J* = 8.5 Hz, 1H, Ar), 7.63 (s, 2H, Ar), 7.43 – 7.39 (m, 1H), 7.32 – 7.07 (m, 67H, Ar), 7.04 – 6.99 (m, 1H, Ar), 6.08 (dd, *J* = 12.3, 1.8 Hz, 1H), 5.43 (d, *J* = 3.3 Hz, 1H), 5.29 (s, 1H, anomeric), 5.18 (s, 1H, anomeric), 5.09 (dd, *J* = 12.4, 6.8 Hz, 1H, C<u>H$_2$</u>- Bn), 5.03 (d, *J* = 11.5 Hz, 1H, C<u>H$_2$</u>- Bn), 4.97 (s, 1H), 4.92 (s, 1H), 4.87 (d, *J* = 3.8 Hz, 1H, anomeric), 4.84 (d, *J* = 3.9 Hz, 1H), 4.81 (d, *J* = 10.9 Hz, 1H), 4.71 – 4.66 (m, 2H), 4.64 (s, 1H), 4.62 – 4.40 (m, 16H), 4.29 (dd, *J* = 12.2, 5.0 Hz, 2H), 4.15 (d, *J* = 20.5 Hz, 3H), 4.06 – 3.68 (m, 11H), 3.62 – 3.41 (m, 4H, 2 x CH_2- Lev), 2.61 (s, 3H), 2.08 (d, 3H, CH_3- Lev), 1.87 – 1.83 (m, 3H), 1.01 (m, 21H, CH_3-, CH, TIPS). ESI-MS (*m*/*z*): $[M+Na]^+$ cald for $C_{129}H_{137}O_{23}Si$ 1983.924 $[M+Na]^+$ obsd 1983.837.

To a stirred solution of obtained hemiacetal (1.01 g, 0.51 mmol) in anhydrous CH_2Cl_2 (6 mL) were added trichloroacetonitrile (0.613 mL, 1.11 mmol) and DBU (10 μL, 0.061 mmol) at 0 °C. The reaction mixture was stirred and warmed up to room temperature. After 2 h, volatiles were evaporated and the crude reaction mixture was purified using silica gel column chromatography to get the imidate **15** (0.980 g, 0.455 mmol, 89%). *Rf* = 0.35 (n-Hex/AcOEt, 2:1) ^{1}H NMR (400 MHz, $CDCl_3$) δ: 7.75 (d, *J* = 7.7 Hz, 1H, <u>NH</u>), 7.68 – 7.60 (m, 3H, Ar), 7.46 – 7.06 (m, 79H, Ar), 7.04 – 6.99 (m, 1H, Ar), 6.08 (dd, *J* = 12.5, 1.9 Hz, 1H, <u>anomeric</u>), 5.42 (d, *J* = 3.2 Hz, 1H, <u>CH$_2$</u>- Bn), 5.29 (s, 1H, anomeric), 5.18 (s, 1H, anomeric), 5.09 (dd, *J* = 12.5, 6.7 Hz, 1H), 5.03 (d, *J* = 11.5 Hz, 1H), 4.98 – 4.90 (m, 2H), 4.88 – 4.78 (m, 4H), 4.72 – 4.40 (m, 19H), 4.29 (dd, *J* = 12.2, 4.9 Hz, 2H), 4.15 (d, *J* = 20.5 Hz, 2H), 4.06 – 3.68 (m, 27H), 3.62 – 3.41 (m, 5H), 2.61 (m, 4H, 2 x CH_2 Lev), 2.08 (s, 3H, CH_3 Lev), 1.85 (s, 1H), 1.02 (m, 21H, <u>CH_3-CH</u> TIPS). ESI-MS cald for $C_{121}H_{136}Cl_3NO_{23}Si$: 2103.834 MS obs: 2104.847 $[M+H]^+$, 2126.852 $[M+Na]^+$. ^{13}C NMR (101 MHz, $CDCl_3$) δ 205.8 (C=O ketone), 172.0 (C=O ester), 160.28 (imidate), 139.1, 139.0, 138.9, 138.6, 138.5, 138.5, 138.0, 128.8, 128.7, 128.6, 128.5, 128.5, 128.5, 128.5, 128.4, 128.4, 128.3, 128.3, 128.2, 128.1, 128.0, 127.9, 127.9, 127.8, 127.7, 127.7, 127.6, 127.6, 127.6, 127.5, 100.9 (anomeric, J_{CH} = 173 Hz), 100.0 (anomeric, J_{CH} = 170

Hz), 98.8 (anomeric, J_{CH}= 171 Hz), 97.1 (anomeric, J_{CH} = 183 Hz), 91.2 (CCl_3), 80.4, 80.0, 78.9, 75.6, 75.3, 75.2, 75.1, 75.0, 74.6, 74.22, 73.9, 73.5, 73.4, 72.8, 72.6, 72.4, 72.4, 72.4, 72.2, 69.3 (CH_2), 69.1 (CH_2), 68.9 (CH_2), 62.7 (CH_2), 38.1 (-CH_2- Lev), 29.9 (-CH_3 Lev), 28.2 (-CH_2- Lev), 18.4 (CH3- TIPS), 12.2 (-CH- TIPS); ESI-HRMS: m/z $[M+Na]^+$ cald 1638.6243, obsd 1638.6320.

Allyl 2-3,4,6-tetra-O-benzyl-α-D-mannopyranosyl-(1→2)-3,4-di-O-benzyl-6-O-triisopropyl silyl-α-D-mannopyranosyl-(1→2)-3,4,6-tri-O-benzyl-α-D-mannopyranosyl-(1→6)-2-O-levulonoyl-3-O-benzyl-4-O-(2-(naphthyl)methyl-α-D-mannopyranosyl-(1→4)-2-azido-3,6-di-O-benzyl-2-deoxy-α-D-glucopyranosyl-(1→6)-1-O-allyl-2(2-napthylmethyl)-3,4,5-tetra-O-benzyl-D-myo-inositol **(5)**

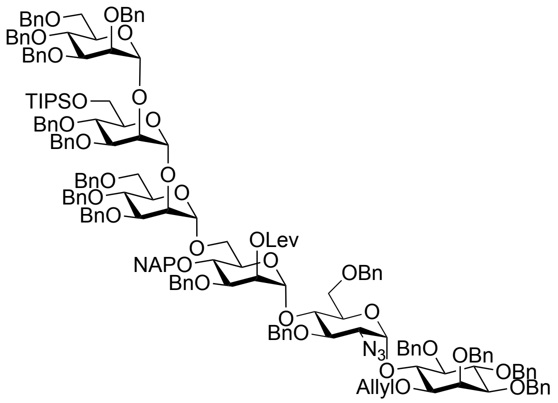

A mixture of glycosyl acceptor **16**[7] (0.519 g, 0.547 mmol) and glycosyl donor **15** (1.21 g, 0.574 mmol) was dissolved and co-evaporated with dry toluene three times and dried under high vacuum for 2 h. The mixture was dissolved in anhydrous CH_2Cl_2 (6 mL) under argon atmosphere followed by the addition of activated powdered molecular sieves. Then, TBSOTf (19 µL, 0.082 mmol) was added to the reaction mixture at -20 °C. After 2 h, the reaction mixture was quenched with triethylamine, filtered and concentrated. The crude mixture was purified using column chromatography to obtain **5** (1.45 g, 0.501 mmol, 92%). ^{1}H NMR (400 MHz, cdcl$_3$) δ 7.82 – 7.77 (m, 1H), 7.66 – 7.63 (m, 2H), 7.56 (dd, *J* = 7.5, 1.4 Hz, 1H), 7.48 – 7.02 (m, 82H), 6.93 (t, *J* = 7.4 Hz, 1H), 5.99 – 5.88 (m, 1H, -$\underline{CH}$=CH_2 allyl), 5.71 (d, *J* = 3.7 Hz, 1H), 5.46 (t, *J* = 2.2 Hz, 1H), 5.38 (d, *J* = 1.7 Hz, 1H, anomeric), 5.32 – 5.24 (m, 2H, anomeric, $C\underline{H}_{2b}$= allyl), 5.23 – 5.17 (m, 2H, $\underline{CH}_{\underline{2a}}$= allyl, anomeric), 5.03 (dd, *J* = 11.6, 7.9 Hz, 2H), 4.96 – 4.76 (m, 10H), 4.76 – 4.62 (m, 7H), 4.61 – 4.18 (m, 23H), 4.17 – 3.72 (m, 25H), 3.67 – 3.52 (m, 3H), 3.51 – 3.30 (m, 5H), 3.29 – 3.18 (m, 4H), 3.12 (dd, *J* = 11.4, 3.0 Hz, 1H), 2.67 – 2.36 (m, 4H), 2.02 (s, 3H), 1.01 (d, *J* = 4.7 Hz, 21H). ^{13}C NMR (101 MHz, $CDCl_3$) δ 205.7 (C=O ketone), 171.9 (C=O ester), 128.6, 128.5, 128.5, 128.4, 128.3, 128.3, 128.2, 128.2, 128.1, 128.1, 128.0, 128.0, 127.9, 127.8, 127.8, 127.7, 127.6, 127.6, 127.5, 127.4, 127.4, 127.3, 127.2, 126.0, 125.6, 125.4, 117.1 (=$\underline{CH_2}$), 100.2 (anomeric), 99.6 (anomeric), 99.2 (anomeric), 99.1 (anomeric), 97.6 (anomeric), 81.8, 81.2, 80.9, 80.3, 80.1, 80.0, 79.8, 78.6, 75.8, 75.1, 75.0, 74.9, 74.7, 74.5, 74.4, 74.3, 73.3, 73.1, 72.5, 72.3, 72.1, 72.0, 71.6, 71.4, 71.2, 70.3, 69.1 (CH_2- Bn), 68.9 (CH_2-), 68.7 (CH_2-), 64.2 (CH_2-), 62.6 (CH_2-), 37.8 (-CH_2- Lev), 30.0 (-CH_3 Lev), 28.3 (CH_2- Lev), 18.14 (CH_3- TIPS), 11.7 (CH- TIPS). ESI-MS (*m/z*): $[M+H]^+$ cald 2891.349 obsd 2891.319.

3,4,6-tri-O-benzyl-α-D-mannopyranosyl-(1→2)-3,4-di-O-benzyl-6-O-triisopropylsilyl-α-D-mannopyranosyl-(1→2)-3,4,6-tri-O-benzyl-α-D-mannopyranosyl-(1→6)-2-O-levulinyl-3-O-benzyl-4-O-(2-napthyl)methyl-α-D-mannopyranosyl-(1→4)-2-azido-3,6-di-O-benzyl-2-deoxy-α-D-glucopyranosyl-(1→6)-2,3,4,5-tetra-O-benzyl-D-myo-inositol **(6)**

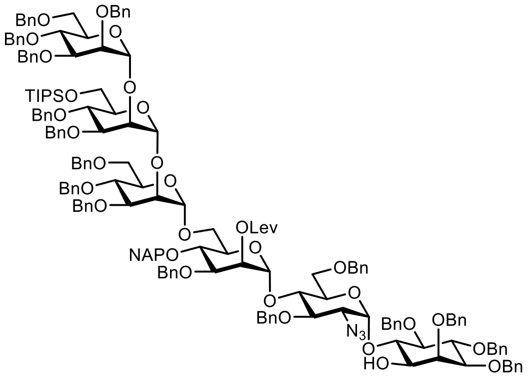

To a stirred solution of *pseudo*-hexasaccharide **5** (0.130 g, 0.045 mmol) in acetic acid (2 mL) and water (0.1 mL) were added palladium chloride (0.04 g, 0.23 mmol) and sodium acetate (0.04 g, 0.45 mmol) at room temperature. After 16 h, the reaction mixture was diluted with dichloromethane and the solution was washed with aqueous NaHCO3, brine, and water. The combined organic layers were dried over Na_2SO_4, filtered and concentrated. The crude product was purified by silica gel column chromatography to give *pseudo*-hexasaccharide **6** (90 mg, 0.032 mmol, 70%) as colorless oil. R_f= 0.34 (n-hexan/ AcOEt, 2:1), ^{1}H NMR (400 MHz, $CDCl_3$) δ 7.76 (dd, *J* = 7.8, 1.7 Hz, 1H, Ar), 7.63 (d, *J* = 8.6 Hz, 2H, Ar), 7.56 (dd, *J* = 7.7, 1.9 Hz, 1H, Ar), 7.45 – 7.01 (m, 72H, Ar), 6.95 – 6.89 (m, 1H, Ar), 5.46 – 5.42 (m, 2H, anomeric, CH_{2b}- Bn), 5.36 (d, *J* = 1.8 Hz, 1H, anomeric), 5.29 (d, *J* = 1.8 Hz, 1H, anomeric), 5.20 (bs, 1H, anomeric), 5.00 (dt, *J* = 11.1, 6.9 Hz, 3H, CH_{2a}- , CH_2- Bn), 4.93 – 4.82 (m, 4H), 4.81 – 4.66 (m, 9H), 4.61 – 4.36 (m, 15H), 4.32 – 4.22 (m, 4H), 4.20 – 4.17 (m, 2H), 4.13 (dd, *J* = 4.2, 1.9 Hz, 2H), 4.07 (td, *J* = 9.2, 2.2 Hz, 2H), 4.02 – 3.55 (m, 21H), 3.46 (dd, *J* = 9.9, 2.2 Hz, 2H), 3.42 – 3.33 (m, 3H), 3.28 (td, *J* = 9.0, 8.5, 4.0 Hz, 2H), 3.24 – 3.20 (m, 2H), 3.00 (d, *J* = 7.1 Hz, 1H, O$\underline{H}$), 2.59 – 2.34 (m, 4H, 2 x CH_2- Lev), 2.00 (s, 3H, CH_3 Lev), 1.00 (m, 21H, $C\underline{H_3}$, C$\underline{H}$ TIPS). ^{13}C NMR (101 MHz, $CDCl_3$) δ 205.7 (C=O ketone), 171.8 (C=O ester), 139.0, 138.9, 138.8, 138.6, 138.5, 138.4, 138.4, 138.1, 137.9, 137.8, 137.6, 136.38, 133.3, 132.8, 128.6, 128.5, 128.4, 128.4, 128.3, 128.3, 128.3, 128.2, 128.1, 128.1, 128.0, 128.0, 127.9, 127.8, 127.8, 127.7, 127.6, 127.6, 127.5, 127.4, 127.4, 127.3, 127.2, 126.0, 125.6, 125.4, 100.1 (anomeric), 99.6 (anomeric), 99.0 (anomeric), 98.8, (anomeric) 97.8 (anomeric), 81.9, 81.2, 80.9, 80.3, 70.0, 79.9, 79.7, 78.6, 75.8, 75.1, 75.0, 74. 9, 74.7, 74.5, 74.4, 74.3, 73.3, 73.1, 72.5, 72.3, 72.1, 72.0, 71.6, 71.4, 71.2, 70.3, 68.9, 68.7, 64.3, 62.6, 37.8 (-CH_2- Lev), 31.5, 30.2 (-CH_3 Lev), 29.8, 27.9 (-CH_2- Lev), 22.73, 18.8 ($\underline{CH_3}$-CH TIPS, 11.9. (-CH- TIPS), ESI-MS (*m*/*z*): $[M+H]^+$ cald 2851.325 obsd 2851.429.

1,2-di-O-stearoyl-sn-glycero-3-H-phosphonate **(7)**[8]

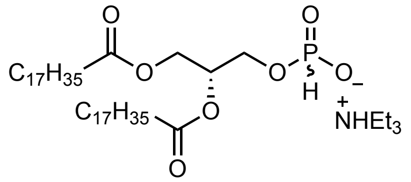

A mixture of 1,2-distearoyl-*sn*-glycerol (250 mg, 0.40 mmol) and phosphonic acid (36.1 mg, 0.44 mmol) were dissolved and co-evaporated three times with pyridine. The resulting mixture was dissolved in anhydrous pyridine (10 mL) and pivaloyl chloride (0.05 mL, 0.32 mmol) was added dropwise.[9] The reaction mixture was stirred under nitrogen atmosphere at room temperature for two days. The volatiles were removed under reduced pressure and the resulting solid was purified by silica gel column chromatography to give H-phosphonate **7** (218 mg, 0.32 mmol, 79%) as a white solid. R*f*= 0.56 (DCM/MeOH, 10:1), ^{1}H NMR (400 MHz, $CDCl_3$) δ 12.50 (s, 1H, N<u>H</u>, ammonium), 6.79 (d, *J* = 623.4 Hz, 1H, P-<u>H</u>), 5.15 (qd, *J* = 5.3, 3.4 Hz, 1H, CH glycerol), 4.31 (dd, *J* = 12.0, 3.6 Hz, 1H, CH_{2b} glycerol), 4.11 (dd, *J* = 11.9, 6.4 Hz, 1H, CH_{2a} glycerol), 3.93 (dd, *J* = 7.9, 5.2 Hz, 2H, CH_2 glycerol), 3.00 (qd, *J* = 7.3, 4.2 Hz, 6H, -CH_2- TEA), 2.32 – 2.15 (m, 4H, CH_2 lipid), 1.52 (tq, *J* = 7.5, 3.5 Hz, 4H, -CH_2- lipid), 1.23 (d, *J* = 34.3 Hz, 65H, lipid), 0.81 (t, *J* = 6.7 Hz, 6H, 2 x CH_3- lipid). ^{13}C NMR (101 MHz, $CDCl_3$) δ = 173.5 (C=O ester), 173.1 (C=O ester), 70.5, 70.4, 62.6, 62.0, 50.8, 45.4, 34.3, 34.1, 32.0, 29.7, 29.7, 29.7, 29.5, 29.4, 29.4, 29.3, 29.2, 29.2, 24.9, 24.9, 22.7, 14.2, 8.5. ^{31}P NMR (162 MHz, $CDCl_3$) δ 4.83. ESI-MS (*m*/*z*): $[M+H]^+$ cald 688.541 obsd 688.712

3,4,6-tri-O-benzyl-α-D-mannopyranosyl-(1→2)-3,4-di-O-benzyl-6-O-triisopropylsilyl-α-D-mannopyranosyl-(1→2)-3,4,6-tri-O-benzyl-α-D-mannopyranosyl-(1→6)-2-O-levulinyl-3-O-benzyl-4-O-(2-napthyl)methyl-α-D-mannopyranosyl-(1→4)-2-azido-3,6-di-O-benzyl-2-deoxy-α-D-glucopyranosyl-(1→6)-1-O-(1,2-dimyristoyl-sn-glyceryl-phosphonato)-2,3,4,5-tetra-O-benzyl-D-myo-inositol **(8)**

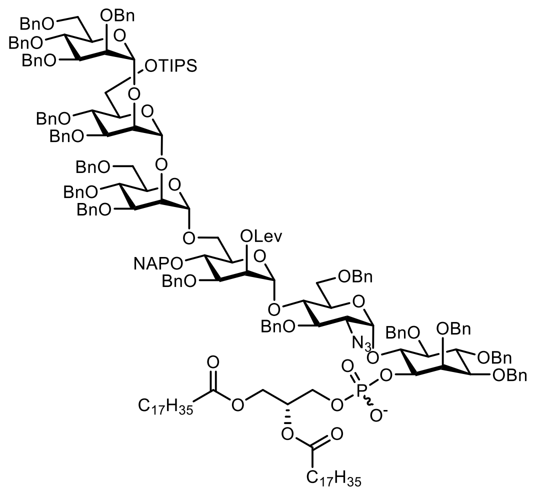

The *pseudo*-pentasaccharide **6** (98 mg, 0.041 mmol) and H-phosphonate **7** (161 mg, 0.203 mmol) were dissolved and co-evaporated with anhydrous pyridine (3 x 3 mL) and dried under high vacuum overnight. The reaction mixture was dissolved in anhydrous pyridine (2 mL) at room temperature and pivaloyl chloride (20 μL, 0.163 mmol) was added.[9] The resulting

reaction mixture was stirred at room temperature for 3 h. Iodine (36.3 mg, 0.143 mmol) in a mixture of pyridine/water (19:1, 0.2 mL) was added to oxidize P (III) to P (V). The reaction mixture was further stirred for 2 h at room temperature to the complete the oxidation. The reaction mixture was diluted with $CHCl_3$ and washed with aqueous $Na_2S_2O_3$ solution to remove the excess iodine. The aqueous layer was further washed with $CHCl_3$ and the combined organic layer was dried over Na_2SO_4, filtered and concentrated under reduced pressure to give the crude residue, which was subjected to flash column with Et_3N-deactivated silica gel to give **8** (70 mg, 55 %) as a syrup. R_f = 0.25 ($CHCl_3$/MeOH = 20: 1). ^{1}H NMR (400 MHz, Chloroform-*d*) δ 7.71 (d, *J* = 7.9 Hz, 1H, Ar), 7.56 (t, *J* = 4.1 Hz, 1H, Ar), 7.48 (d, *J* = 7.9 Hz, 1H, Ar), 7.40 – 6.92 (m, 61H, Ar), 6.84 (t, *J* = 7.4 Hz, 1H, Ar), 5.81 (d, *J* = 3.7 Hz, 1H, anomeric GlcN), 5.34 (bs, 1H anomeric), 5.24 (m, 2H, anomeric, CH_2- Bn), 5.15 (d, *J* = 10.8 Hz, 1H, CH_2- Bn), 5.00 – 4.23 (m, 27H), 4.19 (d, *J* = 12.1 Hz, 1H), 4.15 – 3.64 (m, 15H), 3.63 – 3.32 (m, 7H), 3.33 – 3.11 (m, 3H), 3.04 (dd, *J* = 10.1, 3.8 Hz, 1H), 2.47 – 2.30 (m, 2H), 2.27 – 2.13 (m, 3H, CH_3-Lev), 1.93 (d, *J* = 7.4 Hz, 4H, -CH_2- lipid), 1.53 – 1.40 (m, 4H, -CH_2- lipid)), 1.27 – 1.14 (m, 96H, lipi , CH_3-, amonium), 0.94 (m, 21H, TIPS), 0.81 (t, *J* = 6.7 Hz, 6H, 2 x CH_3-, lipid). ^{13}C NMR (101 MHz, $CDCl_3$) δ = 205.3 (C=O ketone), 172.8 (C=O ester), 172.4 (C=O ester), 171.4 (C=O ester), 138.8, 138.5, 138.4, 138.3, 138.2, 138.1, 138.0, 137.9, 137.9, 137.9, 137.8, 137.8, 137.7, 137.6, 137.6, 137.5, 137.3, 136.0, 133.0, 132.5, 128.1, 128.0, 128.0, 127.9, 127.9, 127.9, 127.8, 127.8, 127.8, 127.7, 127.7, 127.6, 127.6, 127.6, 127.5, 127.5, 127.4, 127.4, 127.3, 127.3, 127.3, 127.2, 127.1, 127.0, 127.0, 126.9, 126.9, 126.8, 126.8, 126.7, 125.6, 125.3, 125.1, 99.9 (anomeric), 98.7 (anomeric), 98.6, 98.3, 97.3, 81.4, 81.3, 80.3, 80.2, 80.0, 79.8, 79.4, 78.1, 75.9, 75.5, 75.1, 75.0, 74.7, 74.7, 74.6, 74.4, 74.3, 74.2, 74.2, 73.9, 73.7, 73.5, 73.3, 73.0, 73.0, 72.8, 72.6, 72.3, 71.7, 71.6, 71.6, 71.4, 71.3, 70.9, 69.9, 68.9, 68.7, 68.6, 68.0, 65.9, 63.5, 63.0, 62.6, 61.1, 37.5 (CH_2- Lev), 33.8, 33.6, 31.6, 29.4 (CH_3- Lev), 29.2, 29.0, 28.9, 28.8, 27.7 (CH_2-Lev), 26.7, 24.5, 22.3, 17.7 (CH3- TIPS), 13.8 (CH_3- Lipid), 11.8 (-CH- TIPS), ^{31}P NMR (162 MHz, $CDCl_3$) δ -1.58. ESI-MS (*m/z*): $[M-H]^-$ cald 3535.835 obsd 3535.044.

3,4,6-tri-O-benzyl-α-D-mannopyranosyl-(1→2)-3,4-di-O-benzyl-6-O-(2-(N-(tert-butoxycarbonyl)-S-(tert-butyl)-L-cysteinyl)aminoethyl phosphonato)-α-D-mannopyranosyl-(1→2)-3,4,6-tri-O-benzyl-α-D-mannopyranosyl-(1→6)-2-O-levulinyl-3,4-di-O-benzyl-α-D-mannopyranosyl-(1→4)-2-azido-3,6-di-O-benzyl-2-deoxy-α-D-glucopyranosyl-(1→6)-1-O-(1,2-dimyristoyl-sn-glyceryl-phosphonato)-2,3,4,5-tetra-O-benzyl-D-myo-inositol **(11)**

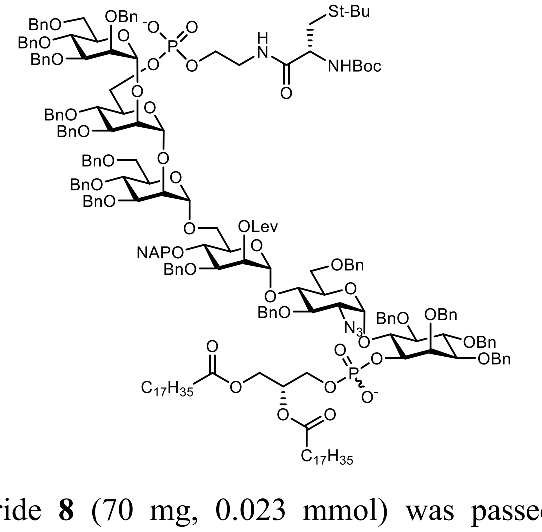

The *pseudo*-pentasaccharide **8** (70 mg, 0.023 mmol) was passed through a column of Amberlite IR120 ion exchange resin (Na^+ form) using $CHCl_3$/MeOH (1:1) as eluent. The eluate was collected and concentrated to dryness. The obtained residue was then dissolved in CH_3CN

(2.5 mL) with a trace of H_2O (100 µL). $Sc(OTf)_3$ (55.6 mg, 0.113 mmol) was added to the reaction mixture and the resulting mixture was heated to 45 ºC and stirred overnight. The reaction mixture was cooled down to room temperature and few drops of pyridine was added to quench any excess $Sc(OTf)_3$. The reaction mixture was evaporated to dryness and subjected to flash column chromatography with Et_3N-deactivated silica gel to afford the alcohol **9** (45 mg, 68 %) as a syrup. The product was directly used in the next phosphorylation step. ESI-MS (*m*/*z*): $[M-H]^-$ cald 3380.709 obsd 3380.025.

The bilipidated *pseudo*-pentasaccharide **9** (40 mg, 0.014 mmol) and *H*-phosphonate **10**[10] (39 mg, 0.102 mmol) were dissolved and co-evaporated with anhydrous pyridine (3x3 mL) and dried under high vacuum overnight. The reaction mixture was dissolved in anhydrous pyridine (2 mL) at room temperature and pivaloyl chloride (13 µL, 0.102 mmol) was added. The resulting reaction mixture was stirred at room temperature for 5 h. The progress of the reaction was monitored by using TLC analysis. Iodine (26 mg, 0.102 mmol) in a mixture of pyridine/water (19:1, 0.2 mL) was added to oxidize P (III) to P (V). The reaction mixture was further stirred for 2 h at room temperature to complete the oxidation, diluted with $CHCl_3$, washed with aqueous $Na_2S_2O_3$ solution to remove the excess iodine. The aqueous layer was further washed with $CHCl_3$ and the combined organic layer was dried over Na_2SO_4, filtered and concentrated under reduced pressure to give the crude residue, which was subjected to flash column with Et_3N-deactivated silica gel to give **11** (32 mg, 70 %) as a syrup. ^{1}H NMR (400 MHz, Chloroform-*d*) δ 12.18 ($^+$N<u>H</u>- Amonium), 7.71 (d, *J*=7.6 Hz, Ar)), 7.34 (d, *J* = 7.3 Hz, 6H, Ar), 7.25 (d, *J* = 14.9 Hz, Arf), 7.25 – 7.09 (m, 74H), 7.02 (dd, *J* = 22.1, 10.4 Hz, 22H), 5.81 (d, J< 1 Hz, 1H, anomeric) 5.38 – 5.02 (m, 4H, 3x anomeric, CH_{2b}- Bn), 4.83 (m, 16H, 1x anomeric, CH_2 Bn), 4.63 – 4.48 (m, 9H), 4.48 – 4.25 (m, 21H), 4.24 – 3.87 (m, 17H), 3.81 (m, 8H), 3.77 – 3.65 (m, 6H, -CH_2- TEA), 3.59 (d, *J* = 9.9 Hz, 3H), 3.44 (dt, *J* = 40.4, 14.9 Hz, 10H), 3.29 – 2.99 (m, 4H), 2.81 (s, 8H), 2.26 – 2.10 (m, 4H, 2 X -CH_2- Lev), 2.01 (s, 3H, CH_3-Lev), 1.95 (m, 4H, -CH_2-Lipid), 1.20 – 1.07 (m, 62H CH_2-Lipid), 0.97 (dd, *J* = 26.0, 12.1 Hz, 12H (CH_3- TEA)), 0.81 (t, *J* = 6.7 Hz, 6H, 2 x CH_3- Lipid). ^{13}C NMR (101 MHz, $CDCl_3$) δ 205.9 (C=O Ketone), 173.4 (C=O ester lipid), 173.0 (C=O ester lipid), 171.9 (C=O ester), 139.0, 138.9, 138.8, 138.6, 138.5, 138.4, 138.4, 138.1, 137.9, 137.8, 137.6, 136.38, 133.3, 132.8, 128.6, 128.5, 128.4, 128.4, 128.3, 128.3, 128.3, 128.2, 128.1, 128.1, 128.0, 128.0, 127.9, 127.8, 127.8, 127.7, 127.6, 127.6, 127.5, 127.4, 127.4, 127.3, 127.2, 126.0, 125.6, 125.4, 100.7 (anomeric), 99.6 (anomeric), 99.3 (anomeric), 98.8 (anomeric), 96.7 (anomeric), 81.9, 81.2, 80.9, 80.3, 70.0, 79.9, 79.7, 78.6, 75.8, 75.1, 75.0, 74. 9, 74.7, 74.5, 74.4, 74.3, 73.3, 73.1, 72.5, 72.3, 72.1, 72.0, 71.6, 71.4, 71.2, 70.3, 68.9, 68.7, 64.3, 62.6, 37.8 (-CH_2- Lev), 31.5, 30.2 (-CH_2- Lev), 29.9, 29.8, 29.8, 29.7, 29.6, 29.4, 29.3, 29.2, 28.0, 27.9 (-CH_2- Lev), 25.0, 24.9, 24.9, 22.7, 22.73, 18.8 (<u>CH_3</u>-CH TIPS), 18.1, 14.2, 11.9 (-CH- TIPS), 8.5, ^{31}P NMR (162 MHz, $CDCl_3$) δ -1.42, -1.51. ESI-MS (*m*/*z*): $[M-H]^-$ cald 3761.835, $[M-H]^-$ obsd 3761.983.

α-D-mannopyranosyl-(1→2)-6-O-(N-(S-S-mercaptoethanol-L-cysteinyl)aminoethanol-phosphonato)-α-D-mannopyranosyl-(1→2)-α-D-mannopyranosyl-(1→6)-α-D-

mannopyranosyl-(1→4)-2-amino-2-deoxy-α-D-glucopyranosyl-(1→6)-1-O-(1,2-dimyristoyl-sn-glyceryl-phosphonato)-D-myo-inositol (GPI**3**)

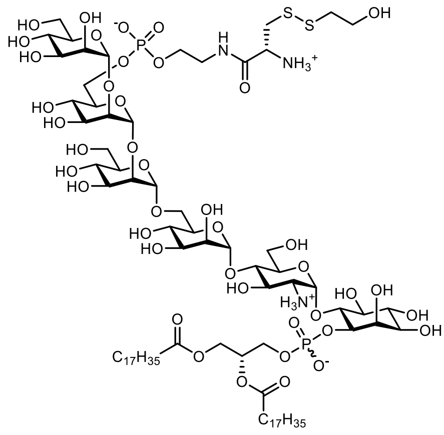

To the stirred solution of bisphosphorylated *pseudo*-hexasaccharide **11** (18.0 mg, 0.005 mmol) in CH_2Cl_2 (2 mL) were added acetic acid (10 µL, 0.19 mmol), pyridine (15 µL, 0.19 mmol) and hydrazine hydrate (2.3 µL, 0.05 mmol) sequentially. The reaction mixture was stirred at room temperature. After 12 h, the reaction mixture was quenched with acetone and concentrated to obtain crude product. The crude product was then dissolved in mixture of CH_2Cl_2/MeOH (3:1, 5 mL) at room temperature and $Pd(OH)_2$/C (33 mg, 0.046 mmol, 20% Pd content) was added. Hydrogen gas was bubbled through the solution for 15 min and the reaction mixture was stirred under an atmosphere of hydrogen gas for additional 16 h. The palladium was removed by filtration through a pad of celite and the solvents were removed under reduced pressure to give the crude product. ^{1}H NMR (400 MHz, $CDCl_3$/MeOD) δ 7.48 (m, 3H, NH, PEtN, cysteine), 5.4a (bs anomeric), 5.13 (m, anomeric), 4.95 (d, *J* = 46.0 Hz, 4H, anomeric), 4.10 – 3.92 (m, 14H), 3.90 (s, 9H), 3.79 (dd, *J* = 18.2, 13.0 Hz, 10H), 3.71 – 3.31 (m, 26H), 3.15 – 2.91 (m, 2H), 2.86 – 2.68 (m, 2H), 2.63 – 2.45 (m, 2H, CH_2-), 2.31 – 2.09 (m, 6H), 1.55 – 1.44 (m, 7H, CH2- lipid), 1.27 – 1.15 (m, 74H, lipid, tBu), 0.79 (m, 17H, tBu, CH3- lipid). ^{31}P NMR (162 MHz, MeOD) δ 0.48, 0.11.

The debenzylated crude intermediate compound was confirmed by mass spectrometry. The crude material (4 mg) was then dissolved at 0 °C in a mixture of trifluoroacetic acid and anisole (5.5 mL, 10:1 v/v) and stirred for 5 min. Then $Hg(TFA)_2$ (9.1 mg, 21 µmol) was added to the reaction mixture and stirred for an additional 30 min. the volatiles were removed under high vacuum at 0 °C. The resulting solid residue was dissolved in an AcOH/water mixture (7.5 mL, 7:3 v/v) and mercaptoethanol (400 µL, 5.7 mmol) was added at room temperature. The mixture was stirred at the same temperature for 10 h. The solution was dried on a rotary evaporator, to give a pale-yellow residue. The amphiphilic nature of GPI**3** (2.5 mg, 1.2 µmol, 41%) created solubility problem in only one solvent. So, the NMR was analysed in mixture of chloroform, methanol and water. ^{1}H NMR (400 MHz, $CDCl_3$/MeOD) δ 7.32 (d, *J* = 7.1 Hz, 1H), 7.25 – 7.17 (m, 2H, NH_2), 5.42 (s, 1H, anomeric), 5.14 (m, 3H, 2 x anomeric), 5.01 (s, 1H, anomeric), 4.89 (s, 1H), 4.16 – 3.42 (m, 77H), 3.41 – 3.31 (m, 0H), 3.11 – 3.00 (m, 1H), 2.30 – 2.18 (m, 2H, CH2- Cys), 2.18 – 2.01 (m, 2H), 1.93 (d, *J* = 10.0 Hz, 1H), 1.57 – 1.31 (m, 8H, -CH2- lipid), 1.28 – 1.11 (m, 58H, lipid), 0.82 – 0.75 (m, 6H, CH_3, lipid). ^{31}P NMR (101 MHz, $CDCl_3$/MeOD) δ 0.27, -0.03. ESI-MS (*m*/*z*): $[M-2H]^{2-}$ cald 987.9350 obsd 988.0052.

*2,3,4-Tri-O-benzyl-6-O-triisopropylsilyl-α-D-mannopyranosyl-(1→2)-3,4,6-tri-O-benzyl-α-D-mannopyranosyl-(1→6)-2,3-di-O-benzyl-4-O-(2-naphthyl)methyl-α-D-mannopyranosyl-(1→4)-2-azido-3,6-di-O-benzyl-2-deoxy-α-D-glucopyranosyl-(1→6)-1-O-allyl-2,3,4,5-tetra-O-benzyl-D-myo-inositol (**13**)*

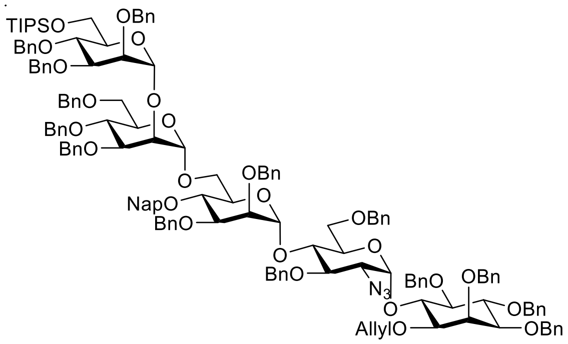

The Synthesis of the GPI**2** started with the removal of the levulinic ester from the pseudopentasaccharide **12**.[5] The pseudopentsaccharide **4** (0.146 g, 0.062 mmol) was dissolved in methanol/CH_2Cl_2 (7:3) followed by addition of (5 mg, 0.092 mmol) of sodium methoxide. The reaction was stirred at room temperature keeping the pH above 8.0. After completion of the reaction by TLC, The reaction was quenched using Amberlite IR120 H+ form. The reaction mixture was filtered and the product was directly benzylated. The residue was dissolved in THF (2 mL) at 0 °C, NaH (6 mg, 0.247 mmol) and 15-crown-5 (0.014 g, 0.062 mmol) were added. The reaction mixture was stirred for 30 min and benzyl bromide (BnBr) (0.022 ml, 0.185 mmol) and DMF (2 mL) were added to the reaction mixture. The reaction mixture was stirred at room temperature for 2 h. The reaction mixture was cooled down to 0 °C, quenched by the addition of H_2O dropwise and the solvent was evaporated using rotavapor. The residue was extracted using ethyl acetate and water. The organic layer was dried over anhydrous sodium sulfate, filtered and concentrated. The residue was purified using silica gel column chromatography to obtain the pseudopentaccharide **13** (0.130 g, 86 %). R*f*= 0.30 (n-hexan/ AcOEt, 3:1), ^{1}H NMR (400 MHz, Chloroform-*d*) δ 7.73 – 7.70 (m, 1H, Ar), 7.65 – 7.59 (m, 2H, Ar), 7.56 (dd, *J* = 7.4, 2.0 Hz, 1H, Ar), 7.39 – 7.31 (m, 4H, Ar), 7.31 – 6.95 (m, 70H, Ar), 5.84 (ddt, *J* = 17.4, 10.6, 5.5 Hz, 1H, CH= allyl), 5.69 (d, *J* = 3.7 Hz, 1H, H-1 GlcN), 5.23 – 5.16 (m, 2H, anomeric, CH_{2b}= allyl), 5.14 (d, *J* = 1.8 Hz, 1H, anomeric), 5.10 (dq, *J* = 10.4, 1.4 Hz, 1H, CH_{2a}= allyl), 5.04 (d, *J* = 11.7 Hz, 1H), 4.95 – 4.80 (m, 4H), 4.77 (s, 2H), 4.71 – 4.49 (m, 11H, anomeric), 4.47 – 4.16 (m, 15H), 4.13 (d, *J* = 10.7 Hz, 1H), 4.09 – 3.85 (m, 9H), 3.85 – 3.71 (m, 7H), 3.65 – 3.48 (m, 7H), 3.39 – 3.13 (m, 9H), 0.96 (m, 21H, CH_3-, CH TIPS). ^{13}C NMR (101 MHz, $CDCl_3$) δ = 139.8, 138.1, 137.8, 137.7, 137.6, 137.5, 137.5, 137.4, 137.3, 137.2, 137.2, 136.9, 136.8, 135.5, 133.2 (CH= allyl), 132.3, 131.7, 127.5, 127.5, 127.4, 127.4, 127.3, 127.3, 127.2, 127.2, 127.1, 127.1, 127.0, 127.0, 127.0, 126.9, 126.8, 126.8, 126.7, 126.7, 126.6, 126.6, 126.5, 126.5, 126.3, 126.2, 126.2, 126.1, 126.1, 126.1, 126.1, 126.0, 125.8, 124.9, 124.8, 124.6, 124.4, 124.3, 116.1 (CH_2= allyl), 99.4 (anomeric), 98.1 (anomeric), 97.3 (anomeric), 96.5 (anomeric), 80.8, 80.4, 79.8, 79.1, 79.0, 78.8, 78.6, 76.2, 75.1, 74.8, 74.4, 74.2, 74.0, 73.9, 73.8, 73.5, 73.3, 73.0, 72.7, 72.6, 72.2, 72.1, 71.8, 71.6, 71.5, 71.1, 71.0, 70.9, 70.8, 70.8, 70.7, 70.4, 69.7, 68.6, 68.0, 67.5, 65.2, 64.3, 62.2, 61.8, 17.0 (CH_3- TIPS), 17.0 (CH_3- TIPS), 11.0 (CH- TIPS). MALDI-MS (*m*/*z*): $[M+Na-N_2]^+$ cald 2445.1489, obsd 2445.080.

*2,3,4-Tri-O-benzyl-6-O-triisopropylsilyl-α-D-mannopyranosyl-(1→2)-3,4,6-tri-O-benzyl-α-D-mannopyranosyl-(1→6)-2,3-di-O-benzyl-4-O-(2-naphthyl)methyl-α-D-mannopyranosyl-(1→4)-2-azido-3,6-di-O-benzyl-2-deoxy-α-D-glucopyranosyl-(1→6)-2,3,4,5-tetra-O-benzyl-D-myo-inositol **(14)***

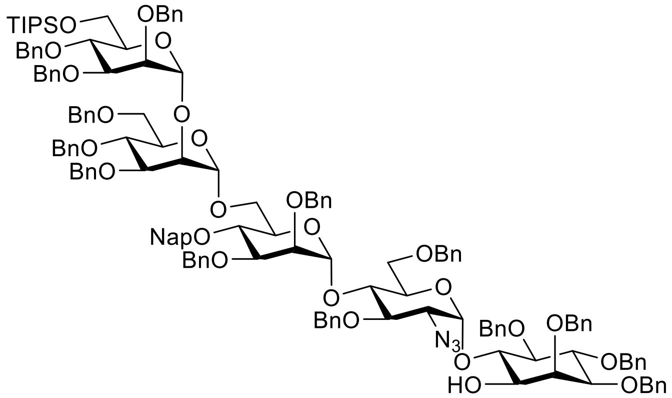

A solution of [IrCOD(PPh2Me)2]PF6 (15.0 mg) in THF (1 mL) was stirred under hydrogen until the color turned from red to colorless to pale yellow. The hydrogen atmosphere was exchanged with Argon. This solution was added to a solution of pseudopentasaccharide **13** (0.130 g, 0.053 mmol) in THF (2 mL). After 16 h, the solvent was removed and the residue was dissolved in a mixture of acetone (2.7 mL) and water (0.3 mL). Mercury (II) chloride (72 mg, 0.265 mmol) and mercury (II) oxide (2.5 mg, 10.60 µmol) were added. After 1 h, saturated $NaHCO_3$ (aq) was added and the reaction mixture was extracted three times with dichloromethane. The combined organic layers were dried over Na_2SO_4, filtered and concentrated. The crude product was purified by silica gel column chromatography to give pseudopentasaccharide **14** (100 mg, 0.041 mmol, 78 % yield) as colorless oil. R_f= 0.25 (n-hexan/ AcOEt, 3:1),^{1}H NMR (400 MHz, $CDCl_3$) δ 7.73 – 7.69 (m, 1H, Ar), 7.63 – 7.60 (m, 2H, Ar), 7.58 – 7.55 (m, 1H, Ar), 7.35 (ddd, *J* = 7.2, 4.9, 1.8 Hz, 2H, Ar), 7.31 – 6.99 (m, 65H, Ar), 5.44 (d, *J* = 3.6 Hz, 1H, anomeric), 5.22 (d, *J* = 2.2 Hz, 1H, anomeric), 5.15 (d, *J* = 1.8 Hz, 1H, anomeric), 5.01 (d, *J* = 11.8 Hz, 1H, C$\underline{H2}$ - Bn), 4.94 (d, *J* = 11.6 Hz, 1H), 4.90 – 4.80 (m, 4H, H1 GlcN, CH_2- Bn), 4.71 – 4.54 (m, 11H), 4.42 (d, *J* = 5.1 Hz, 2H), 4.39 – 4.18 (m, 13H), 4.15 – 3.70 (m, 18H), 3.67 – 3.52 (m, 8H), 3.40 (dd, *J* = 9.9, 2.3 Hz, 1H, C$\underline{H}$2- Man), 3.36 – 3.22 (m, 7H), 2.91 (bs, 1H, O$\underline{H}$), 0.95 (m, 21H, TIPS). ^{13}C NMR (101 MHz, $CDCl_3$) δ 139.0, 138.7, 138.7, 138.5, 138.5, 138.4, 138.4, 138.4, 138.4, 138.2, 138.1, 138.0, 138.0, 137.6, 136.4, 133.3, 132.8, 129.5, 128.5, 128.4, 128.4, 128.3, 128.3, 128.2, 128.2, 128.1, 128.1, 128.1, 128.0, 127.9, 127.9, 127.8, 127.8, 127.8, 127.8, 127.7, 127.7, 127.7, 127.6, 127.5, 127.5, 127.5, 127.4, 127.4, 127.3, 127.3, 127.2, 127.2, 126.9, 125.9, 125.6, 125.5, 125.5, 100.1(H-1 Man), 99.2 (H-1 GlcN), 98.3 (H-1 Man), 97.6 (H-1 Man), 81.7, 81.3, 80.9, 80.5, 80.2, 79.6, 76.0, 75.8, 75.3, 75.0, 75.0, 74.9, 74.8, 74.5, 74.4, 74.2, 73.7, 73.3, 73.2, 73.1, 73.0, 72.5, 72.2, 72.1, 72.0, 71.9, 71.5, 70.4, 69.0 (CH_2 GlcN), 68.7 (CH_2 Man), 64.0 (CH_2 Man), 62.9 (CH_2 Man), 31.9, 31.4, 30.2, 29.7, 29.6, 29.3, 22.7, 18.0 (CH3- TIPS), 12.0 (-CH- TIPS). MALDI-MS (*m/z*): $[M+K-N_2]^+$ cald 2421.0951, obsd 2421.419.

2,3,4-Tri-O-benzyl-α-D-mannopyranosyl-(1→2)-3,4,6-tri-O-benzyl-α-D-mannopyranosyl-(1→6)-2,3-di-O-benzyl-4-O-(2-naphthyl)methyl-α-D-mannopyranosyl-(1→4)-2-azido-3,6-di-O-benzyl-2-deoxy-α-D-glucopyranosyl-(1→6)-1-O-(1,2-dimyristoyl-sn-glyceryl-phosphonato)-2,3,4,5-tetra-O-benzyl-D-myo-inositol **(15)**

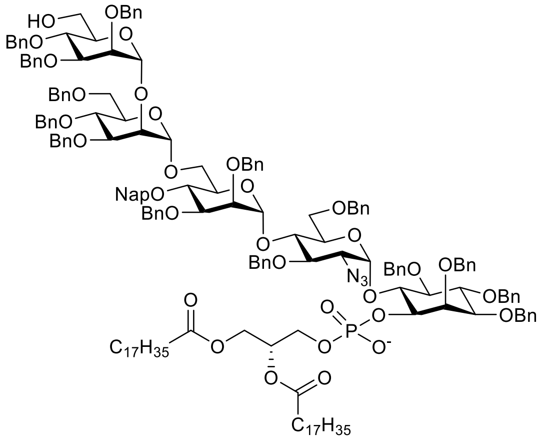

The pentasaccharide **14** (98 mg, 0.041 mmol) and H-phosphonate **7** (161 mg, 0.203 mmol) were dissolved and co-evaporated with anhydrous pyridine (3 x 3 mL) and dried under high vacuum overnight. The reaction mixture was dissolved in anhydrous pyridine (2 mL) at room temperature and pivaloyl chloride (20 μL, 0.163 mmol) was added. The resulting reaction mixture was stirred at room temperature for 3 h. Iodine (36.3 mg, 0.143 mmol) in a mixture of pyridine/water (19:1, 0.2 mL) was added to oxidize P (III) to P (V). The reaction mixture was further stirred for 2 h at room temperature to the complete the oxidation. The reaction mixture was diluted with $CHCl_3$ and washed with aqueous $Na_2S_2O_3$ solution to remove the excess iodine. The aqueous layer was further washed with $CHCl_3$ and the combined organic layer was dried over Na_2SO_4, filtered and concentrated under reduced pressure to give the crude residue, which was subjected to flash column with Et_3N-deactivated silica gel to give **10** (70 mg, 55 %) as a syrup. R_f = 0.25 ($CHCl_3$/MeOH = 20: 1). ^1H NMR (400 MHz, $CDCl_3$) δ 12.29 (s, 1H, NH, Et_3NH^+), 7.33 (d, *J* = 7.4 Hz, 2H), 7.29 – 6.91 (m, 73H), 5.80 (bs, 1H, anomeric), 5.19 – 5.10 (m, 3H, 2 x anomeric, C<u>H</u>), 4.95 (d, *J* = 11.9 Hz, 1H, CH_2- Bn), 4.91 – 4.63 (m, 11H), 4.63 – 4.19 (m, 21H), 4.19 – 3.90 (m, 9H), 3.90 – 3.26 (m, 16H), 3.23 (d, *J* = 10.4 Hz, 1H, $CH_{2a'}$), 3.07 (dd, *J* = 10.2, 3.7 Hz, 1H, CH_2a), 2.93 – 2.79 (dq, 6H, CH_2- Et_3NH^+), 2.16 (q, *J* = 7.3, 6.6 Hz, 4H, CH_2- lipid), 1.46 (q, *J* = 7.2 Hz, 4H, 2xCH_2- Lipid), 1.28 – 1.06 (m, 62H, lipid, CH_3-Et_3NH^+), 0.97 (d, *J* = 4.4 Hz, 21H), 0.80 (t, *J* = 6.6 Hz, 6H, CH_3- lipid). ^{13}C NMR (101 MHz, $CDCl_3$) δ 172.4, 172.1, 138.7, 137.6, 137.6, 137.4, 137.4, 137.3, 137.0, 136.9, 135.5, 132.3, 131.8, 127.5, 127.4, 127.4, 127.3, 127.2, 127.2, 127.2, 127.1, 127.0, 127.0, 126.9, 126.9, 126.8, 126.8, 126.8, 126.7, 126.7, 126.6, 126.6, 126.6, 126.5, 126.4, 126.4, 126.3, 126.2, 126.2, 126.1, 125.8, 124.9, 124.6, 99.9 (anomeric), 98.5 (anomeric), 98.3 (anomeric), 95.5 (anomeric), 80.7, 79.9, 78.7, 78.3, 75.2, 74.7, 74.0, 73.8, 73.5, 73.1, 72.7, 72.1, 71.7, 71.3, 71.2, 71.1, 71.0, 70.9, 70.6, 69.4, 69.4, 68.5, 67.9, 67.7, 65.0, 62.7, 61.9, 61.7, 61.1, 49.8, 44.3, 33.3, 33.1, 30.9, 28.8, 28.7, 28.7, 28.5, 28.4, 28.1, 23.9, 23.8, 21.7, 17.1, 17.0 (CH3- TIPS), 13.1 (CH_3- Lipid), 11.0 (-CH- TIPS), 7.4 (CH_2 lipid).

In the second step, the lipidated pentasaccharide **14a** (70 mg, 0.023 mmol) was passed through a column of Amberlite IR120 ion exchange resin (Na^+ form) using $CHCl_3$/MeOH (1:1) as eluent. The eluate was collected and concentrated to dryness. The obtained residue was then dissolved in CH_3CN (2.5 mL) with a trace of H_2O (100 μL). $Sc(OTf)_3$ (55.6 mg, 0.113 mmol) was added to the reaction mixture and the resulting mixture was heated to 45 ºC and stirred overnight. The reaction mixture was cooled down to room temperature and few drops of pyridine was added to quench any excess $Sc(OTf)_3$. The reaction mixture was evaporated to dryness and subjected to flash column chromatography with Et_3N-deactivated silica gel to afford **15** (45 mg, 67.7 %) as a syrup. R_f = 0.24 ($CHCl_3$/MeOH = 20 : 1); ^1H NMR (400 MHz, Chloroform-*d*) δ 12.25 (s, 2H, NH- $^+$N<u>H</u>Et_3), 7.90 (d, *J* = 34.5 Hz, 1H), 7.61 – 6.80 (m, 94H),

5.86 (bs, 1H, anomeric), 5.51 – 5.33 (m, 1H, anomeric), 5.23 – 4.92 (m, 3H, anomeric, CH_2-Bn), 4.88 – 4.46 (m, 10H), 4.44 – 4.17 (m, 9H), 4.19 – 3.90 (m, 5H), 3.89-3.61 (m, 4H), 3.59-301 (m, 6H), 2.74 (p, J = 7.3, 6.8 Hz, 14H, CH_2- $^+$NHEt$_3$), 2.30 – 2.05 (m, 4H, -CH_2- lipid), 1.59 – 1.40 (m, 6H, -CH_2- lipid), 1.17 (d, J = 8.2 Hz, 62H, lipid)), 1.05 (t, J = 7.3 Hz, 20H, CH_3- $^+$NHEt$_3$), 0.81 (t, J = 6.6 Hz, 6H, CH3- lipid). ^{13}C NMR (101 MHz, $CDCl_3$:MeOD=10:1) δ 173.50 (C=O ester), 173.10 (C=O ester), 139.73, 138.64, 138.49, 138.43, 138.35, 138.03, 137.95, 136.52, 133.30, 132.80, 128.55, 128.47, 128.41, 128.34, 128.29, 128.22, 128.18, 128.07, 128.01, 127.96, 127.90, 127.84, 127.77, 127.69, 127.65, 127.49, 127.38, 127.24, 127.11, 126.87, 125.98, 125.65, 100.90 (anomeric), 99.53 (anomeric), 99.35 (anomeric), 96.59 (anomeric), 81.73, 80.98, 79.77, 79.30, 75.00, 74.85, 74.55, 74.19, 73.76, 73.15, 72.78, 72.30, 72.17, 72.05, 71.90, 71.69, 70.53, 69.54, 68.99, 68.73, 66.05, 63.73, 62.97, 62.79, 62.17, 50.85, 45.40, 34.31, 34.11, 31.96, 29.83, 29.76, 29.71, 29.58, 29.41, 29.20, 24.87, 22.73, 18.10, 14.17 (CH_3- Lipid), 12.03, 8.44 (CH_2 lipid). ^{31}P NMR (162 MHz, $CDCl_3$:MeOD =10:1) δ -1.63. ESI-HRMS (*m/z*): $[M+Na+NH_4]^{2+}$ calcd 1491.2763; obsd, 1491.2341; $[M-H]^-$ cald 2939.5188, obsd, 2939.3640.

(6 O-(2-(N-(tert-butoxycarbonyl)-S-(tert-butyl)-L-cysteinyl)aminoethyl phosphonato)-2,3,4-Tri-O-benzyl-α-D-mannopyranosyl-(1→2)-3,4,6-tri-O-benzyl-α-D-mannopyranosyl-(1→6)-2,3-di-O-benzyl-4-O-(2-naphthyl)methyl-α-D-mannopyranosyl-(1→4)-2-azido-3,6-di-O-benzyl-2-deoxy-α-D-glucopyranosyl-(1→6)-1-O-(1,2-dimyristoyl-sn-glyceryl-phosphonato)-2,3,4,5-tetra-O-benzyl-D-myo-inositol **(16)**

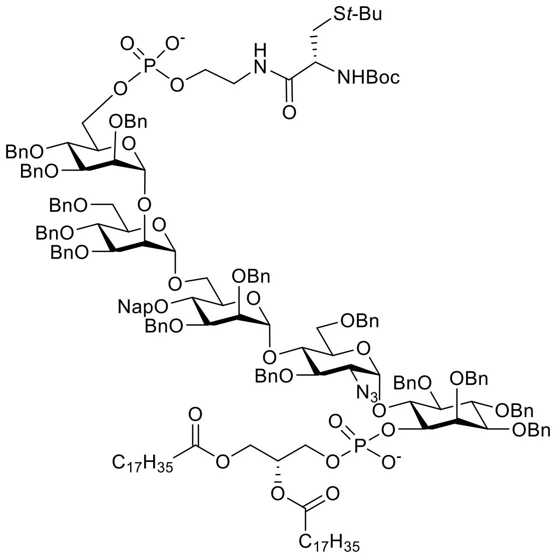

The bilipidated pseudopentasaccharide **15** (40 mg, 0.014 mmol) and *H*-phosphonate **10**[10] (39 mg, 0.102 mmol) were dissolved and co-evaporated with anhydrous pyridine (3 x 3 mL) and dried under high vacuum overnight. The reaction mixture was dissolved in anhydrous pyridine (2 mL) at room temperature and pivaloyl chloride (13 µL, 0.102 mmol) was added. The resulting reaction mixture was stirred at room temperature for 5 h. The progress of the reaction was monitored by using TLC analysis. Iodine (26 mg, 0.102 mmol) in a mixture of pyridine/water (19:1, 0.2 mL) was added to oxidize P (III) to P (V). The reaction mixture was further stirred for 2 h at room temperature to complete the oxidation, diluted with $CHCl_3$, washed with aqueous $Na_2S_2O_3$ solution to remove the excess iodine. The aqueous layer was further washed with $CHCl_3$ and the combined organic layer was dried over Na_2SO_4, filtered and concentrated under reduced pressure to give the crude residue, which was subjected to flash

column with Et_3N-deactivated silica gel to give **16** (32 mg, 70 %) as a syrup. R_f = 0.35 ($CHCl_3$/MeOH = 10: 1); ^{1}H NMR (700 MHz, $CDCl_3$:MeOD=10:1) δ 7.73 (d, J = 7.8 Hz, 1H, NH), 7.66 – 7.57 (m, 3H, Ar), 7.47 – 6.76 (m, 73H, Ar), 5.85 (d, J= 3.7 Hz, 1H, anomeric), 5.43 (d, J = 8.0 Hz, 1H), 5.23-5.13 (m, 3H, CH_2 –Bn, anomeric), 4.99 (dd, J = 43.0, 10.7 Hz, 3H), 4.87 – 4.78 (m, 4H), 4.74 – 4.50 (m, 10H), 4.45 – 4.30 (m, 14H), 4.28 – 4.21 (m, 5H), 4.18 – 4.08 (m, 2H), 4.05-4.01 (m, 6H), 3.98 – 3.91 (m, 3H), 3.89 – 3.74 (m, 4H), 3.74 – 3.63 (m, 6H), 3.61 – 3.41 (m, 8H), 3.28 – 3.13 (m, 4H), 3.07 (d, J = 7.1 Hz, 1H), 2.8 (d, J = 5.4 Hz, 2H), 2.64 (q, J = 7.2 Hz, 1H CH_2) 2.47 (dq, J = 8.3, 7.9, 4.8 Hz, 12H, CH_2- TEA), 2.31 (q, J = 7.2 Hz, 2H, CH_2), 2.17 (dt, J = 7.8, 5.9 Hz, 4H, CH_2) 1.49 – 1.45 (m, 4H), 1.34 (s, 9H, Boc), 1.17 (m, 65H, Lipid, tBu), 0.81 (t, J = 7.0 Hz, 6H). ^{13}C NMR (151 MHz, $CDCl_3$:MeOD, 10:1) δ 173.32 (C=O amide), 172.95 (C=O ester), 170.17 (C=O ester), 155.15, 139.78, 138.84, 138.70, 138.63, 138.46, 138.36, 138.12, 137.99, 136.56, 133.27, 132.72, 128.50, 128.35, 128.31, 128.26, 128.20, 128.11, 128.04, 127.97, 127.88, 127.79, 127.74, 127.69, 127.63, 127.56, 127.47, 127.40, 127.31, 127.18, 127.08, 126.89, 125.91, 125.54, 125.39, 125.23, 100.81 (anomeric), 99.53 (anomeric), 99.04 (anomeric), 96.27 (anomeric), 81.82, 81.71, 80.98, 79.98, 79.86, 79.30, 76.34, 76.10, 75.93, 75.62, 74.83, 74.61, 74.49, 74.23, 73.84, 73.74, 73.10, 72.87, 72.30, 72.25, 72.17, 71.99, 71.83, 71.66, 70.57, 69.33, 68.96, 68.84, 66.30, 64.04, 63.78, 63.62, 63.10, 62.80, 54.13, 42.33 (CH_2- TEA), 40.28 (CH_2), 34.27, 34.06, 31.89, 31.66, 30.91 (CH_2), 30.1 ((CH_3- tBu), 29.63, 29.50, 29.33, 29.13, 27.57 (CH_3- tBu) 28.33, 24.88, 24.84, 22.65, 13.38 (2xCH_3- Lipid). ^{31}P NMR (162 MHz, $CDCl_3$:MeOD = 10:1) δ 1.73, -1.61. ESI-HRMS (m/z): $[M+2K]^{2+}$ calcd: 1701.2959, obsd: 1701.2985; $[M-2H]^{2-}$ calcd: 1661.3255, obsd: 1661.3218.

(6-(N-(S-S-mercaptoethanol-L-Cysteinyl)aminoethanol-phosphonato)-α-D-mannopyranosyl-(1→2)-α-D-mannopyranosyl-(1→6)-α-D-mannopyranosyl-(1→4)-2-amino-2-deoxy-α-D-glucopyranosyl-(1→6)-1-O-(1,2-dimyristoyl-sn-glycerol-phosphate)-D-myo-inositol **(**GPI**2)**

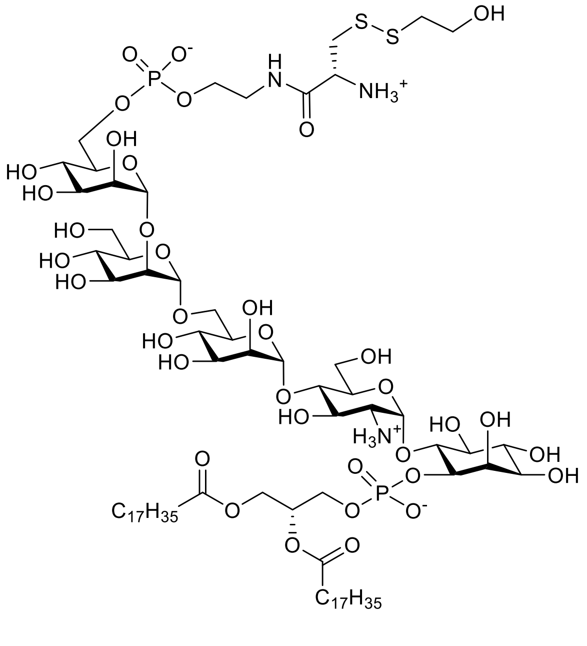

The bisphosphorylated pseudopentasaccharide **16** (10.0 mg, 0.003 mmol) was dissolved in mixture of THF/MeOH/H_2O (2:1:1, 5 mL) with formic acid (4 % v/v, 200 µL) at room temperature and Pd/C (256 mg, 0.24 mmol, 10% Pd content) was added. Hydrogen gas was bubbled through the solution for 15 min and the reaction mixture was stirred under an atmosphere of hydrogen gas for additional 16 h. The palladium was removed by filtration through a pad of celite and the solvents were removed under reduced pressure to give 4 mg of the crude debenzylated product. The dibenzylated compound was confirmed by mass spectrometry. The crude material (4 mg) was then dissolved at 0 °C in a mixture of trifluoroacetic acid and anisole (11 ml, 10:1 v/v) and stirred for 5 min. Then $Hg(TFA)_2$ (9.1 mg, 21 µmol) was added to the reaction mixture and stirred for an additional 30 min. the volatiles were removed under high vacuum at 0 °C. The resulting solid residue was dissolved in an AcOH/water mixture (15 mL, 7:3 v/v) and mercaptoethanol (400 µL, 5.7 mmol) was added at room temperature. The product was purified using Sephadex LH-20 column using $CHCl_3$/MeOH/H_2O (10:10:3) for elution to give 1.8 mg (50 % yield) of GPI**2**. The product was characterized by NMR, however due to the low solubility, spectra were only possible in a mixture of solvents. The given integral values are approximated. ^1H NMR (400 MHz, Rf = 0.3 (DCM/MeOH, 5:1), ^1H NMR (600 MHz, $CDCl_3$/MeOD/D_2O, 3:3:0.1) δ 8.31 (s, 1H), 5.49 (s, 1H, anomeric), 5.28-5.21 (m, 1H, anomeric), 5.18-5.05 (m 4 H), 5.09 (m, 2H), 4.94 (s, 0H), 4.23 – 3.50 (m, 13H), 3.43 (d, J = 10.1 Hz, 1H), 2.73 – 2.63 (m, 5H), 2.31 (m, 4H, CH_2-cysteine), 2.21 – 1.92 (m, 19H), 6.65-1.45 (m, 6H, lipid), 1.54 – 1.44 (m, 4H), 1.26 (m, 50H, lipid), 1.14 (m, 2H, lipid), 0.88 – 0.84 (m, 6H, 2 xCH_3 lipid). ^{31}P NMR (243 MHz, $CDCl_3$/MeOD/D_2O, 3:3:0.1) δ -2.44, -3.08, ESI-MS (*m/z*): $C_{76}H_{143}N_3O_{37}P_2S_2$ $[M+H]^+$ cald 1739.860, $[M+2NH_4]^+$ obsd 1773.6470.

LC-MS Characterization of the GPIs

GPI2 Samples were dissolved in a mixture of methanol/chloroform/water (3:3:1) and analyzed in Phenomenex Jupiter C4 column (50 x 2.1 mm), gradient 5 – 70% ACN with 0.1% FA in water with 0.1% FA in 30 min on a Waters Aquity H-Class UPLC, flow rate 0.5 mL/min and mass spectra were obtained with Waters Xevo G2-XS mass spectrometer in ES^- mode, 0.8 kV capillary voltage.

a

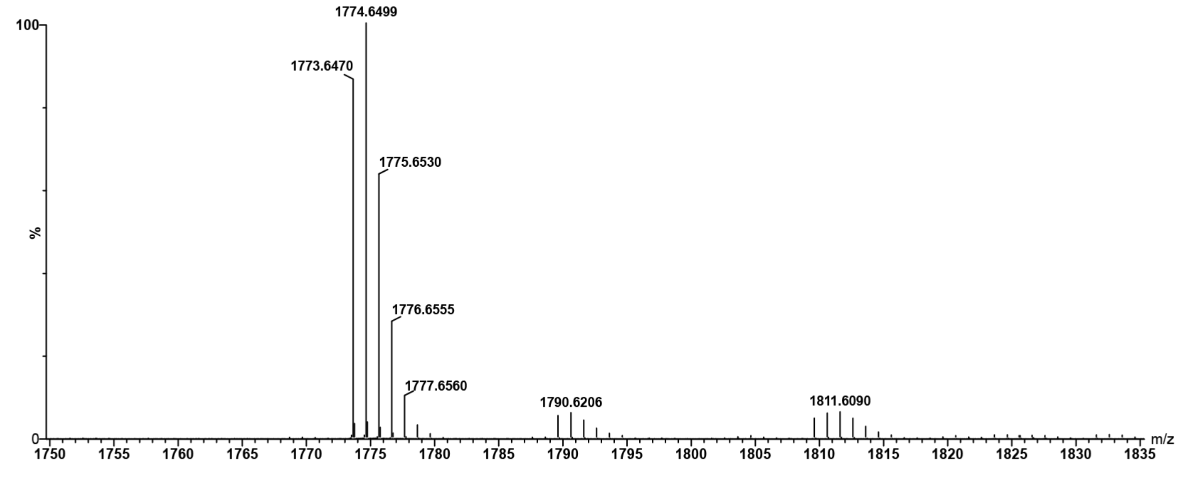

b

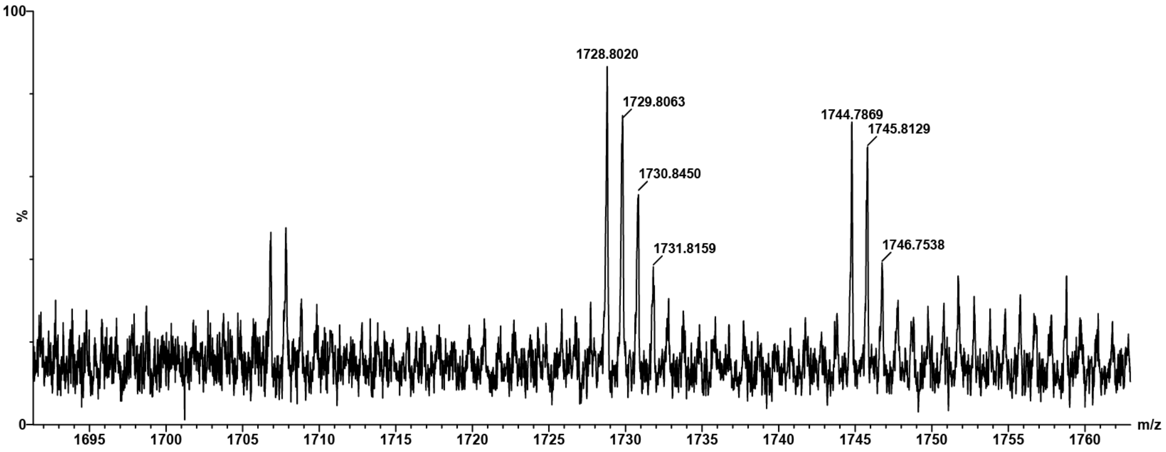

Figure **S17.** ESI-MS spectra of **GPI2**, $[M+H]^+_{calc}$=1739.83 Da. a) spectrum acquired in ES^+ mode, $[M+H]^+_{obs}$=1773.6470 m/z, which corresponds to a double ammonium adduct, b) spectrum acquired in ES^- mode, $[M-H]^-_{obs}$=1728.8020 Da and 1744.7869 Da, both of which cannot clearly assigned to the product (Δ-9 and +7 Da).

The **GPI1** Sample was dissolved in 20 % ACN and analyzed in YMC C_8 ProPack column (50 x 3 mm), gradient 5 – 70% ACN with 0.1% FA in water with 0.1% FA in 30 min on Waters Aquity H-Class UPLC, flow rate 0.5 mL/min and mass spectra were obtained with Waters Xevo G2-XS mass spectrometer in ES^- mode, 0.8 kV capillary voltage.

a

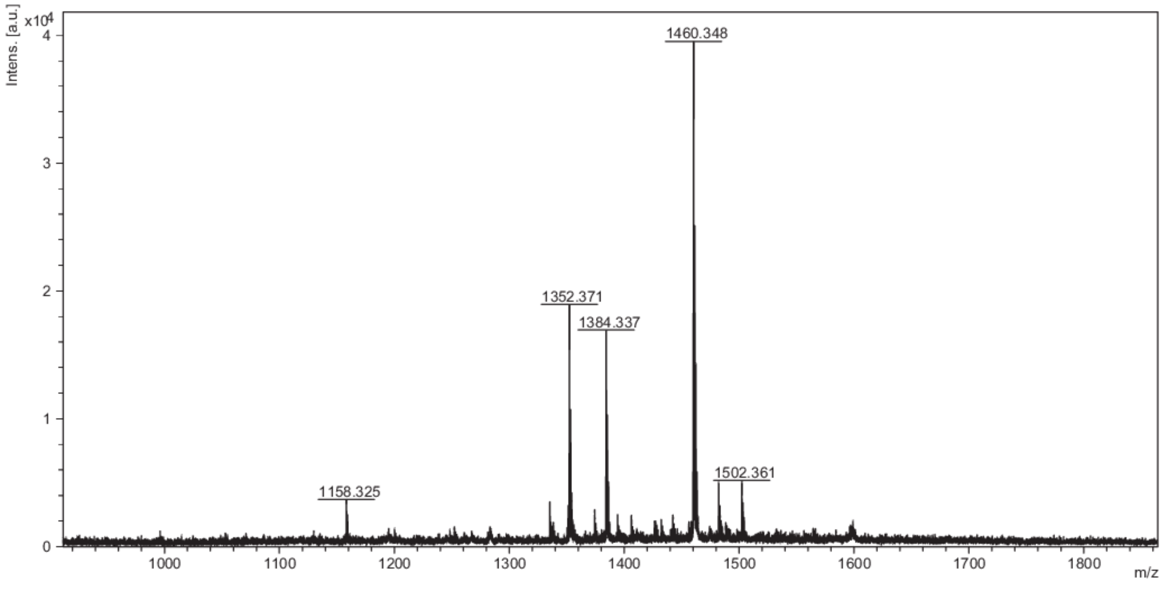

b

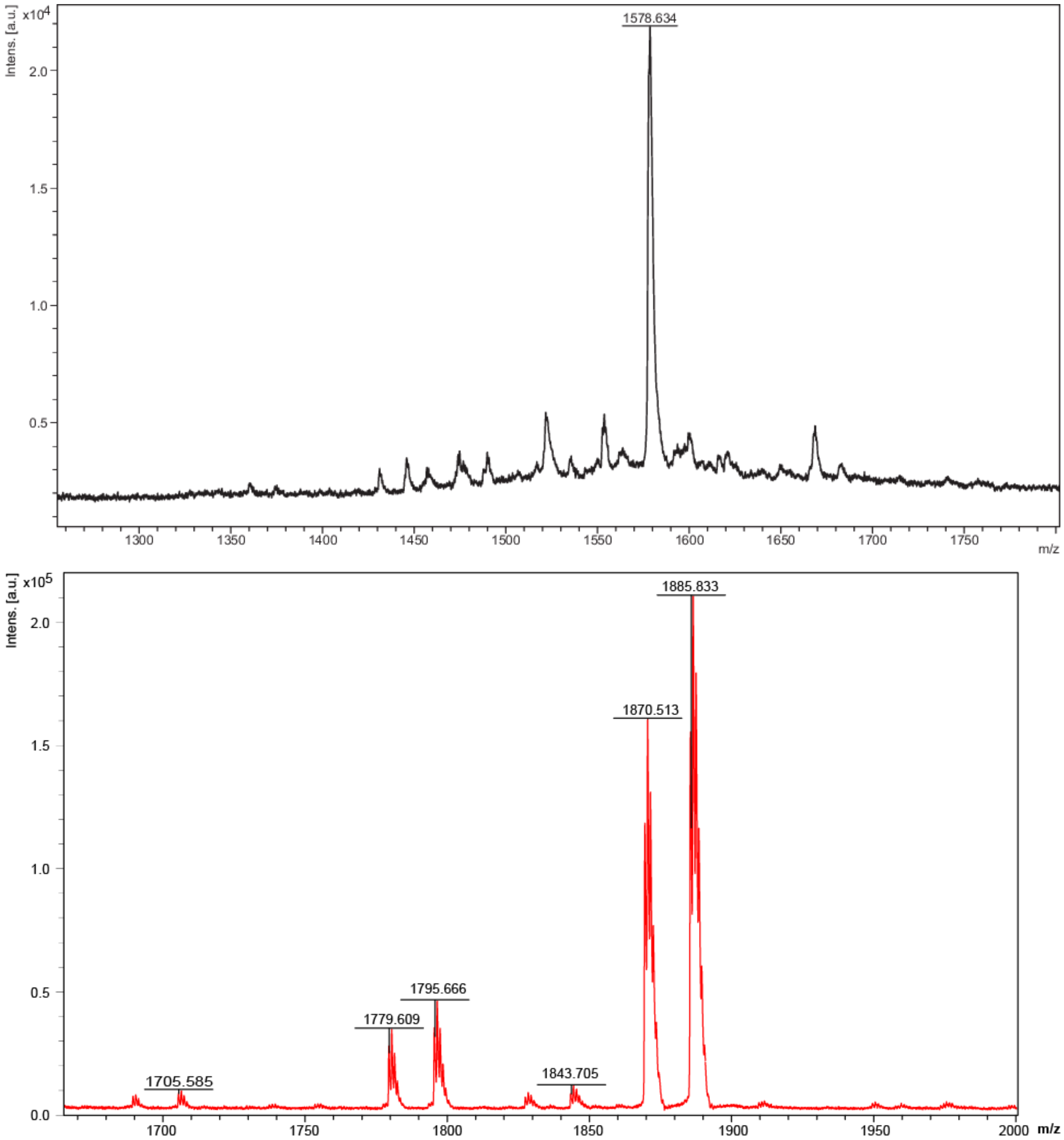

Figure **S18.** MALDI-TOF-MS analysis of GPI molecules. a) MALDI-TOF mass spectrum of GPI**,** $[M\text{-}H]^{-}_{obs}$=1460.348 Da, $[M\text{-}H]^{-}_{calc}$=1458.5 Da, b) MALDI-TOF MS spectrum of **GPI1**, **[**M-H]$^{-}_{obs}$=1578.634 Da, **[**M-H]$^{-}$ calculated =1581.54 Da. C) MALDI-TOF MS spectrum of **GPI3**, **[**M-H]$^{-}_{obs}$=1885.833 Da, **[**M-H_2O]$^{-}$ calculated =1884.54 Da. All spectra were acquired in negative mode.

a

b

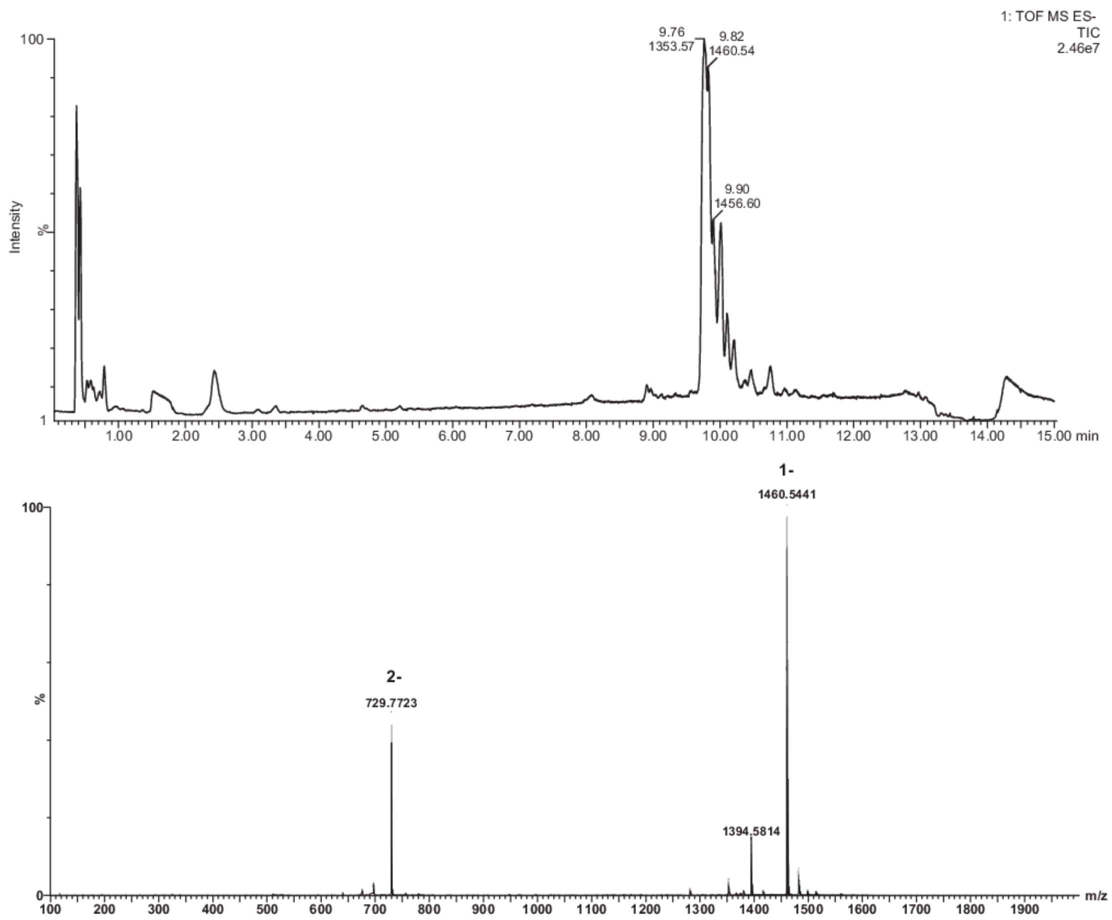

Figure **S19.** LC-ESI-MS analysis of **GPI1**. a) Total ion chromatogram (TIC) of **GPI1** analysis, b) mass spectrum acquired at t_R=9.8 min, $[M+H]^+_{obs}$=1460.5441 Da, $[M+H]^+_{calc}$=1460.534.

NMR Spectra

^{1}H NMR of tetramannoside **14** (400 MHz, $CDCl_3$),

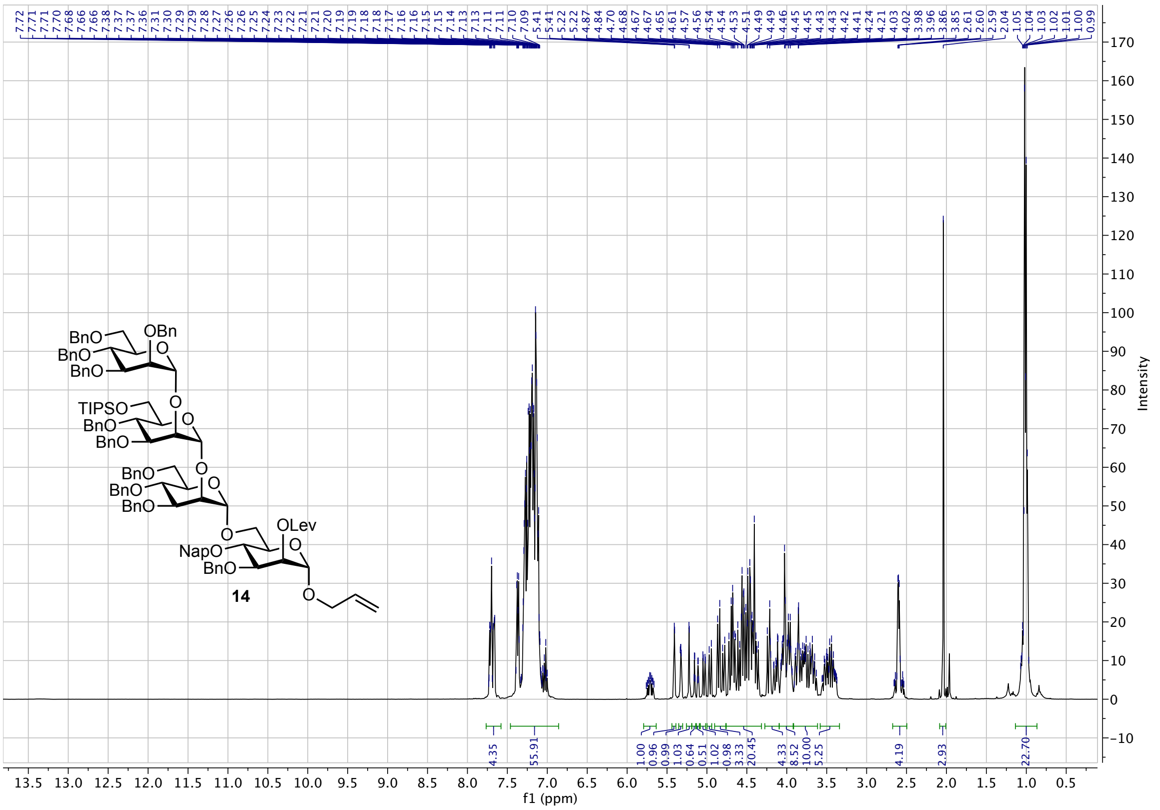

^{13}C NMR of tetrammanoside **14** (101 MHz, $CDCl_3$)

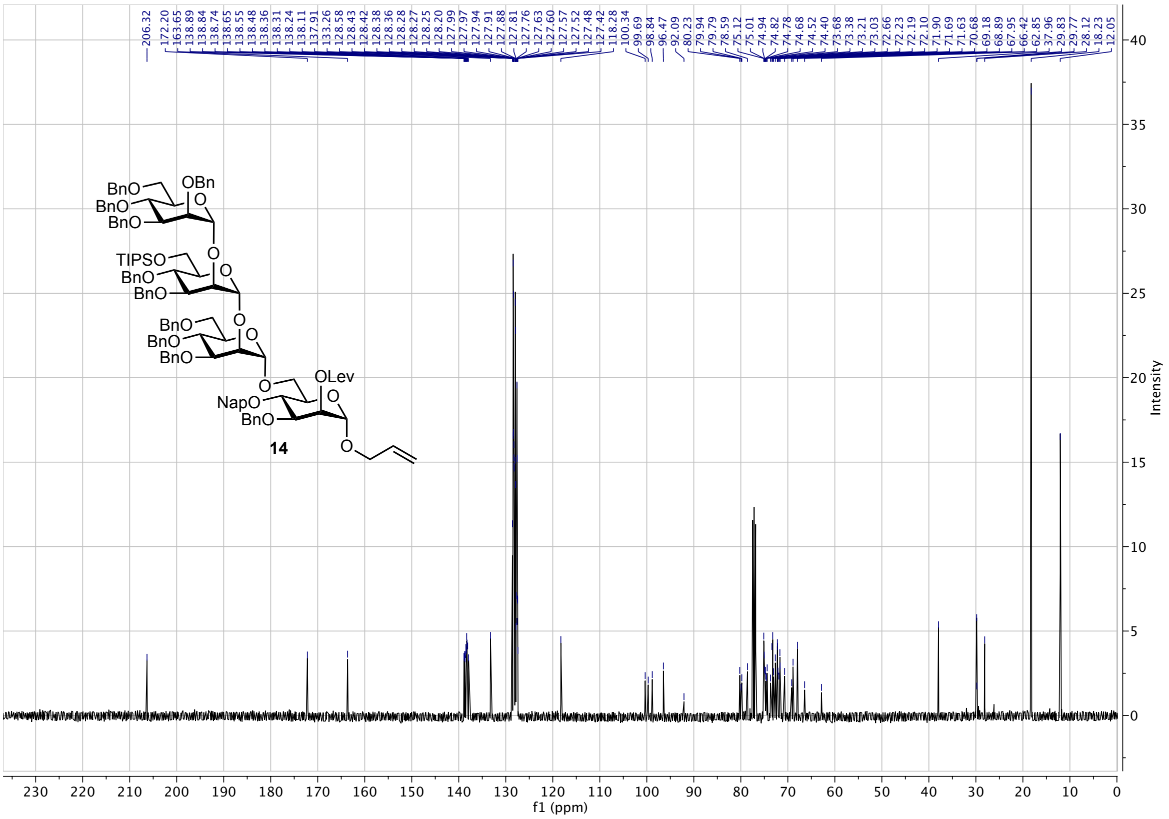

^{1}H NMR of imidate **15** (400 MHz, $CDCl_3$)

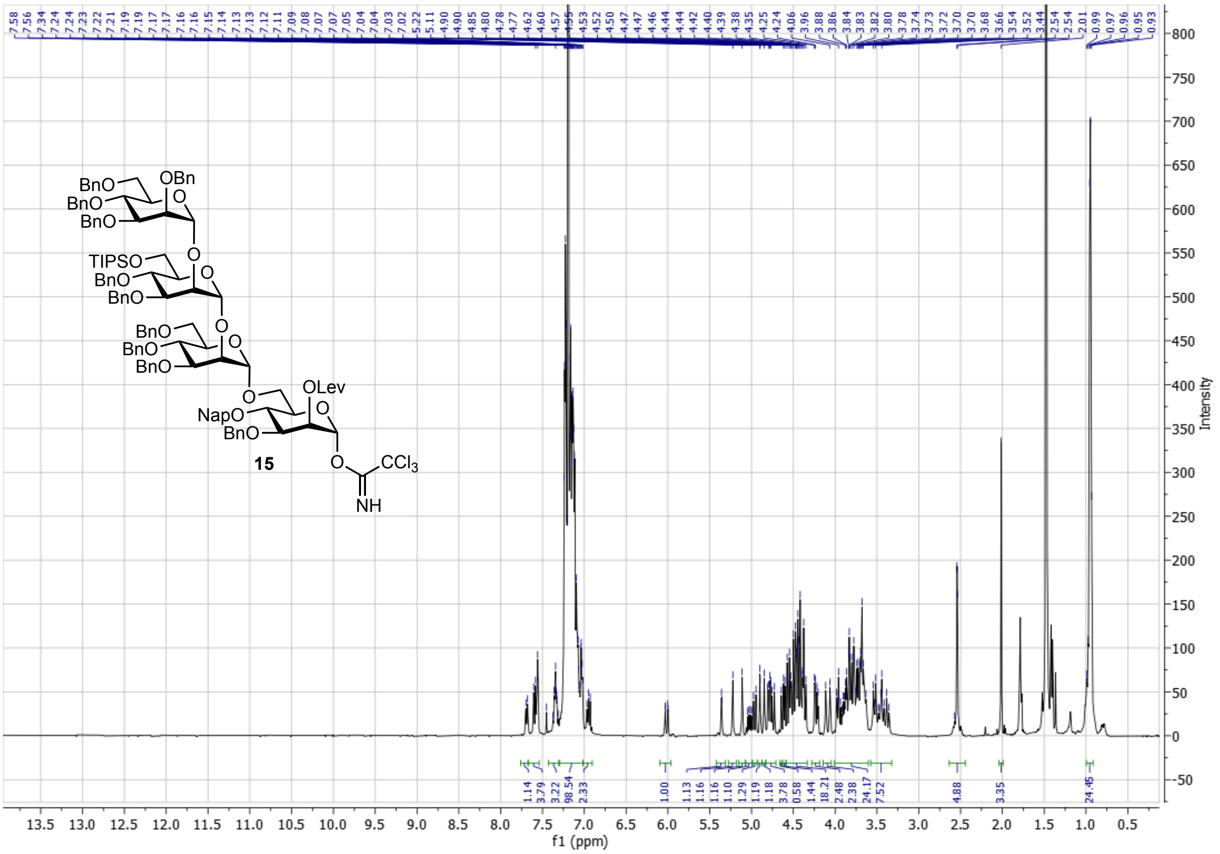

^{1}H NMR spectrum of *pseudo*-hexasaccharide **5** (400 MHz, $CDCl_3$)

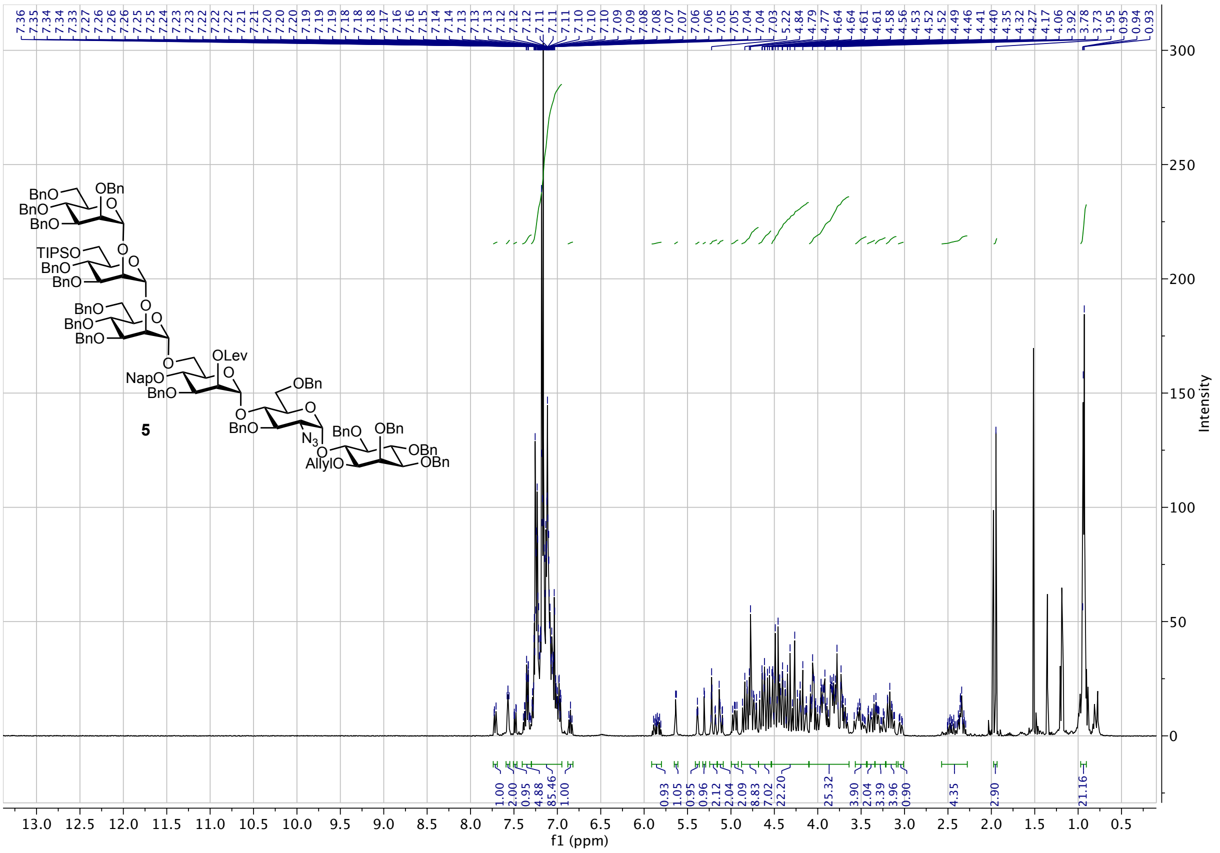

400 NMR coupled HSQC spectrum of *pseudo*-hexasaccharide **5** ($CDCl_3$)

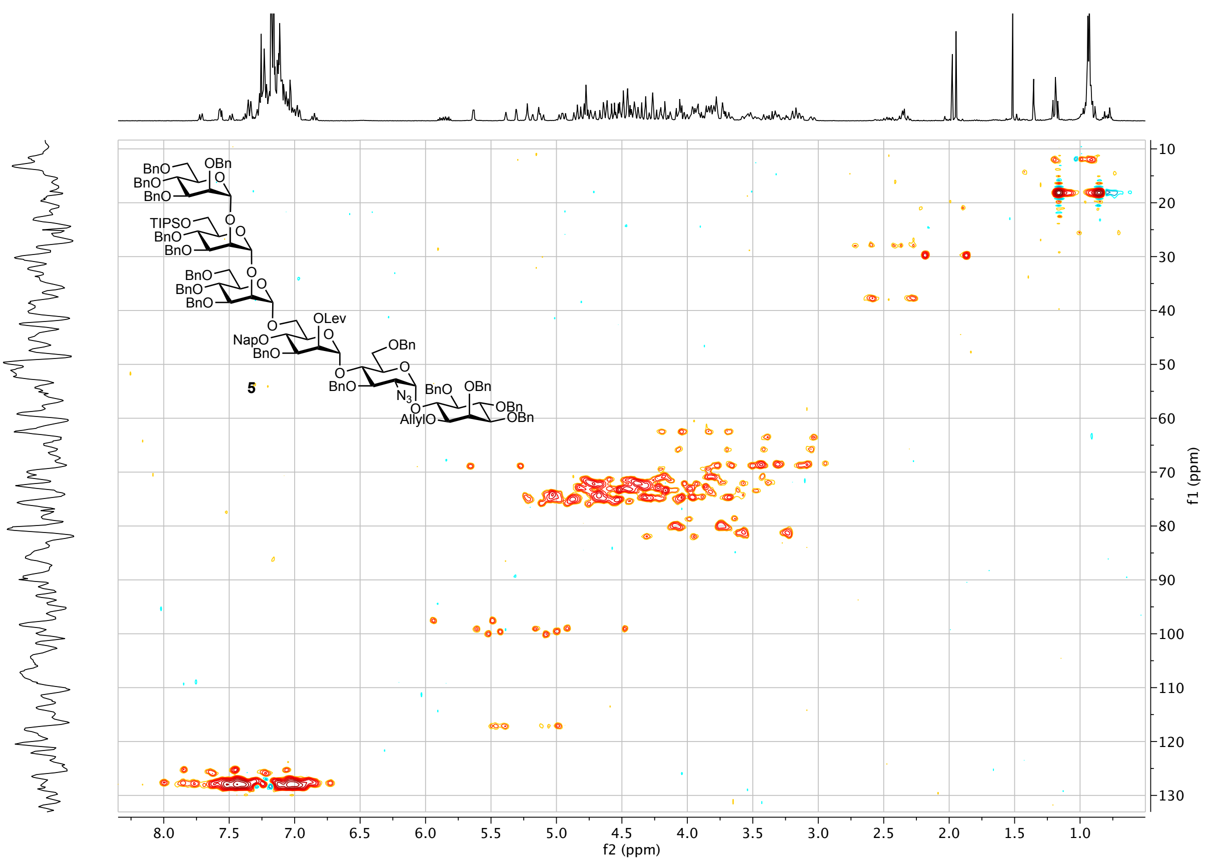

^{1}H NMR spectrum of *pseudo*-hexasaccharide **6** (400 MHz, $CDCl_3$)

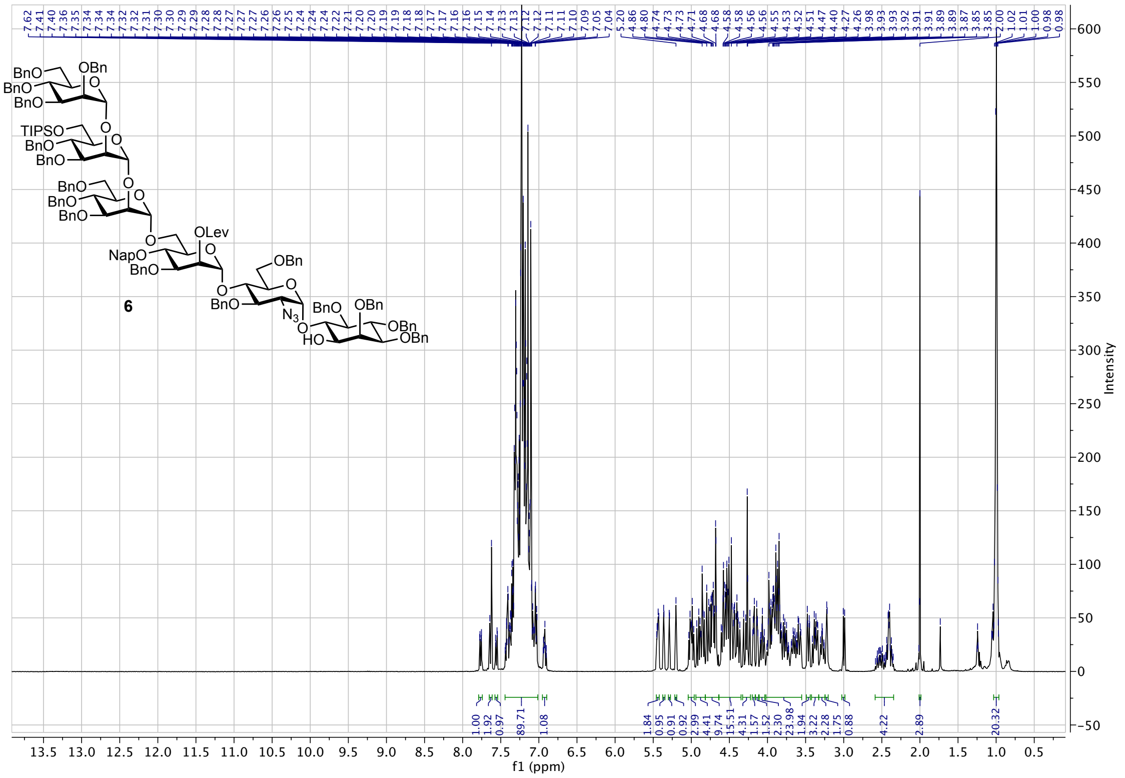

^{13}C NMR of *pseudo*-hexasaccharide **6** (101 MHz, $CDCl_3$)

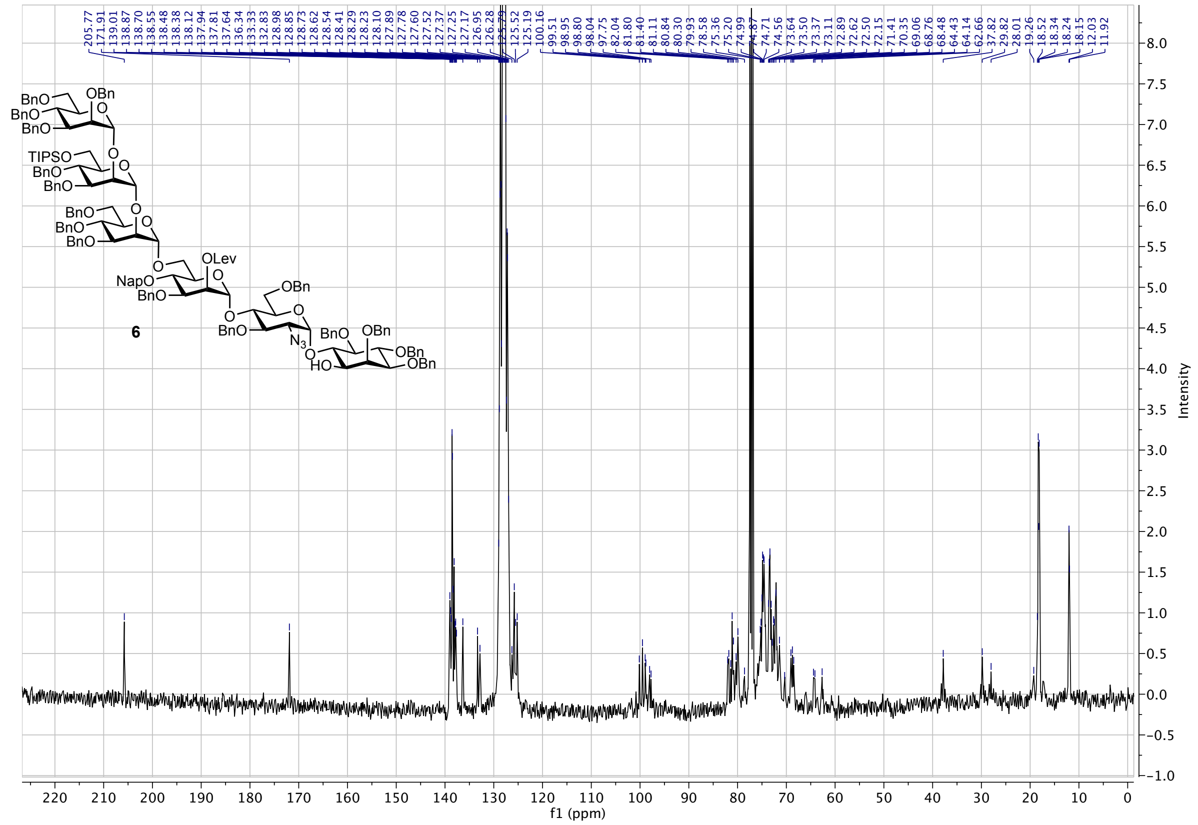

^{1H} NMR of H-phosphonate **7** (400 MHz, $CDCl_3$)

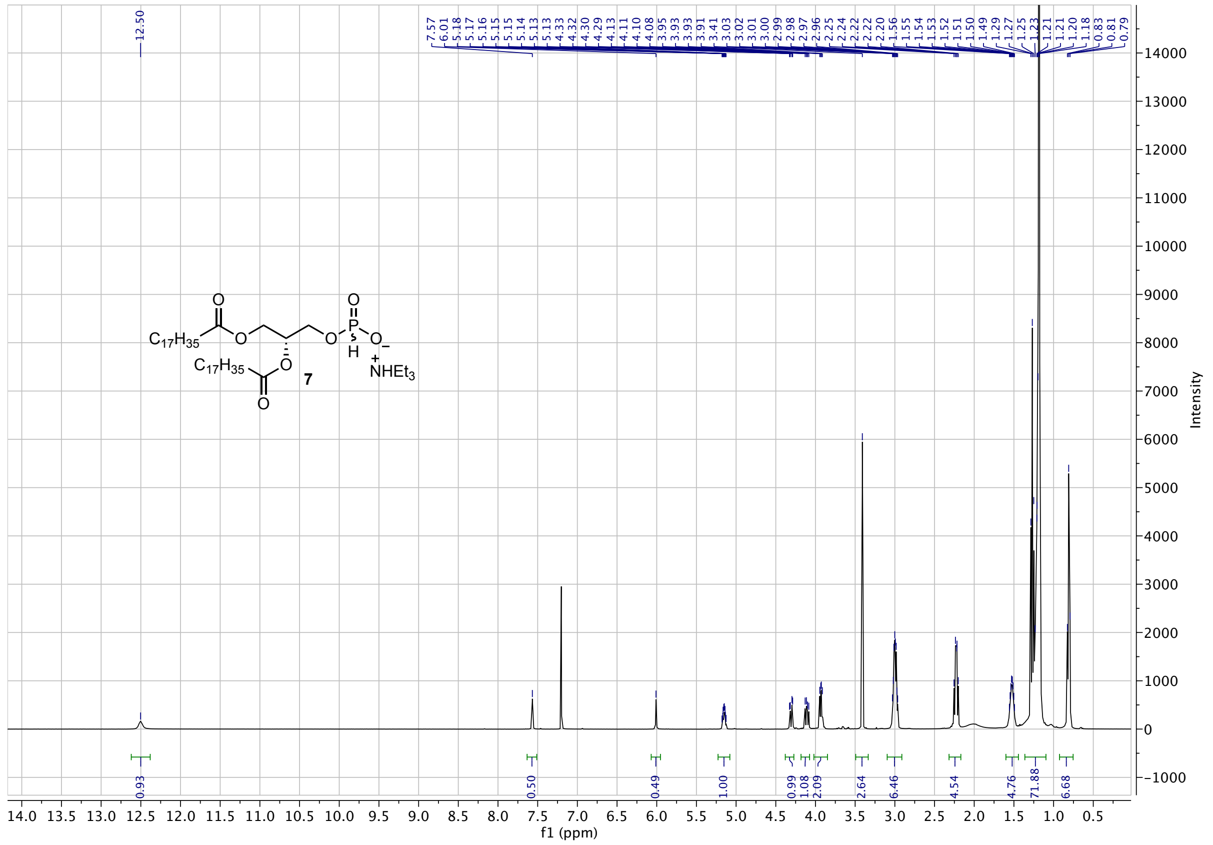

^{13}C NMR of H-phosphonate **7** (101 MHz, $CDCl_3$)

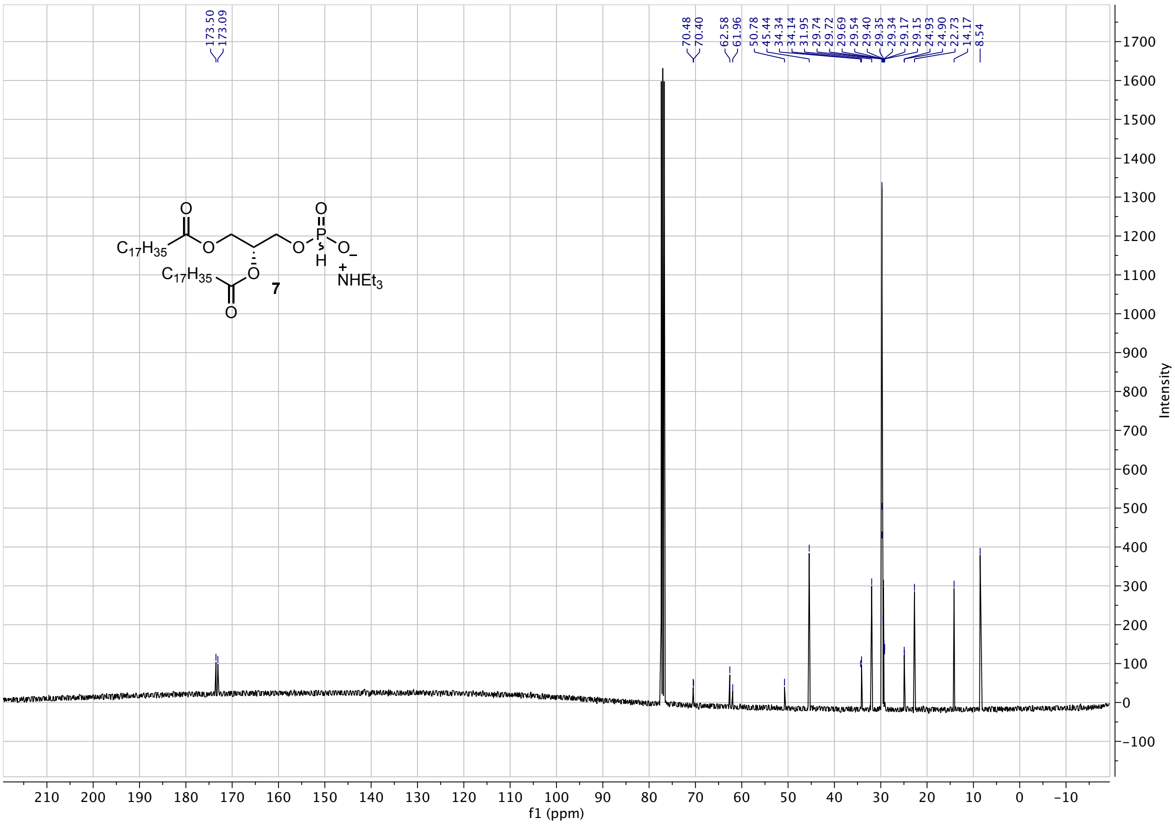

^{31}P NMR of H-phosphonate **7** (162 MHz, $CDCl_3$)

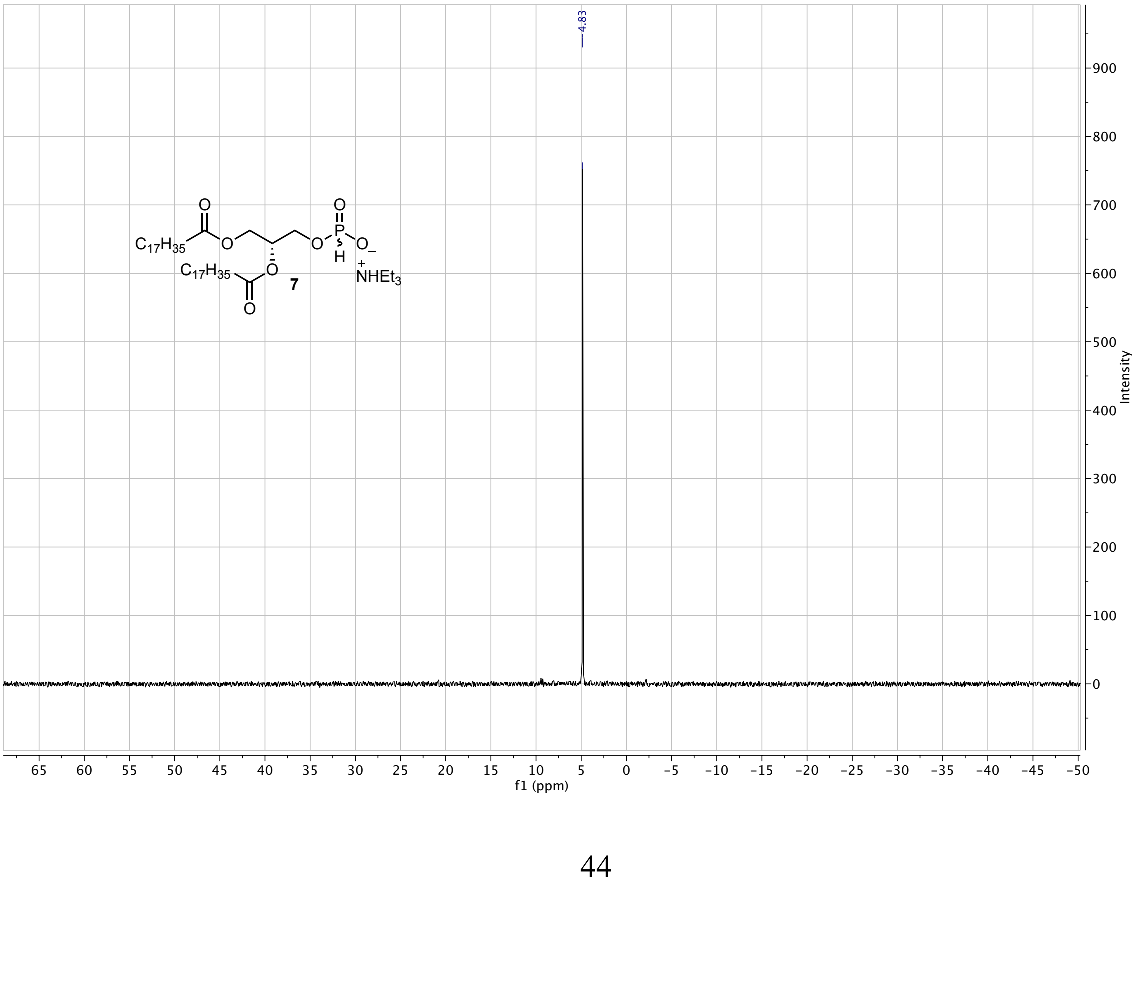

^{1}H NMR spectrum of lipidated *pseudo*-hexasaccharide **8** (400 MHz, $CDCl_3$)

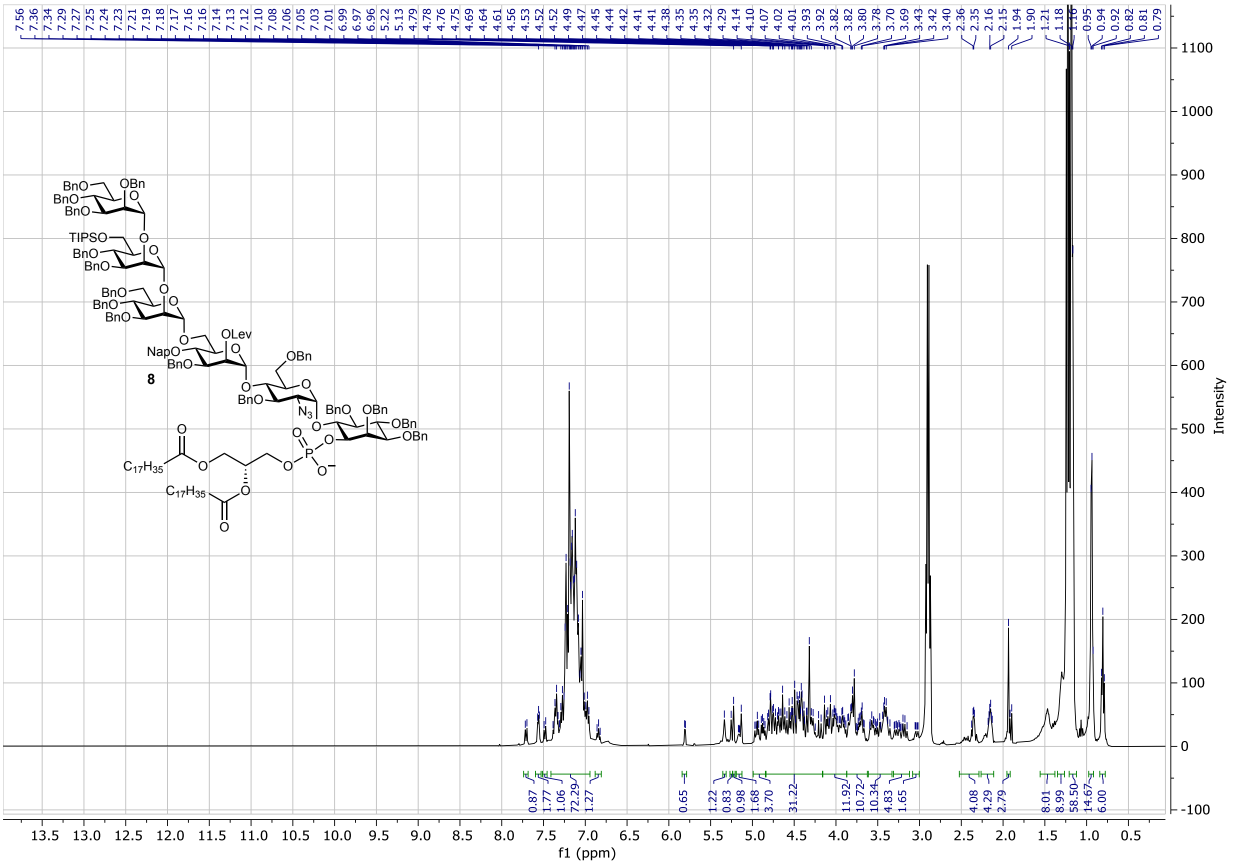

^{13}C NMR of spectrum of lipidated *pseudo*-hexasaccharide **8** (101 MHz, $CDCl_3$)

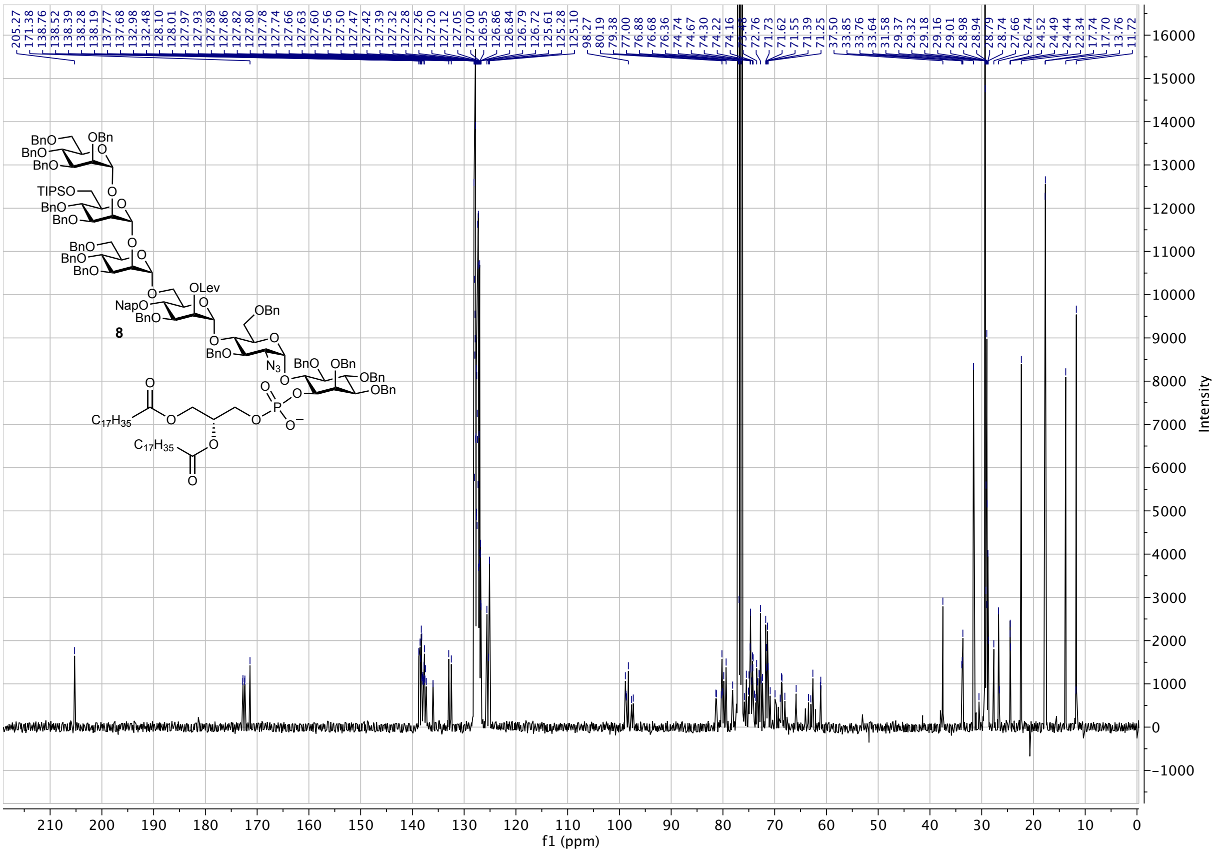

^{31}P NMR spectrum of lipidated *pseudo*-hexasaccharide **8** (162 MHz, $CDCl_3$)

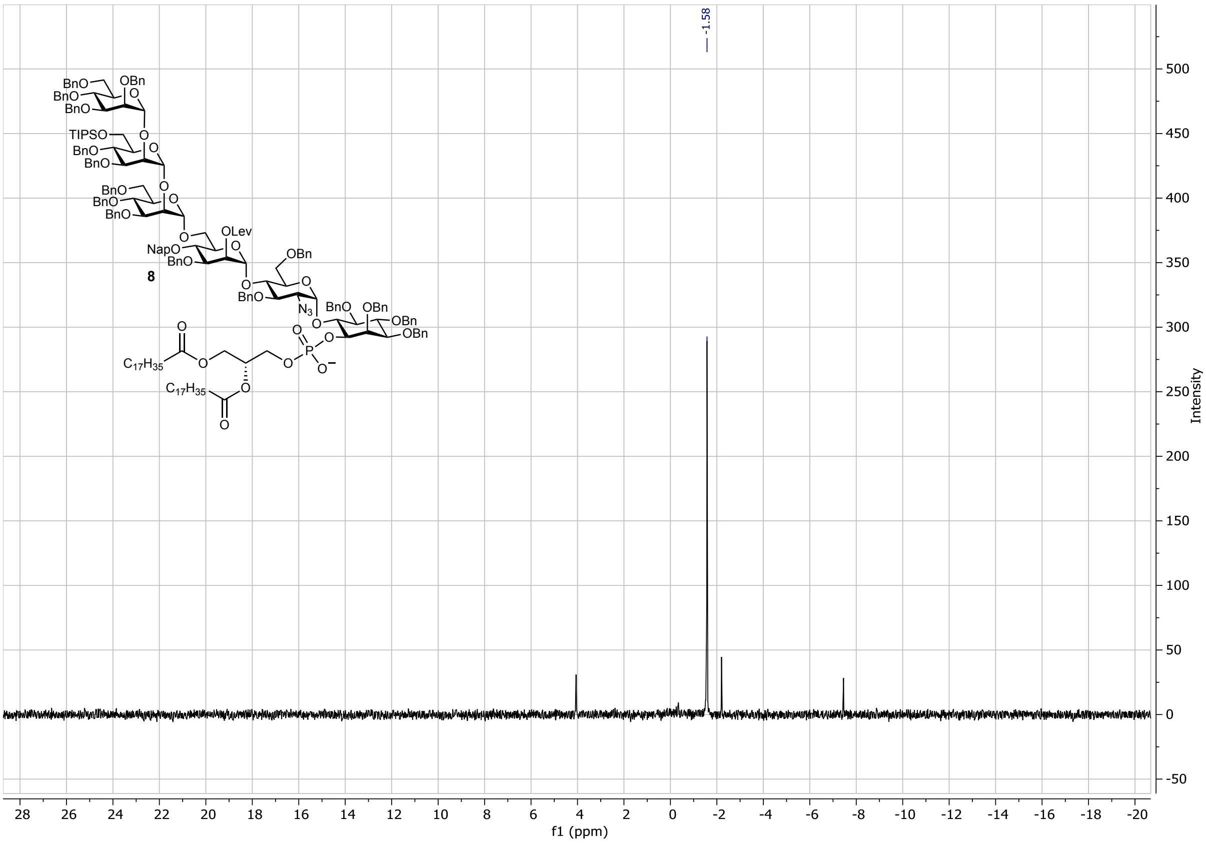

^{1}H NMR spectrum of bisphosphorylated *pseudo*-hexasaccharide **11**

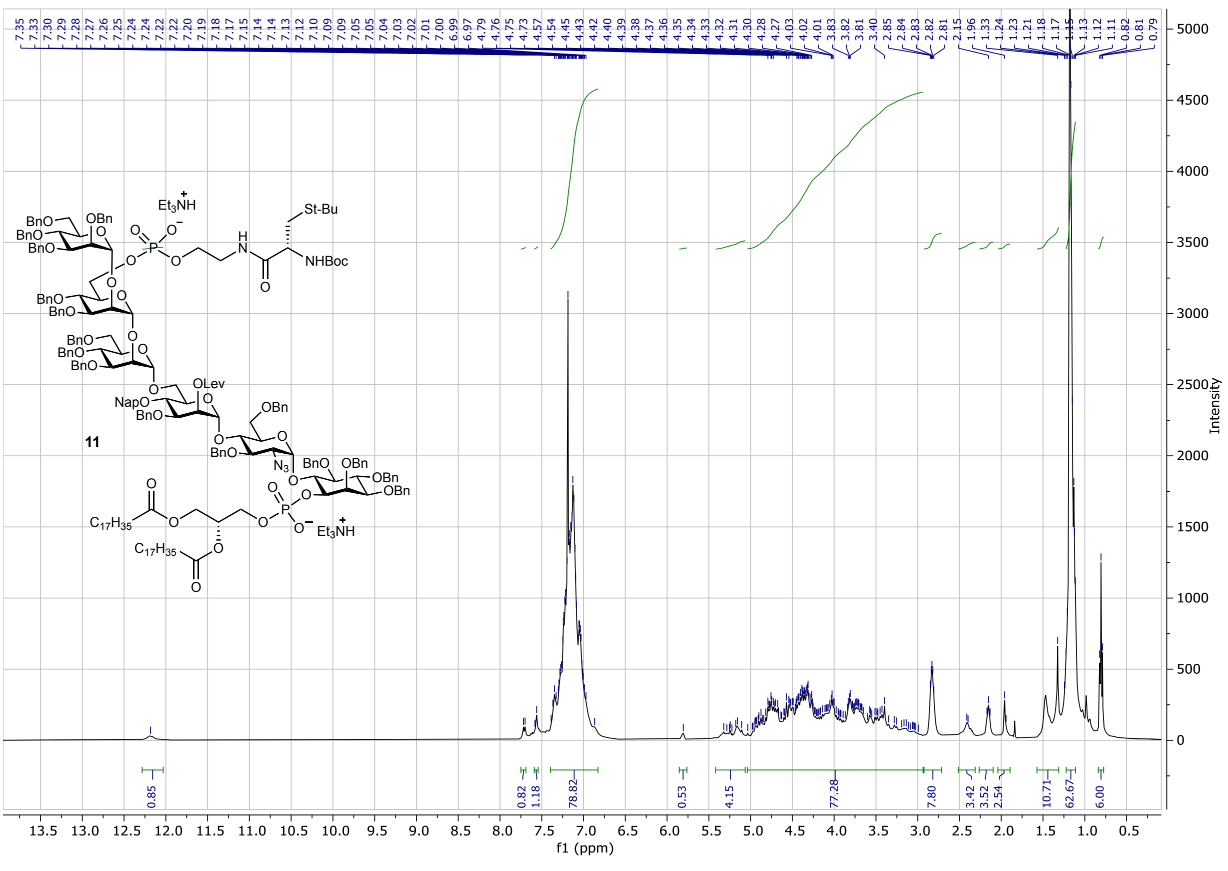

HSQC NMR spectrum of bisphosphorylated *pseudo*-hexasaccharide **11**

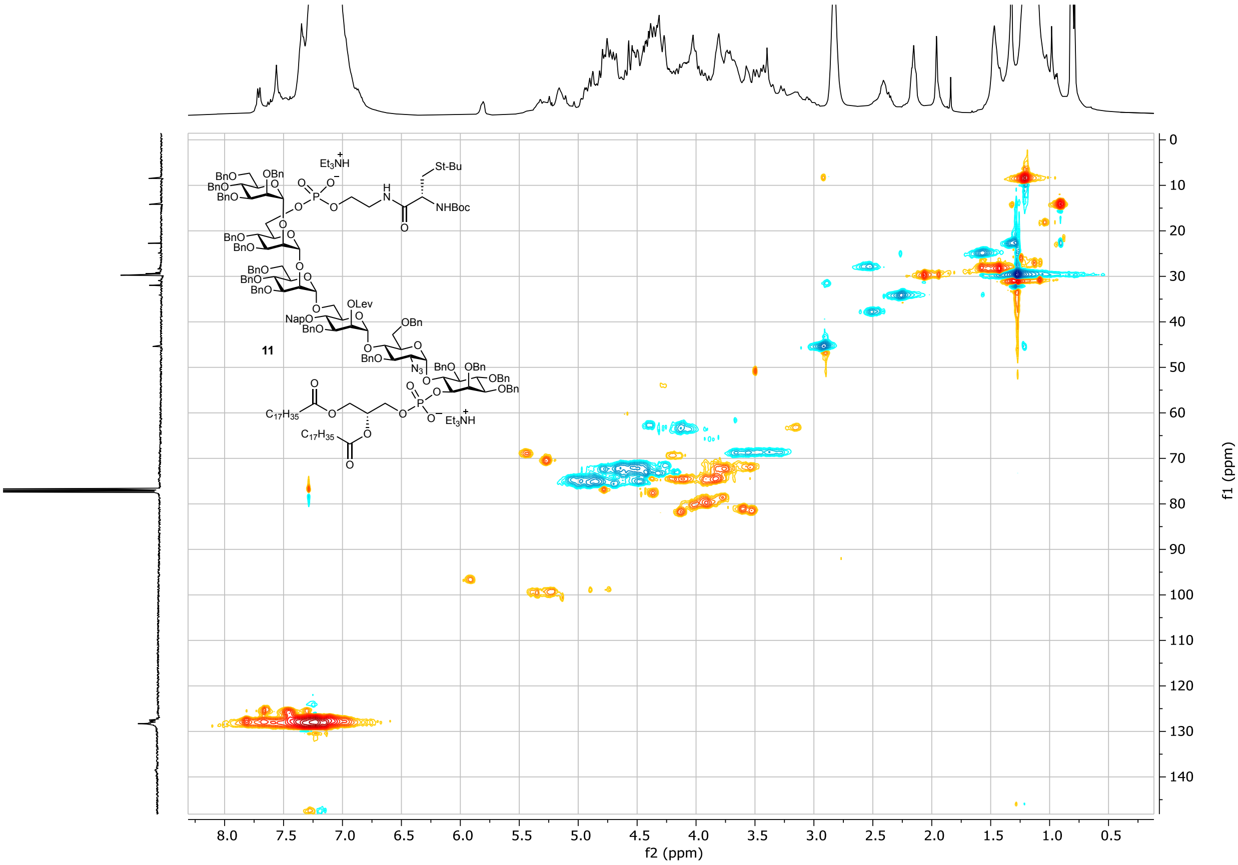

^{31}P NMR spectrum of bisphosphorylated *pseudo*-hexasaccharide **11**

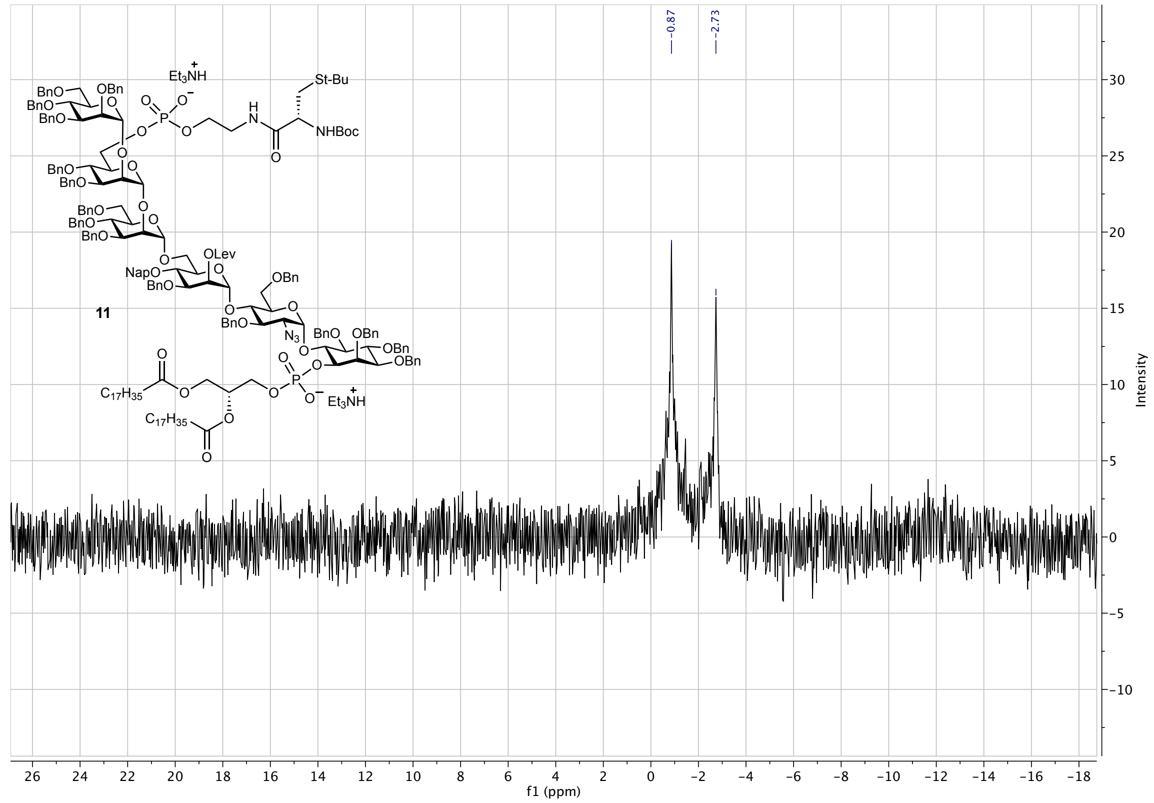

^{1}H NMR spectrum of GPI**3** (400 Mhz, $CDCl_3$/MeOD/D_2O, 10:10:1)

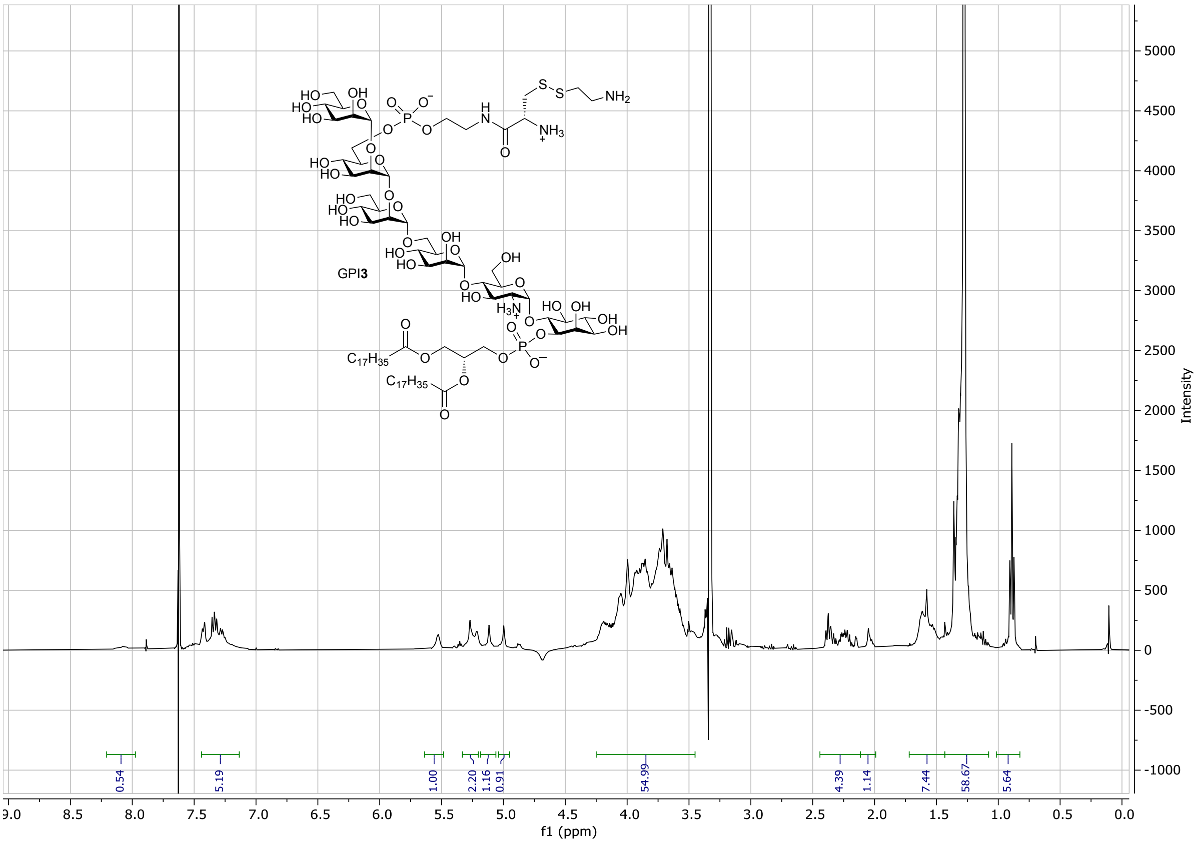

^{31}P NMR spectrum of **GPI3** (162 Mhz, $CDCl_3$/MeOD/D_2O, 10:10:1)

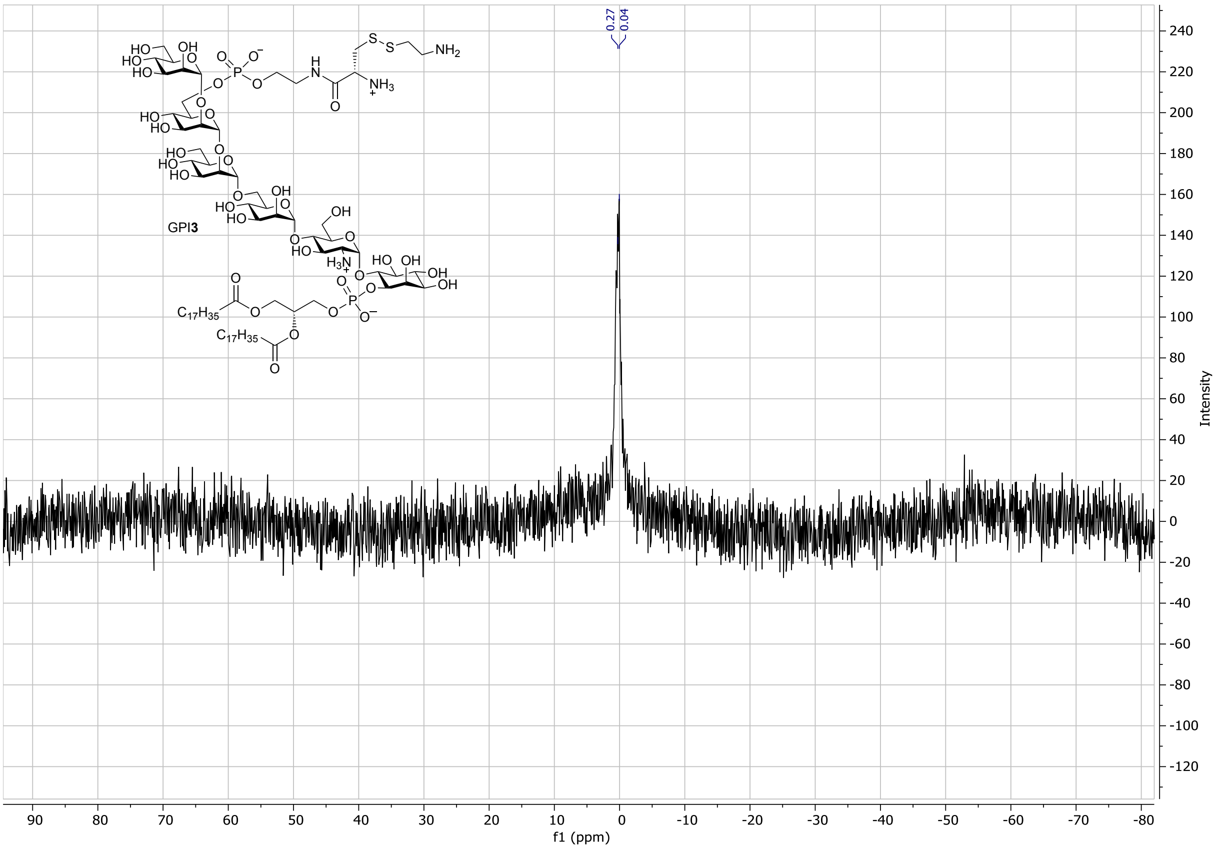

^{1}H NMR spectrum of *pseudo*-pentasaccharide **13** (400 MHz, $CDCl_3$)

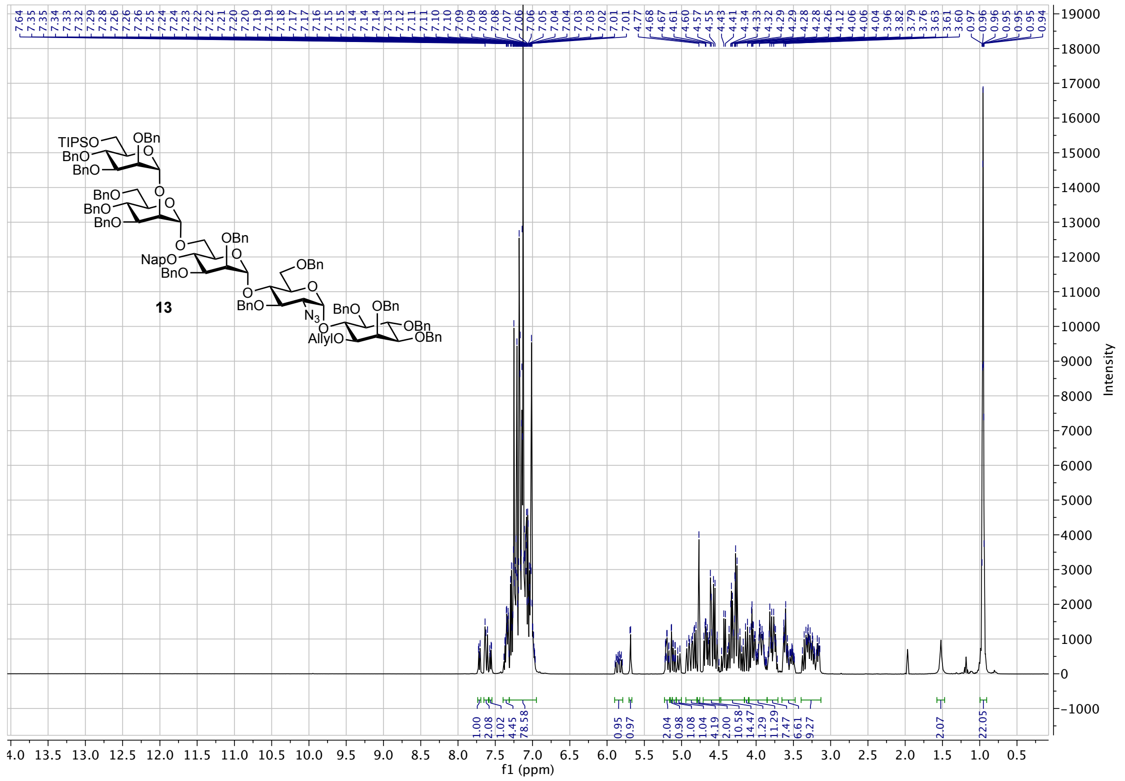

^{13}C NMR spectrum of *pseudo*-pentasaccharide **13** (101 MHz, $CDCl_3$)

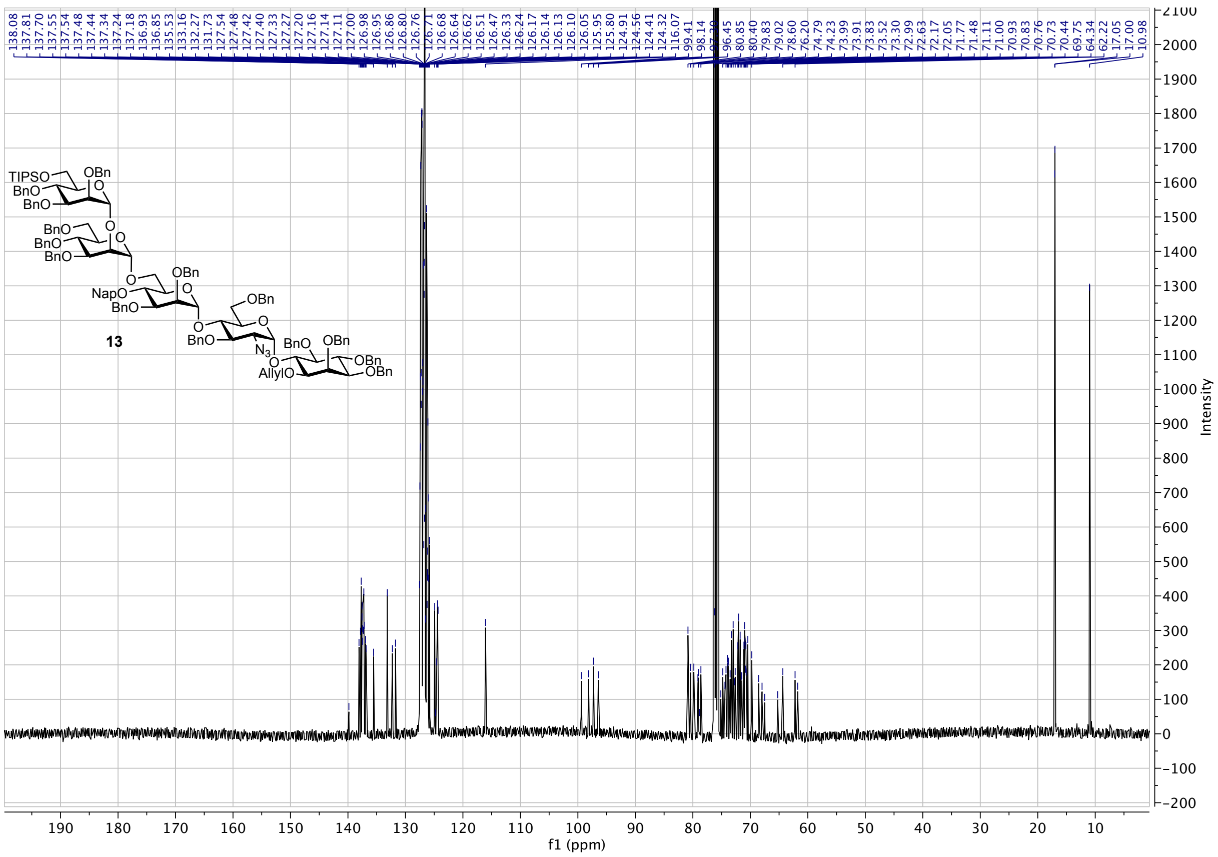

^{1}H NMR spectrum of *pseudo*-pentasaccharide **14** (400 MHz, $CDCl_3$)

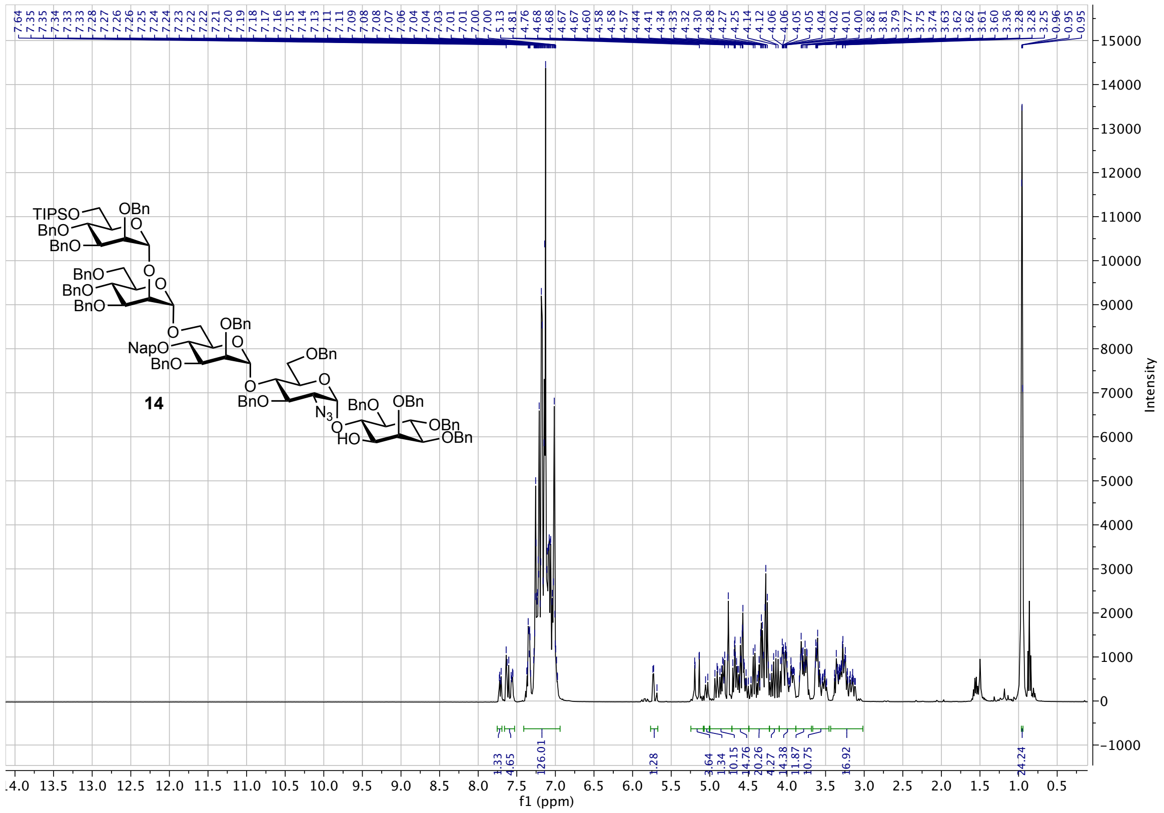

^{13}C NMR spectrum of *pseudo*-pentasaccharide **14** (101 MHz, $CDCl_3$)

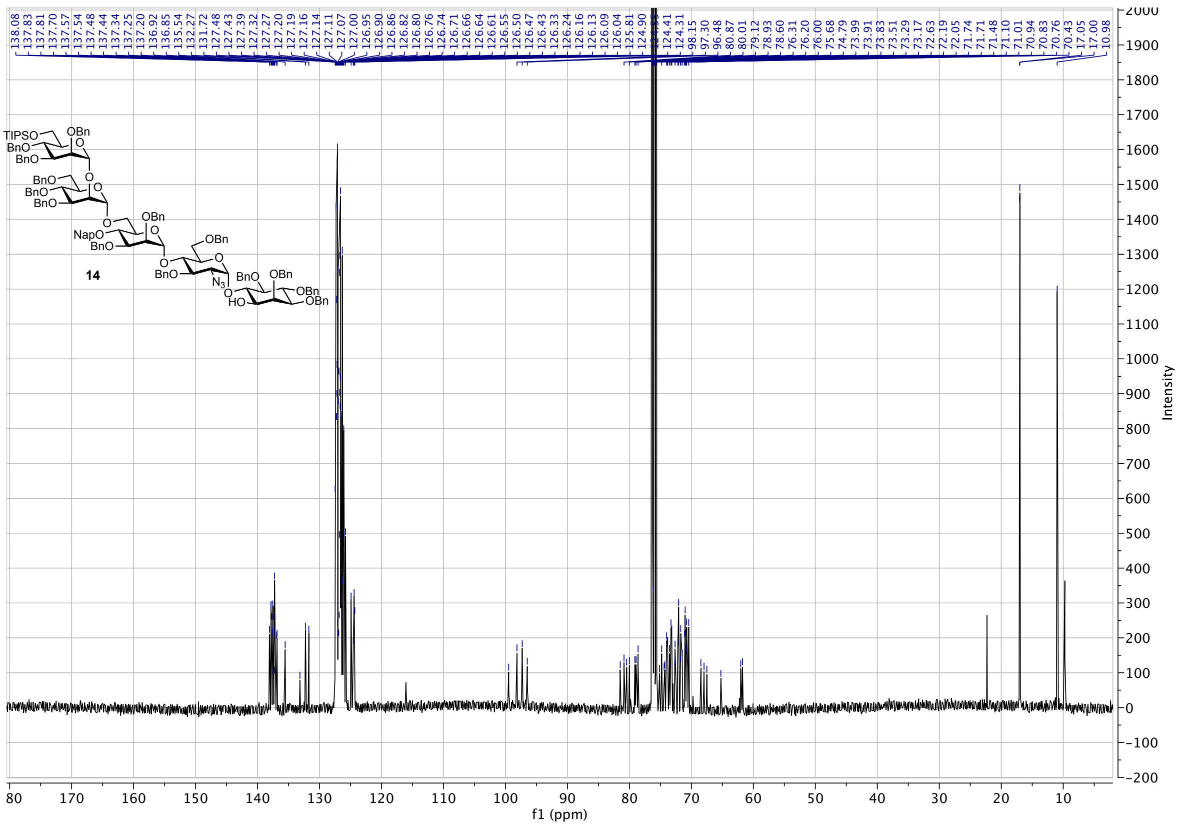

^{1}H NMR spectrum of lipidated *pseudo*-pentasaccharide **14a** (400 Mhz, $CDCl_3$/MeOD (10:1)

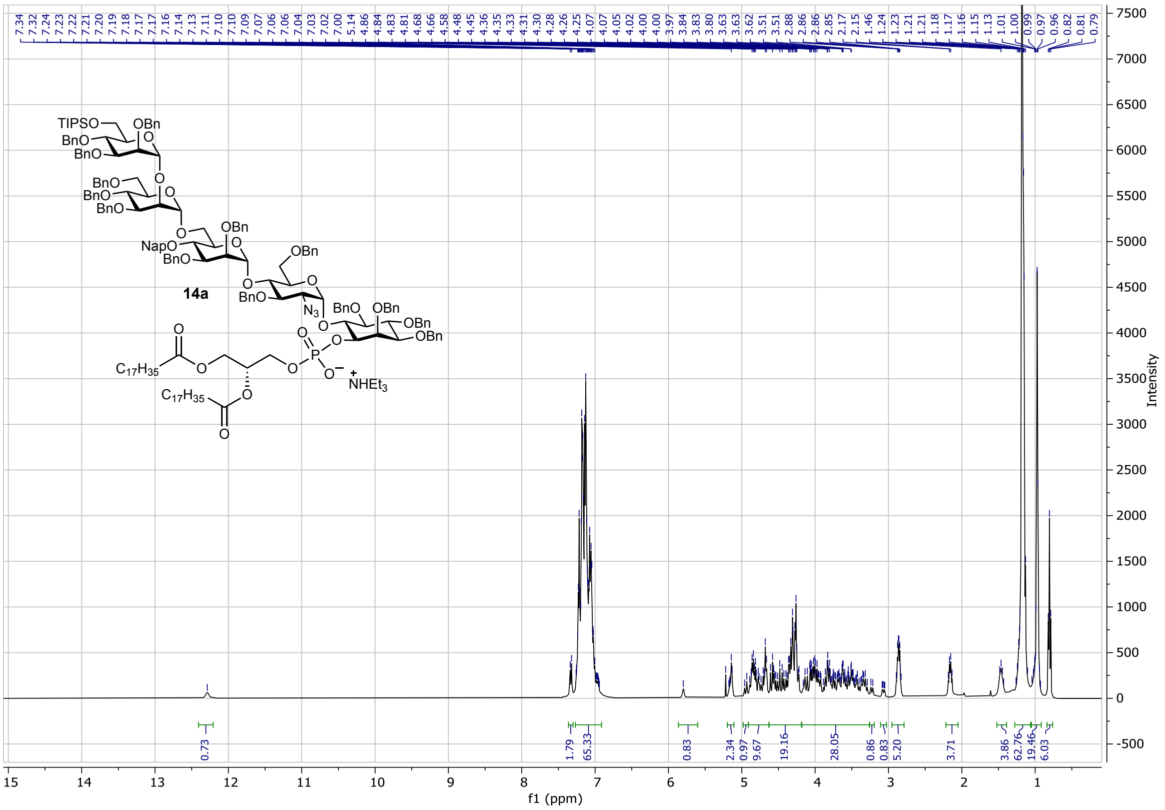

^{1}H NMR spectrum of lipidated *pseudo*-pentasaccharide **15** (400 Mhz, $CDCl_3$/MeOD (10:1)

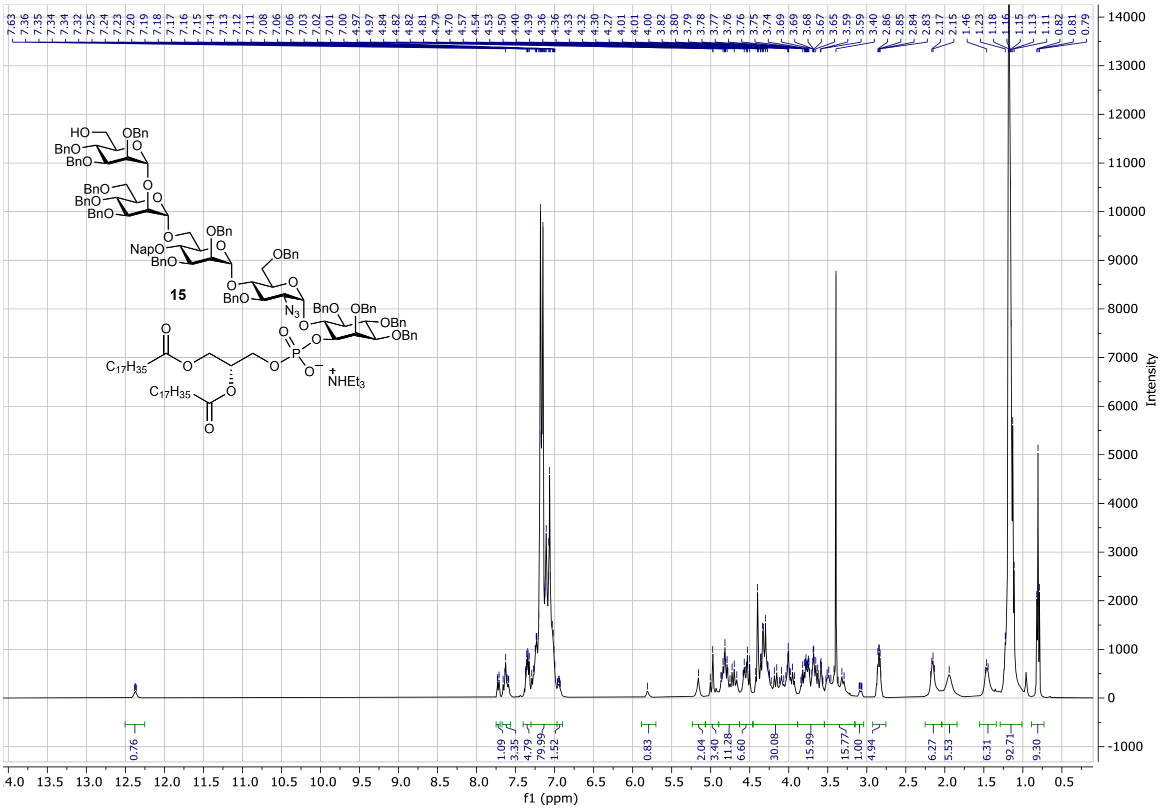

^{31}P NMR spectrum of lipidated *pseudo*-pentasaccharide **15** (162 Mhz, $CDCl_3$/MeOD (10:1)

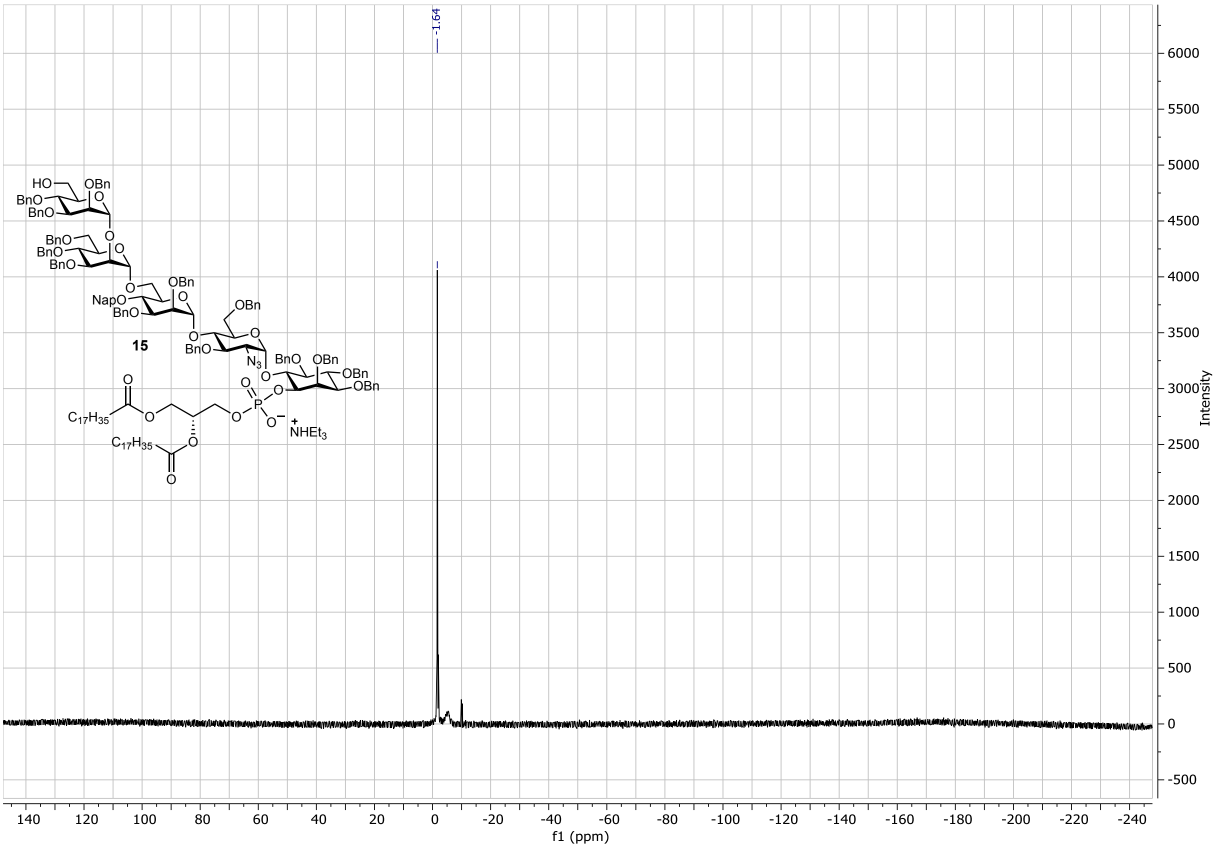

^{13}C NMR spectrum of lipidated *pseudo*-pentasaccharide **15** (101 Mhz, $CDCl_3$/MeOD (10:1)

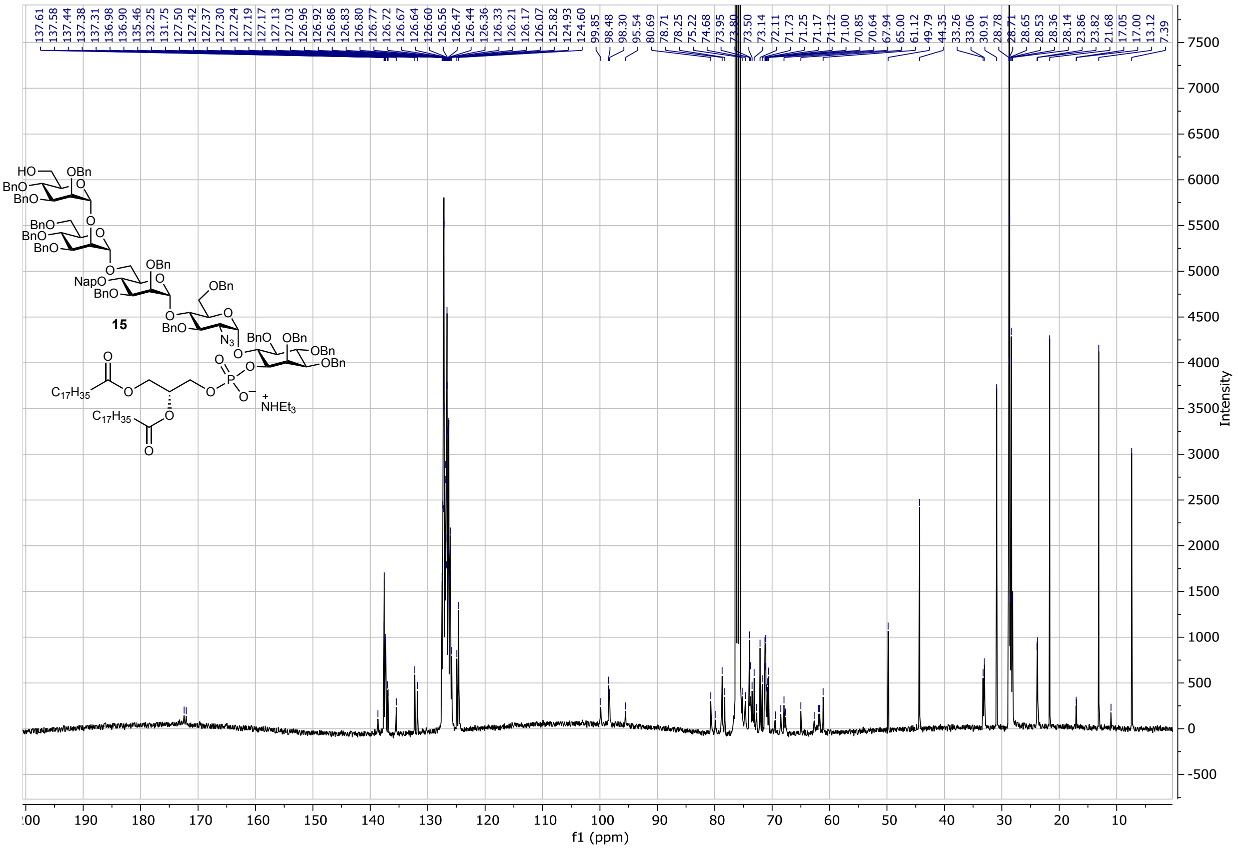

^{1}H NMR spectrum of bilipidated *pseudo*-pentasaccharide **16** (700 MHz, $CDCl_3$/MeOD, 10:1)

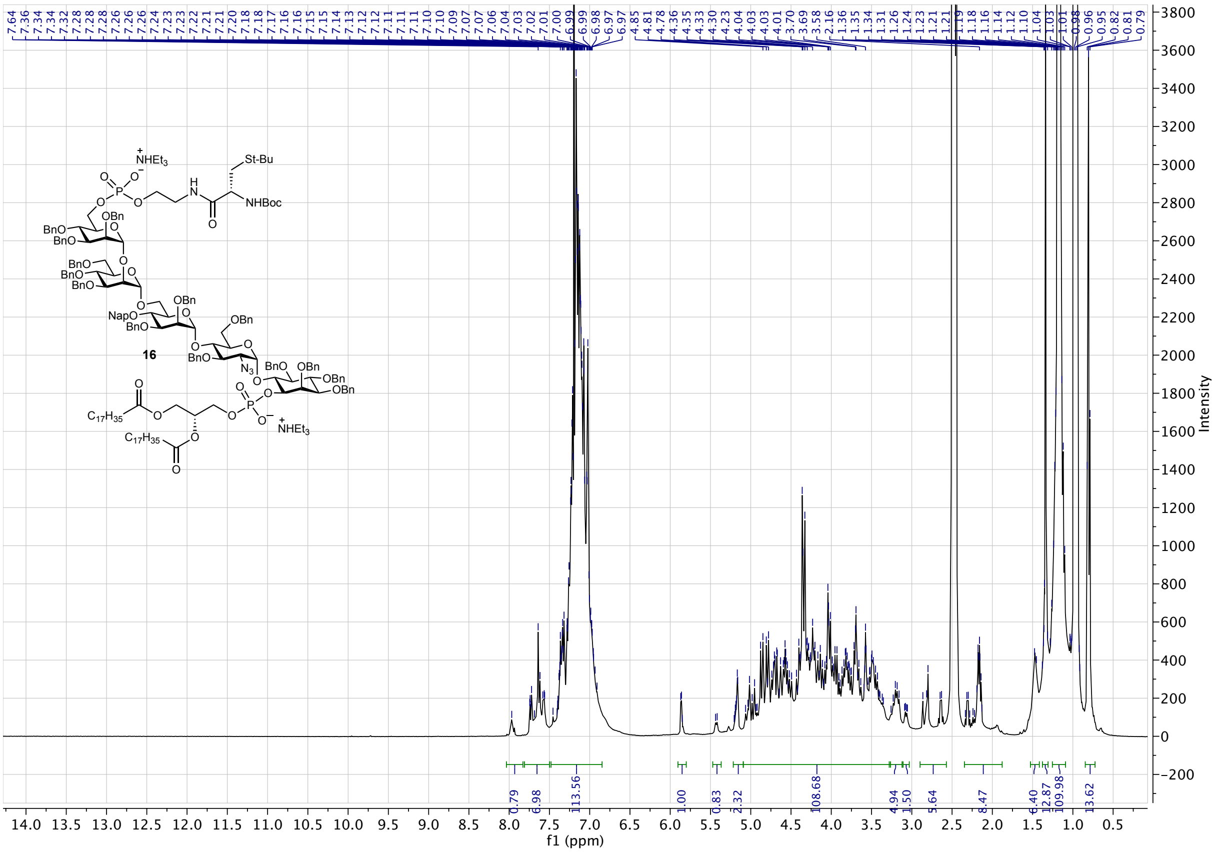

HSQC spectrum of bilipidated *pseudo*-pentasaccharide **16** (151 MHz, $CDCl_3$/MeOD, 10:1)

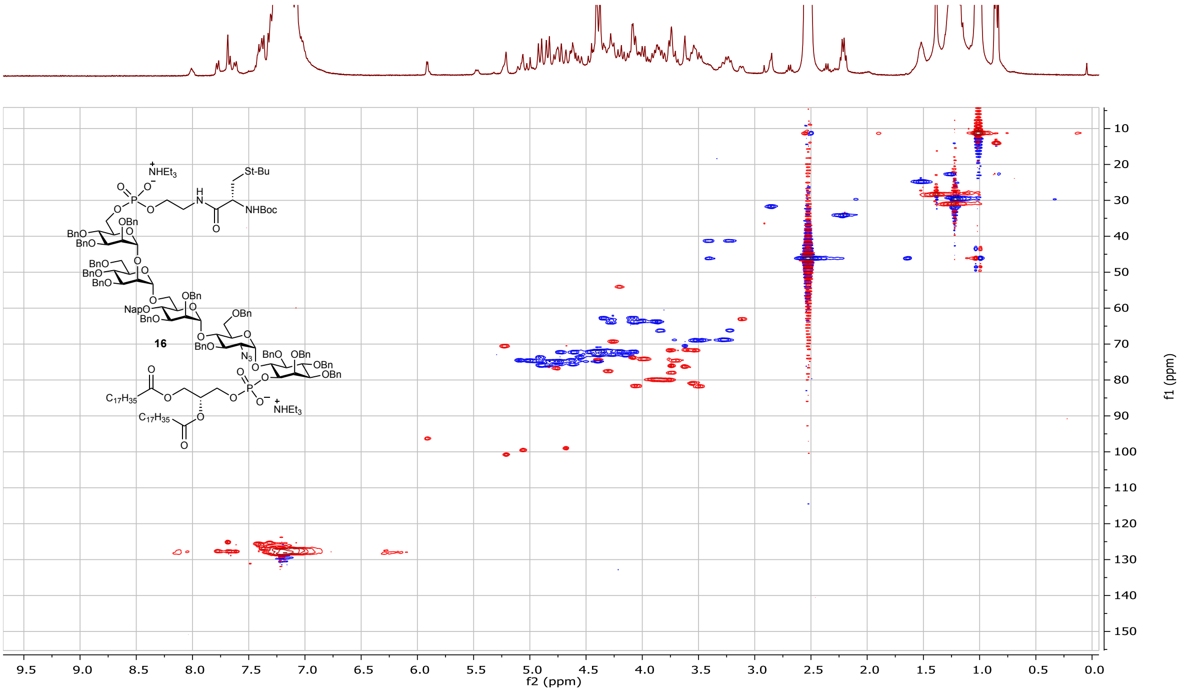

^{31}P NMR spectrum of bilipidated *pseudo*-pentasaccharide **16** (162 MHz, $CDCl_3$/MeOD, 10:1)

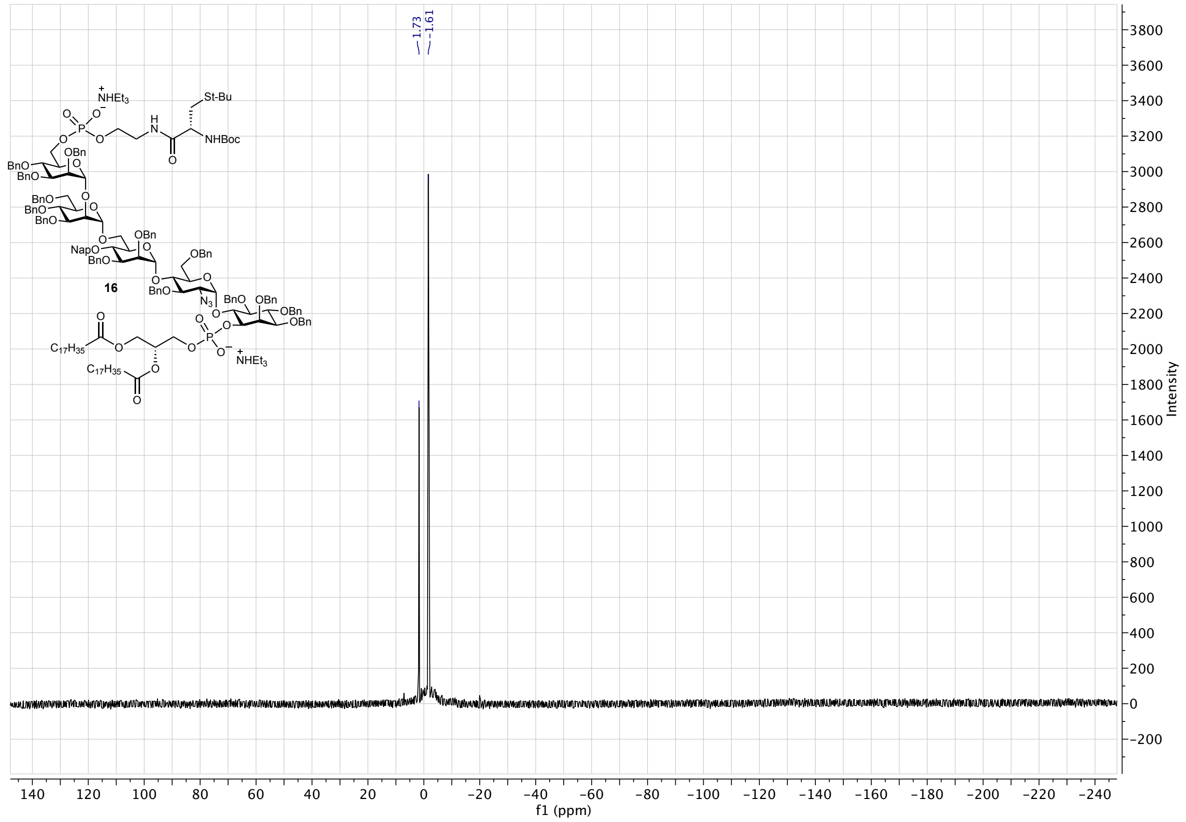

^{1}H NMR spectrum of GPI**2** (400 Mhz, $CDCl_3$/MeOD/D_2O (3:3:0.3)

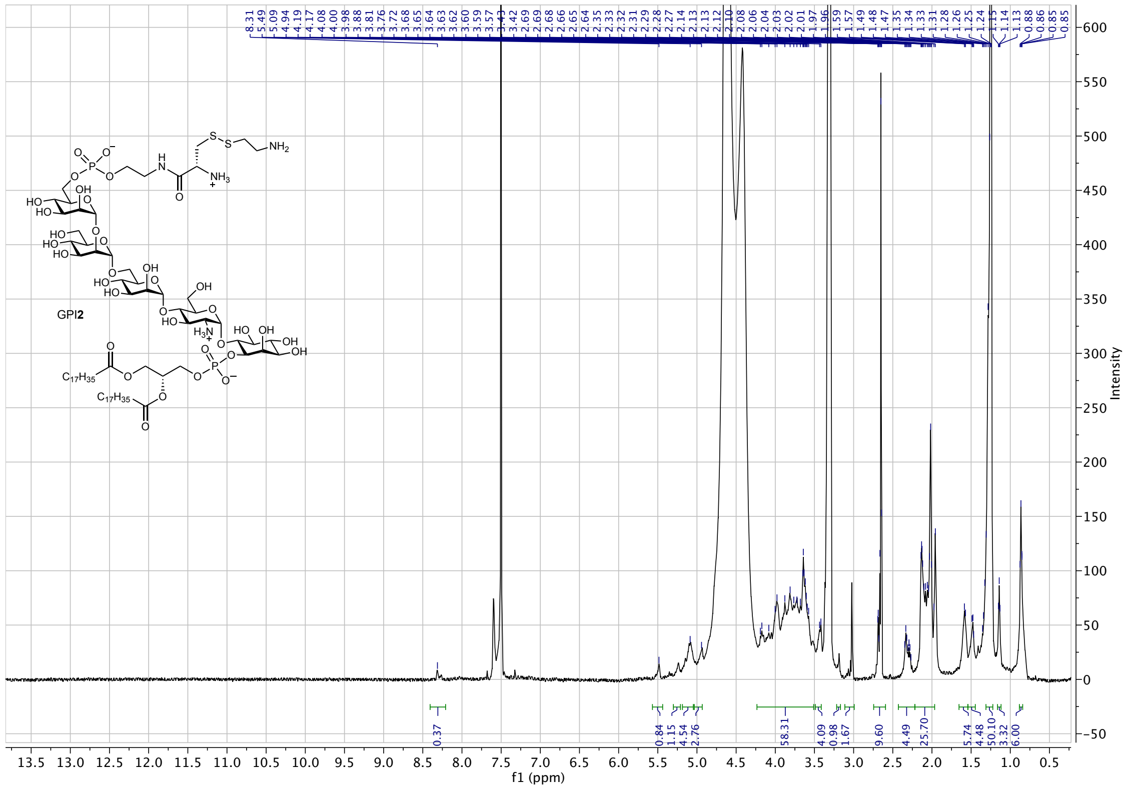

^{31}P NMR spectrum of GPI**2** (243 Mhz, $CDCl_3$/MeOD/D_2O (3:3:0.3)

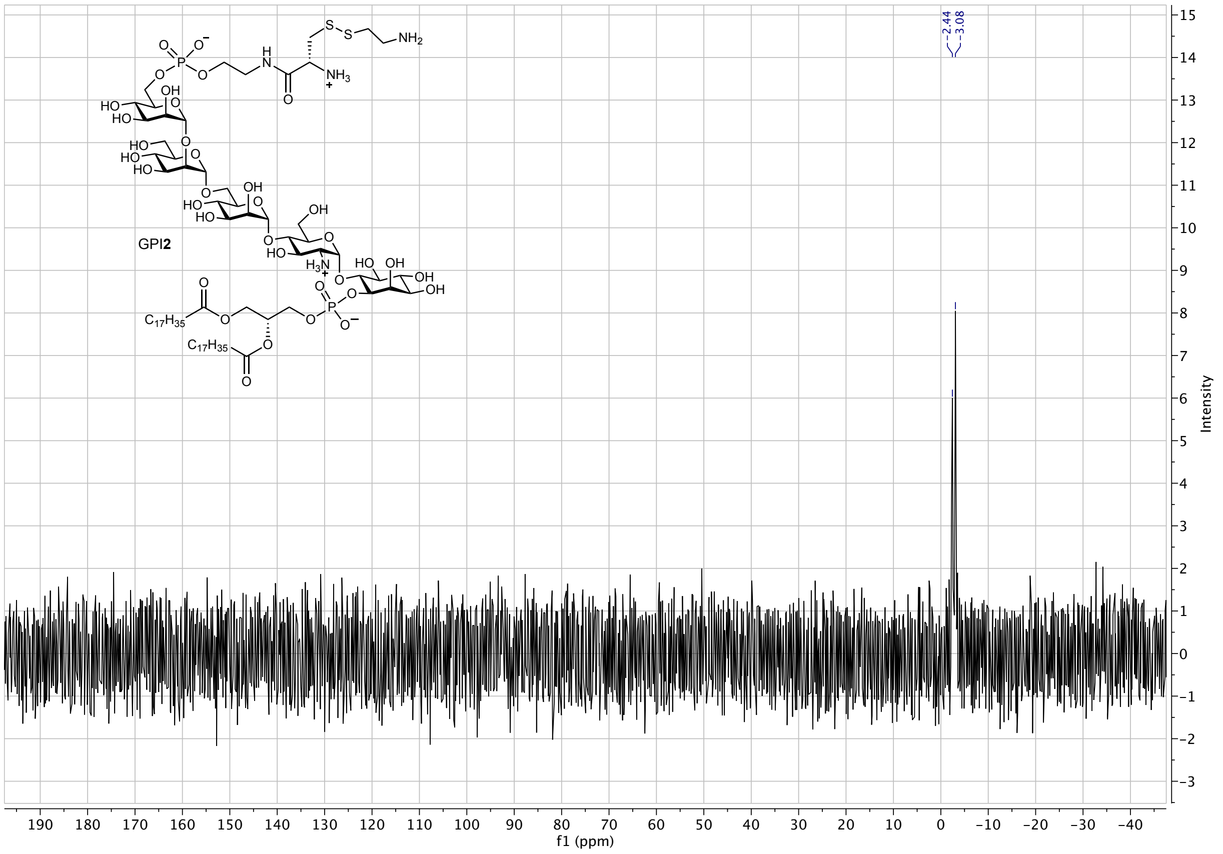

References

[1] a) S. L. Pedersen, A. P. Tofteng, L. Malik, K. J. Jensen, *Chem. Soc. rev.* **2012**, *41*, 1826-1844; b) J. M. Collins, K. A. Porter, S. K. Singh, G. S. Vanier, *Org. Lett.* **2014**, *16*, 940-943.

[2] P. Cherkupally, G. A. Acosta, L. Nieto-Rodriguez, J. Spengler, H. Rodriguez, S. N. Khattab, A. El-Faham, M. Shamis, Y. Luxembourg, R. Prohens, R. Subiros-Funosas, F. Albericio, *Eur. J. Org. Chem.* **2013**, *2013*, 6372-6378.

[3] T. Michels, R. Dölling, U. Haberkorn, W. Mier, *Org. Lett.* **2012**, *14*, 5218-5221.

[4] J. Zettler, V. Schütz, H. D. Mootz, *FEBS Lett.* **2009**, *583*, 909-914.

[5] Y.-H. Tsai, S. Gotze, I. Vilotijevic, M. Grube, D. Varon Silva, P. H. Seeberger, *Chem. Sci.* **2013**, *4*, 468-481.

[6] P. H. Seeberger, R. L. Soucy, Y. U. Kwon, D. A. Snyder, T. Kanemitsu, *Chem. Commun.* **2004**, 1706-1707.

[7] D. K. Baeschlin, A. R. Chaperon, L. G. Green, M. G. Hahn, S. J. Ince, S. V. Ley, *Chem. Eur. J.* **2000**, *6*, 172-186.

[8] X. Y. Liu, Y. U. Kwon, P. H. Seeberger, *J. Am. Chem. Soc.* **2005**, *127*, 5004-5005.

[9] P. J. Garegg, I. Lindh, T. Regberg, J. Stawinski, R. Strömberg, C. Henrichson, *Tetrahedron Lett.* **1986**, *27*, 4055-4058.

[10] C. F. W. Becker, X. Y. Liu, D. Olschewski, R. Castelli, R. Seidel, P. H. Seeberger, *Angew. Chem. Int. Ed.* **2008**, *47*, 8215-8219.
